# Tissue CD14^+^CD8^+^T-cells reprogrammed by myeloid cells and modulated by LPS

**DOI:** 10.1038/s41586-022-05645-6

**Published:** 2023-01-25

**Authors:** Laura J. Pallett, Leo Swadling, Mariana Diniz, Alexander A. Maini, Marius Schwabenland, Adrià Dalmau Gasull, Jessica Davies, Stephanie Kucykowicz, Jessica K. Skelton, Niclas Thomas, Nathalie M. Schmidt, Oliver E. Amin, Upkar S. Gill, Kerstin A. Stegmann, Alice R. Burton, Emily Stephenson, Gary Reynolds, Matt Whelan, Jenifer Sanchez, Roel de Maeyer, Clare Thakker, Kornelija Suveizdyte, Imran Uddin, Ana M. Ortega-Prieto, Charlotte Grant, Farid Froghi, Giuseppe Fusai, Sabela Lens, Sofia Pérez-del-Pulgar, Walid Al-Akkad, Giuseppe Mazza, Mahdad Noursadeghi, Arne Akbar, Patrick T.F. Kennedy, Brian R. Davidson, Marco Prinz, Benjamin M. Chain, Muzlifah Haniffa, Derek W. Gilroy, Marcus Dorner, Bertram Bengsch, Anna Schurich, Mala K. Maini

**Affiliations:** 1 Division of Infection & Immunity, Institute of Immunity & Transplantation, University College London, UK; 2 Division of Medicine, University College London, UK; 3 Institute of Neuropathology, University of Freiburg, Germany; 4 Department of Medicine, Imperial College London, UK; 5 Blizard Institute, Barts & The London School of Medicine & Dentistry, QMUL, UK; 6 Biosciences Institute, Faculty of Medical Sciences, Newcastle University, UK; 7 School of Immunology and Microbial Sciences, Kings College London, UK; 8 Division of Surgery, University College London, UK; 9 Liver Unit, Hospital Clinic, IDIBAPS and CIBEREHD, University of Barcelona, Spain; 10 Institute for Liver & Digestive Health, University College London, UK; 11 Center for Basics in NeuroModulation, Faculty of Medicine, and Signalling Research Centres BIOSS and CIBSS, University of Freiburg, Germany; 12 Signalling Research Centres BIOSS and CIBSS, University of Freiburg, Germany; 13 Department of Computer Science, University College London, UK; 14 Faculty of Medicine, Clinic for Internal Medicine II, Gastroenterology, Hepatology, Endocrinology, and Infectious Disease, University Medical Center Freiburg, Germany

## Abstract

The liver is bathed in bacterial products including LPS transported from the intestinal portal vasculature, but maintains a state of tolerance exploited by persistent pathogens and tumours^1,2,3,4^. The cellular basis mediating this tolerance, yet allowing a switch to immunity or immunopathology, needs to be better understood for successful immunotherapy of liver diseases. Here we show that a variable proportion of CD8^+^T-cells compartmentalised in the human liver co-stain for CD14 and other prototypic myeloid membrane proteins and are enriched in close proximity to CD14^hi^ myeloid cells in hepatic zone 2. CD14^+^CD8^+^T-cells preferentially accumulate within the donor pool in liver allografts, amongst hepatic virus-specific and tumour-infiltrating responses, and in cirrhotic ascites. CD14^+^CD8^+^T-cells exhibit increased turnover, activation and constitutive immunomodulatory features with high homeostatic IL-10/IL-2 production *ex vivo*, and enhanced antiviral/anti-tumour effector function upon TCR engagement. This CD14^+^CD8^+^T-cell profile can be recapitulated by acquisition of membrane proteins, including the LPS receptor complex, from mononuclear phagocytes, resulting in augmented tumour killing by TCR-redirected T-cells *in vitro*. CD14^+^CD8^+^T-cells express integrins and chemokine receptors favouring interactions with the local stroma, which can promote their induction through CXCL12. LPS can also increase the frequency of CD14^+^CD8^+^T-cells *in vitro* and *in vivo*, and skew their function towards the production of chemotactic and regenerative cytokines. Thus, bacterial products in the gut-liver axis and tissue stromal factors can tune liver immunity by driving myeloid instruction of CD8^+^T-cells with immunomodulatory capacity.

The highly tolerogenic properties of the liver are exemplified by the successful transplantation of this organ across MHC barriers. Homeostatic downregulation of immunity in the liver at steady-state is underscored by its predilection to harbour persistent pathogens and tumours, although this can be overridden during resolution of hepatic viral infection, transplant rejection or inflammatory liver diseases. The cellular mechanisms mediating hepatic immunoregulation, whilst allowing the switch to immunity or immunopathology, are poorly understood^1,2^. As the liver receives the majority of its blood supply from the portal vein, it is heavily exposed to bacteria and bacterial products such as lipopolysaccharide (LPS), able to translocate the gut and permeate the liver through the extensive fenestrated sinusoidal vasculature^2,3,4^. The gut microbiome and LPS content can influence the progression of liver diseases including hepatocellular carcinoma (HCC), but the cellular basis for this connection also remains ill-defined^3,5^. We recently described a population of tissue-resident memory CD8^+^T-cells (CD8^+^T_RM_) in the human liver and noted that some express CD14^6^, a component of the LPS receptor usually expressed on myeloid cells. Here we investigate the unique features of CD14^+^CD8^+^T-cells within the human liver, providing a mechanistic link between the stromal/myeloid network, bacterial LPS, and hepatic immunity.

## CD14^+^CD8^+^T-cells in human liver

**Extended Data Figure 1:**
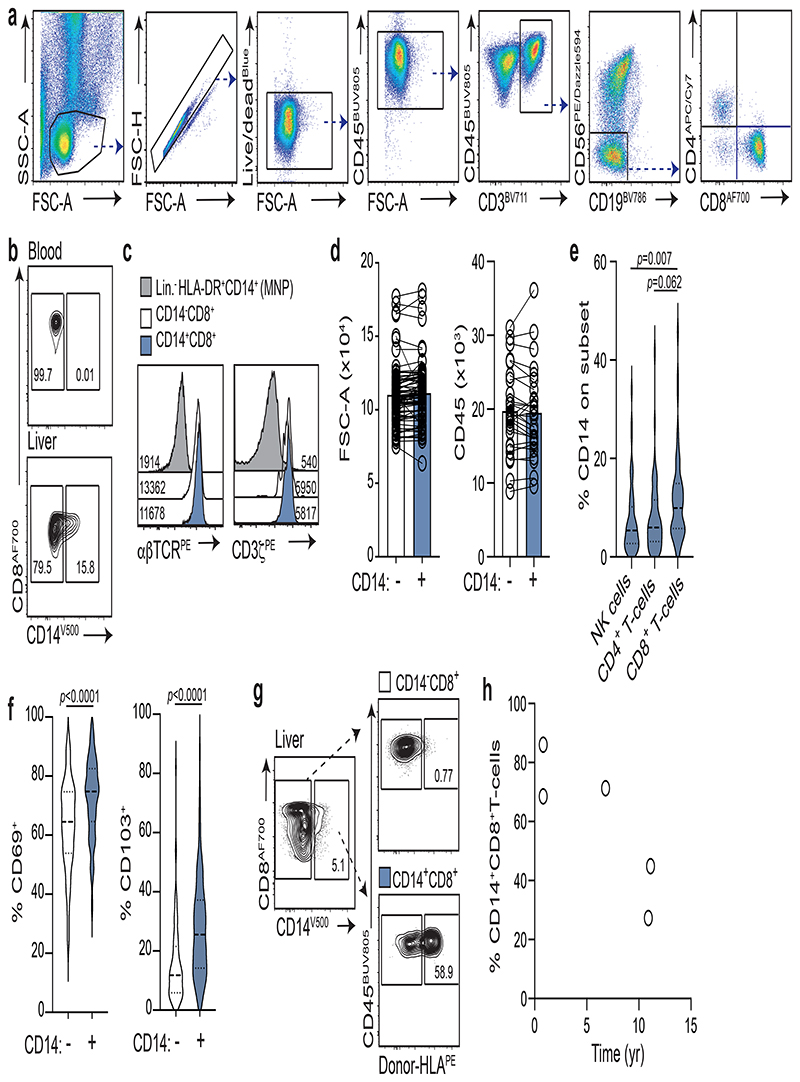
Confirmation of CD14^+^CD8^+^T-cells in human liver. **a** Flow cytometric gating strategy defining CD14^+^CD8^+^T-cells in human liver with sequential exclusion of: debris and MNP by forward (FSC-A) and side scatter (SSC-A), doublets, dead cells, CD45^-^, CD3^-^, CD56^+^, CD19^+^, and CD3^+^CD4^+^T-cells. **b** Representative flow cytometric staining of peripheral and intrahepatic CD14^+^CD8^+^T-cells (using a different clone, and company than example shown in Fig.1a). **c** Representative histograms of αβTCR and CD3ζ expression on intrahepatic CD14^-^CD8^+^T-cells (black outline), CD14^+^CD8^+^T-cells (blue filled) and classical MNP (large SSC-A, Lin^-^HLA-DR^+^CD3^-^CD14^+^; grey filled). **d** Summary data: cell size using FSC-A (n=80) and CD45 expression (n=28) on CD14^-^CD8^+^T-cells and CD14^+^CD8^+^T-cells. **e** CD14 percentage on CD3^-^ CD56^+^ NK cells, CD3^+^CD8^-^CD4^+^T-cells and CD3^+^CD4^-^CD8^+^T-cells from the same donors (n=28). **f** Summary data: percentage of CD69 (n=89) and CD103 (n=96) on intrahepatic CD14^-^CD8^+^T-cells or CD14^+^CD8^+^T-cells. **g** Representative plots showing the frequency of donor-derived (donor-HLA^+^) or recipient-derived (donor-HLA^-^) CD14^-^CD8^+^T-cells and CD14^+^CD8^+^T-cells. **h** Frequency of donor-derived CD14^+^CD8^+^T-cells in liver allografts explanted after 8 months-11 yrs from HLA-haplotype disparate recipients (n=5) using the gating strategy in Fig.1h. Each dot (or pair of dots) represent(s) a study participant, processed, stained and analysed independently; bars represent mean. **e** and **f** solid lines: median, dotted lines: IQR. *p*-values determined using either a Kruskal-Wallis test (ANOVA) with Dunn’s multiple comparisons test **e**; or two-tailed Wilcoxon *t* Test **d,f**.

**Figure 1:**
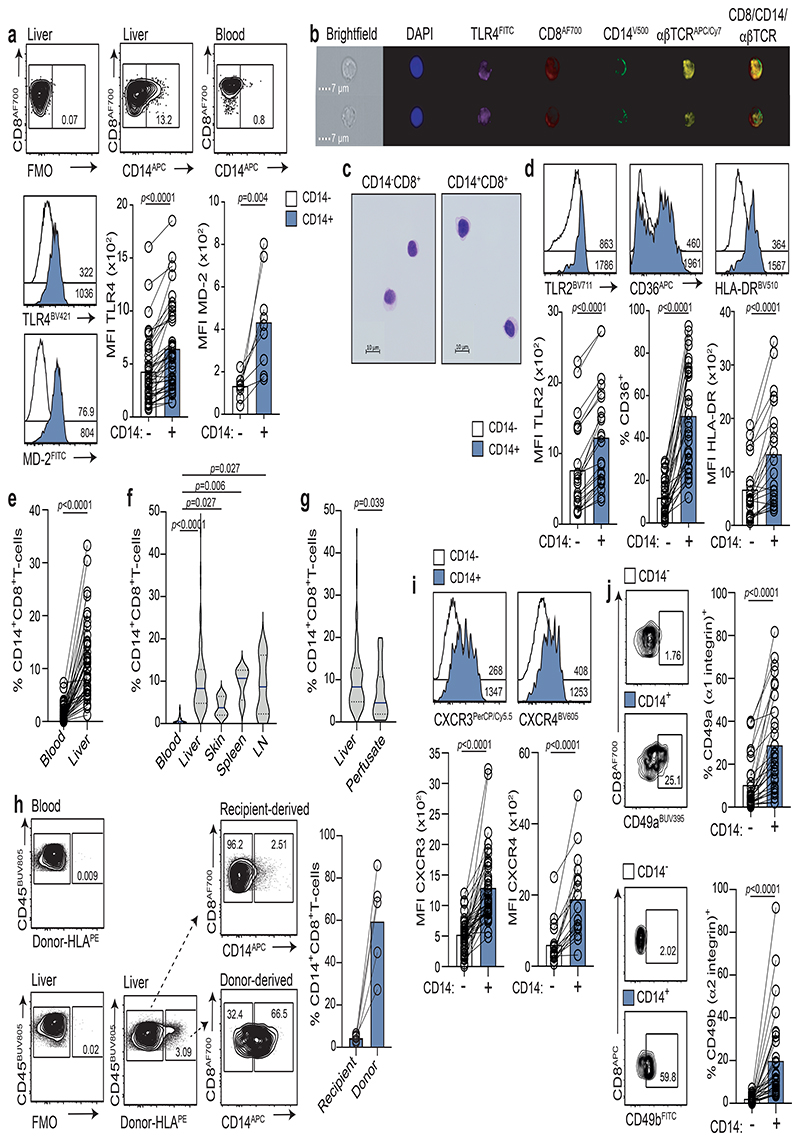
CD8^+^T-cells expressing CD14 in human liver. **a** Representative flow cytometry plots: human peripheral and intrahepatic CD8^+^T-cells stained with REAfinity anti-CD14 mAb compared to FMO control (gating strategy, Extended Data Fig.1a), and assessment of intrahepatic CD14^-^CD8^+^T-cell (black outline) and CD14^+^CD8^+^T-cell (blue filled) expression (MFI) of TLR4 (n=39) and MD-2 (n=10). **b** Single cell images of intrahepatic CD8^+^T-cells co-stained with αβTCR, CD14 and TLR4 (ImageStreamX MKII; 60x; representative of three samples). **c** Representative cytospins:FACS-sorted intrahepatic CD14^-^CD8^+^ and CD14^+^CD8^+^T-cells (representative of 2 samples). **d** Assessment of intrahepatic CD14^-^CD8^+^T-cell and CD14^+^CD8^+^T-cell TLR2 (n=23), CD36 (n=35) and HLA-DR (n=22) expression. Frequency of CD14^+^CD8^+^T-cells in **e** paired blood and non-diseased liver tissue from the same donor (n=46), and **f** blood (n=97), explanted/resected liver tissue (n=106), skin biopsies (n=6), splenic tissue (n=7), freshly isolated lymph nodes (n=9) and **g** explanted/resected liver tissue (n=106) compared to transplant vascular perfusates (n=25). **h** CD14^+^CD8^+^T-cell longevity in liver allografts explanted after 8months-11yrs from HLA-haplotype disparate recipients (n=5). HLA staining distinguishing donor- or recipient-derived leukocytes in blood and intrahepatic leukocytes; plots and summary data of CD14 expression on recipient-derived (donor-HLA^-^) and donor-derived (donor-HLA^+^) CD8^+^T-cells. Representative plots and summary data (MFI) of **i** CXCR3 (n=34) and CXCR4 (n=14) and percentage of **j** CD49a (integrin α1β1; n=28) and CD49b (integrin α2 n=17) on intrahepatic CD14^-^CD8^+^T-cells or CD14^+^CD8^+^T-cells. Each dot (or pair of dots) represent(s) a study participant, processed, stained and analysed independently; bars represent mean. **f** and **g** solid lines: median, dotted lines: IQR. *p*-values determined using either a Kruskal-Wallis test (ANOVA) with Dunn’s multiple comparisons test **f**; two-tailed Wilcoxon *t* Test **a, d-e, i-j**; or two-tailed Mann-Whitney *U* Test **g**.

We observed that a variable fraction of CD8^+^T-cells compartmentalised within human liver tissue stained for CD14, TLR4 and myeloid differentiation factor 2 (MD-2), components of the LPS receptor that are typically found on myeloid cells (gating strategy with doublet exclusion, Extended Data Fig.1a,b Fig.1a, using a REAfinity™ Fc-engineered antibody and FcR blocking step to exclude non-specific FcR staining). We further excluded the possibility that CD14^+^CD8^+^T-cells represented T-cell/monocyte doublets^7^ or atypical myeloid cells by visualising single cells co-staining for αβT-cell receptor (TCR), CD8, CD14 and TLR4 by imaging flow cytometry (Fig.1b) and by confirming CD14-expressing CD8^+^T-cells were equivalent in size, morphological appearance and expression of αβTCR, CD45 and CD3ζ-chain to conventional intrahepatic CD8^+^T-cells without CD14 (Fig.1c, Extended Data Fig.1c,d). CD14^+^CD8^+^T-cells were similarly enriched for other surface receptors highly expressed by myeloid cells including TLR2, CD36 and HLA-DR (Fig.1d). CD14^+^CD8^+^T-cells accounted for a highly variable fraction of intrahepatic CD8^+^T-cells (median 8.3%, max. 45.7%), whereas they were often barely detectable in paired peripheral blood samples from the same donors (Fig.1e,f). CD14 tended to be detectable on a higher proportion of CD8^+^T-cells than CD4^+^T-cells or NK cells in the same liver samples (Extended Data Fig.1e). CD14^+^CD8^+^T-cells were also found at higher frequencies in healthy human skin and lymphoid organs (spleen and lymph nodes) compared to blood (Fig.1f).

The strikingly reduced proportion of CD8^+^T-cells staining for CD14 in the circulation pointed to this being a feature of tissue-residency; consistent with this, liver CD14^+^CD8^+^T-cells expressed high levels of the prototypic residency markers CD69 and CD103^6^ (Extended Data Fig.1f). To further investigate the propensity for CD14 to preferentially accumulate on CD8^+^T_RM_ we examined MHC-mismatched liver allografts, distinguishing donor and recipient populations by their disparate HLA-haplotype staining. In this setting we recently showed that a small pool of donor-derived CD8^+^T_RM_ can persist for more than a decade^8^, suggesting this fraction has specialised adaptations rendering it resistant to removal by pre-transplant liver perfusion and able to undergo homeostatic proliferation to maintain its progeny long term. Global CD8^+^T_RM_ (defined by CD69/CD103 co-expression) are flushed out during liver transplant donor organ perfusion at similar frequencies to those isolated from digested liver tissue, in line with a predominantly intravascular localisation^6,9^; by contrast, CD14^+^CD8^+^T-cells were less abundant in pre-transplantation perfusates than in tissue digests (Fig.1g). Consistent with reduced removal by perfusion, CD14^+^CD8^+^T-cells were strikingly enriched amongst the small pool of long-lived donor-derived lymphocytes in HLA-mismatched allografts, ranging from 86% at eight months to 27-45% ˜11 years post-transplantation (Fig.1h, Extended Data Fig.1g,h). By contrast, recipient T-cells, which can increase expression of CD69 but not CD103 or CXCR3 upon infiltrating liver allografts^8^, were not able to acquire substantial CD14 expression, even after 11 years (Fig.1h, Extended Data Fig.1h). These data indicated that CD14 expression was preferentially induced on a well-tethered proliferative CXCR3^hi^ tissue-resident population but could not be imprinted on HLA-mismatched peripheral CD8^+^T-cells infiltrating the liver vasculature.

To probe a role for chemokine/stromal-dependent signals, we examined whether CD14-staining CD8^+^T-cells had differential expression of prototypic liver-homing chemokine receptors and integrins. CD14^+^CD8^+^T-cells freshly isolated from liver tissue were selectively enriched for expression of CXCR3 (promoting CXCL10 signals from liver sinusoidal endothelial cells and hepatocytes^10^) and CXCR4 (favouring binding to CXCL12 [stromal cell-derived factor 1] produced by endothelial and stromal cells^11,12^) (Fig.1i). CD14^+^CD8^+^T-cells also expressed markedly more of the extracellular matrix (ECM)-binding integrins CD49a and CD49b (Fig.1j). Taken together, the allograft and homing profile of CD14^+^CD8^+^T-cells suggested they were selectively tethered within the liver stromal network, dictating the local cues they receive.

## CD14 marks myeloid-instructed CD8^+^T-cells

**Extended Data Figure 2:**
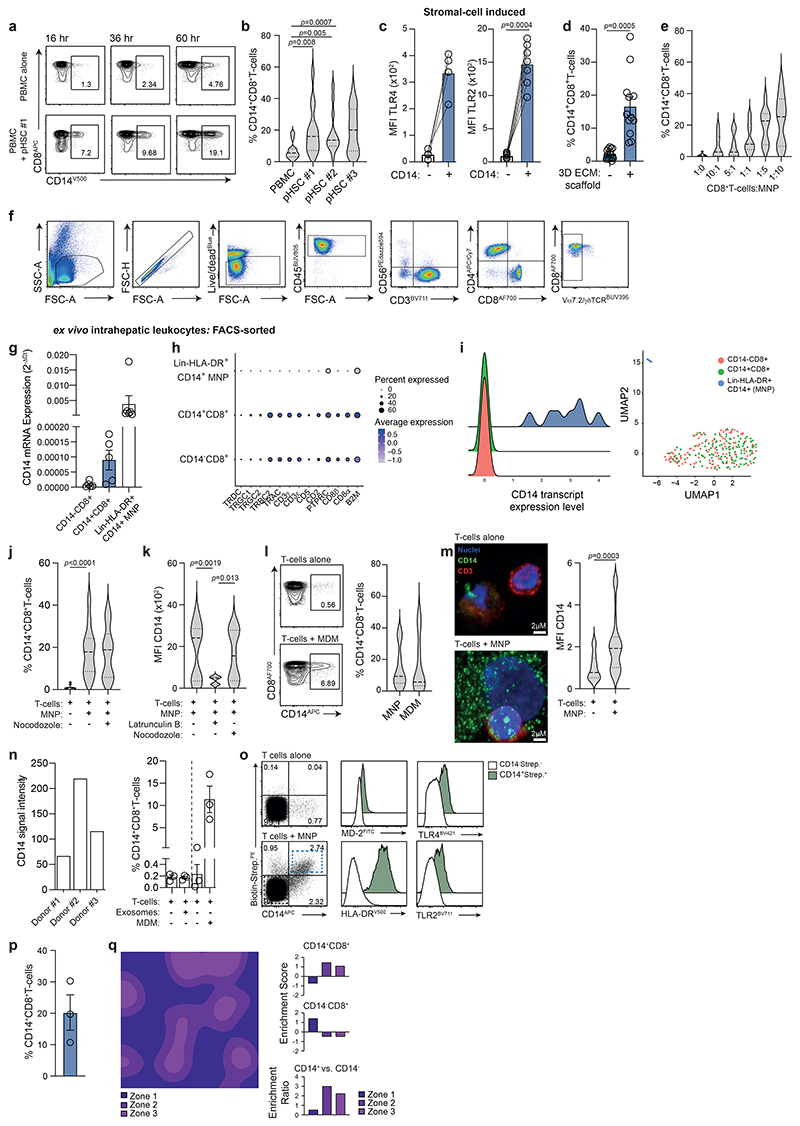
Residency and transcriptional features of CD14^+^CD8^+^T-cells. **a** Representative plots: %CD14 on peripheral CD8^+^T-cells after PBMC and pHSC co-culture (representative of four PBMC donors with 3 pHSC donors). **b** Summary data: *in vitro* induction of CD14^+^CD8^+^T-cells after PBMC and pHSC co-culture (4d; PBMC donors used with: pHSC#1 n=15; pHSC#2 n=30; and pHSC#3 n=24). **c** Summary data: MFI of TLR4 (n=4) and TLR2 (n=7) of pHSC-induced CD14^-^CD8^+^T-cells (black outline) or CD14^+^CD8^+^T-cells (blue filled). **d** %CD14 on peripheral CD8^+^T-cells after PBMC cultured ± 3D de-cellularised human liver ‘extracellular matrix scaffolds’ (3d; n=12 PBMC donors; 3 experiments). **e** Frequency of peripheral CD14^+^CD8^+^T-cells after co-culture of purified CD8^+^T-cells with a varied ratio of autologous MNP (4d; n=11). **f** Flow cytometric gating strategy used to isolate *ex vivo* or stromal-cell induced CD14^+^CD8^+^T-cells with the sequential exclusion of: debris and MNP by forward (FSC-A) and side scatter (SSC-A), doublets, dead cells, CD45^-^, CD3^-^, CD56^+^, CD3^+^CD4^+^T-cells and γδT-cells/Vα7.2^+^T-cells (strategy used for experiments shown in Fig.4b and Extended Data Fig.2g-i, 3g and 4b). **g** Assessment of CD14 mRNA by RT-PCR (relative to 18S control) of FACS-sorted Lin^-^HLA-DR^+^CD3^-^CD14^+^ MNP, *ex vivo* intrahepatic CD14^-^CD8^+^T-cells and CD14^+^CD8^+^T-cells (n=5). **h** Percentage expression and relative levels of T-cell genes by scRNAseq transcriptome analysis of FACS-sorted intrahepatic CD14^-^CD8^+^T-cells, CD14^+^CD8^+^T-cells or Lin^-^HLA-DR^+^CD3^-^ CD14^+^ MNP (n=2 liver samples). **i** Histogram plot: CD14 mRNA transcript expression and UMAP depicting the transcriptomic profile of FACS-sorted intrahepatic CD14^-^CD8^+^T-cells (red), CD14^+^CD8^+^T-cells (green) or Lin^-^HLA-DR^+^CD3^-^CD14^+^ MNP (blue; n=2 samples). **j** Frequency of peripheral CD14^+^CD8^+^T-cells after co-culture of purified CD8^+^T-cells ± MNP supplemented with nocodozole (4d; n=10). **k** MFI of CD14 on CD14^+^CD8^+^T-cells after co-culture ± MNP supplemented with latrunculin B or nocodozole (n=10). **l** Representative and summary data: Percentage CD14 expression on CD8^+^T-cells post co-culture ± MDM or MNP isolated from the same donor (n=9). **m** Representative confocal images of purified CD8^+^T-cells co-cultured with MDM (4d; images representative of 2 donors) and summary data: comparison of MFI of CD14 on the T-cell population post culture with media alone or autologous MDM (defined by identification of the cell boundary segmented using the extent of CD8^+^ expression). **n** (left panel) CD14 signal intensity of MDM-derived exosomes from 3 donors and (right panel) percentage CD14 after co-culture of purified CD8^+^T-cell with concentrated MDM-derived exosomes or the autologous MDM from which exosomes were derived (4d; n=3). **o** Representative plots: MFI of MD-2, TLR4, HLA-DR and TLR2 on peripheral T-cells co-expressing (or lacking) CD14 and the biotin-streptavidin complex after exposure to biotin-labelled MNP. **p** Summary data: frequency of intrahepatic CD14^+^CD8^+^T-cells as determined by imaging mass cytometry (IMC) (mean ±S.E.M. of percentage expression in two distinct regions of interest/liver). **q** Representative IMC image of macroscopically healthy liver tissue sections (2 regions of interest captured from n=3 sections) as shown in Fig.2n with the demarcation of three liver zones defined by intensity of CYP1A2 staining (scale bar: 200µM; magnification: 50µM); enrichment scores of the zonal localisation of intrahepatic CD14^-^ CD8^+^T-cells and CD14^+^CD8^+^T-cells. Each dot (or pair of dots) represent(s) a study participant, processed, stained and analysed independently; bars represent mean and error bars (where appropriate) represent ± S.E.M. **b, e, j-m** solid lines: median, dotted lines: IQR. *p*-values determined using either a Kruskal-Wallis test (ANOVA) with Dunn’s multiple comparisons test **b** (compared to media alone), **j, k** (compared to MNP + T-cells); or two-tailed Wilcoxon *t* Test **c-d, l, m**.

**Figure 2:**
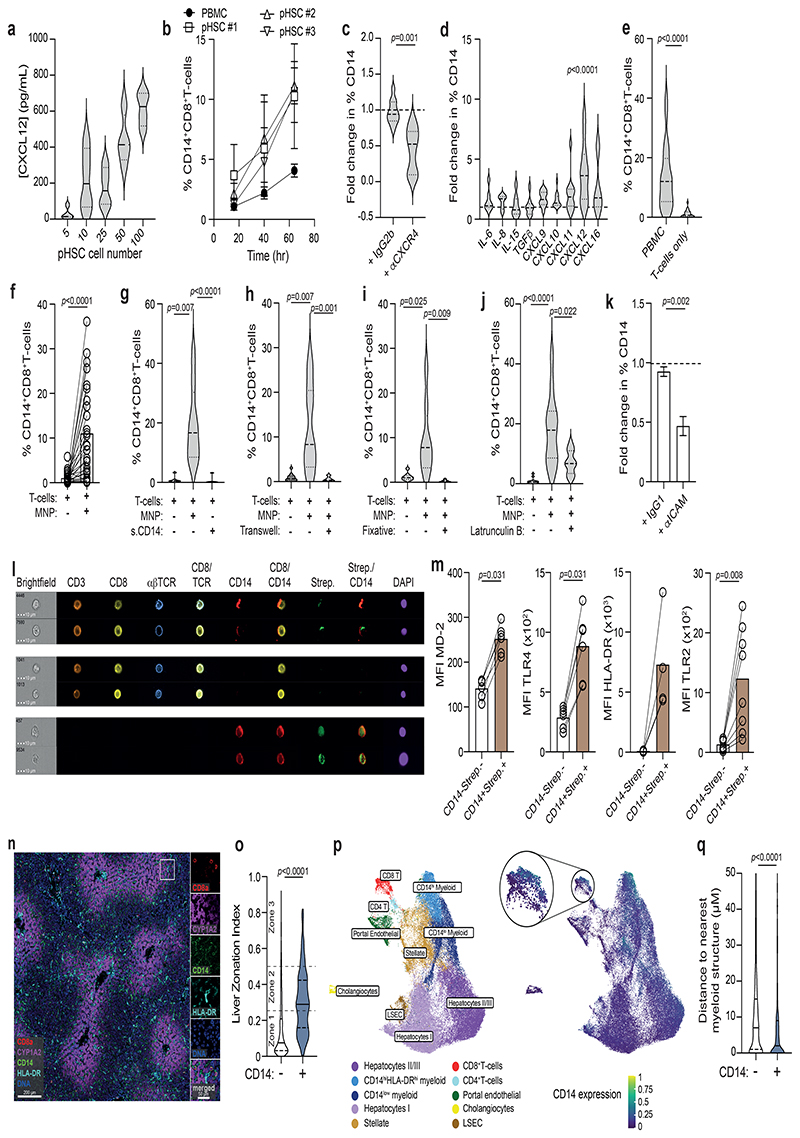
Derivation of CD14^+^CD8^+^T-cells. **a** pHSC production of CXCL12 (pg/mL; n=3; after 4 d culture with increasing cell numbers). **b** %CD14 staining on CD8^+^T-cells over time after co-culture of PBMC (n=4 donors) ±pHSC (derived from n=3 donors); mean±S.E.M. **c** and after 4d co-culture ±anti-CXCR4 or isotype-matched control (n=11). **d** Fold change in %CD14^+^CD8^+^T-cells in PBMC supplemented with recombinant IL-6, IL-8, IL-15, TGFβ, CXCL9, CXCL10, CXCL11, CXCL12 or CXCL16 (n=7,7,8,16,12,14,12,24,8 respectively) compared to media alone. **e** %CD14^+^CD8^+^T-cells after co-culture of PBMC (n=27) or magnetic-bead isolated CD8^+^T-cells (n=27) with pHSC (4d). %CD14^+^CD8^+^T-cells after co-culture of purified CD8^+^T-cells ± **f** freshly isolated autologous MNP (1:1 ratio; n=27), **g** soluble CD14 (s.CD14; n=12), **h** MNP ± separated by 0.4 µM transwell insert (n=8), **i p**rior MNP fixation with 4% paraformaldehyde (n=8), **j** MNP ± latrunculin B (n=10) and **k** fold change of MNP effect on % CD14^+^CD8^+^T-cells with anti-ICAM-1 or isotype-matched control (n=15). **l** Representative single cell images of biotin-labelled autologous MNP cultured 4d alone (bottom 2 rows) or with magnetic-bead isolated peripheral CD8^+^T-cells (top 4 rows), all co-stained with αβTCR, CD3, CD8, CD14 and streptavidin mAb (ImageStreamX MKII; 60x, representative of 3 samples). **m** MFI of MD-2, TLR4, HLA-DR and TLR2 (n=6,6,4,8 respectively) on peripheral T-cells ±CD14 and streptavidin staining after exposure to biotin-labelled MNP. **n** Representative imaging mass cytometry (IMC) of macroscopically healthy liver sections (representative of 3 samples), co-stained for CD8 (red), CYP1A2 (purple), CD14 (green), HLA-DR (cyan) and DNA (blue) (scale bar:200µM; magnification:50µM). **o** Summary data: distribution of CD14^-^CD8^+^T-cells and CD14^+^CD8^+^T-cells across liver zones (defined by expression of CYP1A2 intensity, see Extended Data Fig.2q). **p** UMAP of 72,000 single cells clustered by expression profile (left) and scaled CD14 intensity, with magnification of the CD8^+^T-cell cluster (right). **q** Summary data: distance of CD14^-^CD8^+^T-cells and CD14^+^CD8^+^T-cells to nearest lba1+ cell. Each dot represents a PBMC or CD8^+^T-cell/MNP donor, processed, stained and analysed independently; bars represent mean. **a, c-j, o, q** solid lines:median, dotted lines:IQR. *p*-values determined using Kruskal-Wallis test (ANOVA) with Dunn’s multiple comparisons test **d** (compared to media alone), **g**-**j** (compared to MNP + T-cells condition); or two-tailed Wilcoxon *t* Test **c, e**-**f, k, m, o** and **q**.

Next, we tested whether hepatic stromal cells (activated stellate cells/myofibroblasts), that produce ECM proteins and CXCL12 (relevant ligands for CD49a/b and CXCR4 respectively)^11^, could play a role in instructing CD8^+^T-cells to express CD14. Primary hepatic stellate cells (pHSC) were isolated from resected liver tissue from three donors and differentiated into activated myofibroblasts, able to release CXCL12 (Fig.2a). Transdifferentiated pHSC promoted CD8^+^T-cells co-staining for CD14 within PBMC co-cultures in a time-dependent manner, accompanied by an increase in expression of TLR4 and TLR2 (Fig.2b, Extended Data Fig.2a-c). Interestingly, only a proportion of PBMC acquired CD14 *in vitro*, with the mean and range roughly mimicking that seen e*x vivo* from human liver (Fig.2b, Extended Data Fig.2b). Stellate cell induction of CD14 on CD8^+^T-cells was significantly reduced by blocking the CXCL12 receptor CXCR4 (Fig.2c). The role of CXCL12 was confirmed by its capacity to recapitulate the increase in CD14 expression on CD8^+^T-cells in the absence of stellate cells, an effect not seen with the addition of other candidate prototypic liver-derived cytokines/chemokines (Fig.2d). Culture in three-dimensional de-cellularised liver scaffolds^13^ likewise induced CD14^+^CD8^+^T-cells from PBMC (Extended Data Fig.2d), implicating a role for the ECM, which is known to contain TLR4-binding proteins as well as retaining chemokines like CXCL12^14^.

*In vitro* induction of CD14^+^CD8^+^T-cells was abrogated when pHSC were co-cultured with isolated CD8^+^T-cells rather than PBMC (Fig.2e). This raised the possibility that CD14 on CD8^+^T-cells in the liver could be transferred from other cell types like mononuclear phagocytes (MNP), recently postulated to deposit CD14^+^ ‘particles’ on T-cells following *in vivo* conjugate formation^7^. Consistent with this, addition of purified monocytes to T-cell/stellate cell co-cultures recapitulated, in a dose-dependent manner, the expansion of CD14^+^CD8^+^T-cells observed with whole PBMC, whereas the addition of soluble CD14 (at physiological concentrations) did not (Fig.2f,g, Extended Data Fig.2e). In support of CD14^+^CD8^+^T-cells predominantly acquiring CD14 protein rather than transcribing it, examining sorted intrahepatic CD14^+^CD8^+^T-cells (gating strategy Extended Data Fig.2f) revealed that CD14 mRNA was at the threshold of detection by RT-PCR (Extended Data Fig.2g) whilst single-cell RNA sequencing (scRNA-Seq) identified classical CD8^+^T-cell transcripts (Extended Data Fig.2h) but no detectable CD14 or overlap with the transcriptional profile of MNP sorted from the same livers (Extended Data Fig.2h,i).

We investigated the mechanism by which CD8^+^T-cells acquire prototypic myeloid markers using autologous purified T-cells and MNP co-cultured in contact with pHSC. Separation of T-cells and MNP by transwell membranes abrogated the ability of MNP to transfer CD14 to CD8^+^T-cells (Fig.2h), pointing to a contact-dependent mechanism. Active transfer was supported by prior fixation of MNP preventing CD14 acquisition by CD8^+^T-cells (Fig.2i). Addition of latrunculin B to block the actin polymerisation required for immune synapse formation reduced both the percentage and intensity of CD14 staining on CD8^+^T-cells, whereas blockade of microtubule polymerisation (involved in lytic granule transport) using nocodazole did not (Fig.2j, Extended Data Fig.2j,k). LFA-1 is a key feature of liver T_RM_ allowing their patrolling function^15^ and the LFA-1/ICAM-1 axis is central to T-cell synapse formation; ICAM-1 blockade also decreased CD14 acquisition compared to the isotype control (Fig.2k).

To more closely mimic the myeloid landscape of the liver, we co-cultured CD8^+^T-cells with monocyte-derived macrophages (MDM) since hepatic macrophages (Kupffer cells) play a central role in dictating the function of liver T-cells^16,17^. MDM, like circulating monocytes, were able to transfer CD14 to CD8^+^T-cells by flow cytometry, with acquisition of punctate CD14 staining on individual CD8^+^T-cells (co-cultured with MDM) confirmed by confocal imaging (Extended Data Fig.2l,m). The previous transwell data argued against a role for exosomes (since these are 30-100 nM diameter, small enough to pass through the 400 nM pore inserts; Fig.2h). Exosome transfer was further excluded by specifically purifying CD14-containing exosomes from the supernatant of MDM derived from three donors (Extended Data Fig.2n, left panel) and showing that these were unable to recapitulate the transfer of CD14 to CD8^+^T-cells achieved by direct contact with their MDM (Extended Data Fig.2n, right panel). To further interrogate the process by which CD14 was acquired by CD8^+^T-cells, we incorporated a biotin-label into the plasma membrane of the myeloid cells prior to co-culture. Imaging cytometry confirmed the capture of CD14 from the biotin-labelled membrane of the myeloid cells by CD8^+^T-cells, with fluorescently-labelled streptavidin co-staining CD8^+^T-cells that had acquired CD14 and not those lacking CD14 (Fig.2l). Flow cytometric phenotyping of CD8^+^T-cells labelled with both CD14 and streptavidin revealed that CD8^+^T-cells had acquired the entire CD14/TLR4/MD-2 complex, along with additional prototypic MNP markers HLA-DR and TLR2 from the biotin-labelled myeloid cell membrane (Fig.2m, Extended Data Fig.2o).

To examine the topological relationship between CD14^+^CD8^+^T-cells and MNP *in vivo*, we visualised them in macroscopically healthy human liver sections using imaging mass cytometry. Images were segmented for single cell analysis and cell clustering carried out (2 regions of interest per liver from 3 donors, example image Fig.2n); quantitative high dimensional spatial profiling of liver sections identified a proportion of intrahepatic CD8^+^T-cells co-expressing CD14 (mean 20%, Extended Data Fig.2p), a frequency in line with the range we had quantified by flow cytometry. Whereas CD14^-^CD8^+^T-cells tended to accumulate in the periportal region (zone 1), CD14^+^CD8^+^T-cells had a significantly different liver zonation index, being relatively enriched in the midlobular zone 2, the region where hepatocytes maintain liver homeostasis^18^ (Fig2o, Extended Data Fig.2q). UMAP visualised a cluster with increased CD14 expression within CD8^+^T-cells as well as a cluster of CD14^hi^HLA-DR^hi^ MNP (Fig.2p). Critically, CD14^+^CD8^+^T-cells were observed in close proximity to CD14^hi^HLA-DR^hi^ MNP (Fig.2n), with quantitative analysis confirming they were significantly closer to the nearest MNP compared to the distance between CD14^-^CD8^+^T-cells and MNP (Fig.2q).

Taken together these data suggested that the population of CD14^+^CD8^+^T-cells identified *ex vivo* from human liver, with a distinct zonal distribution, represented the fraction that had acquired cellular material from CD14^hi^ myeloid cells, in a contact-dependent manner that was facilitated by CXCL12/stromal interactions.

## Functional profile of CD14^+^CD8^+^T-cells

**Extended Data Figure 3:**
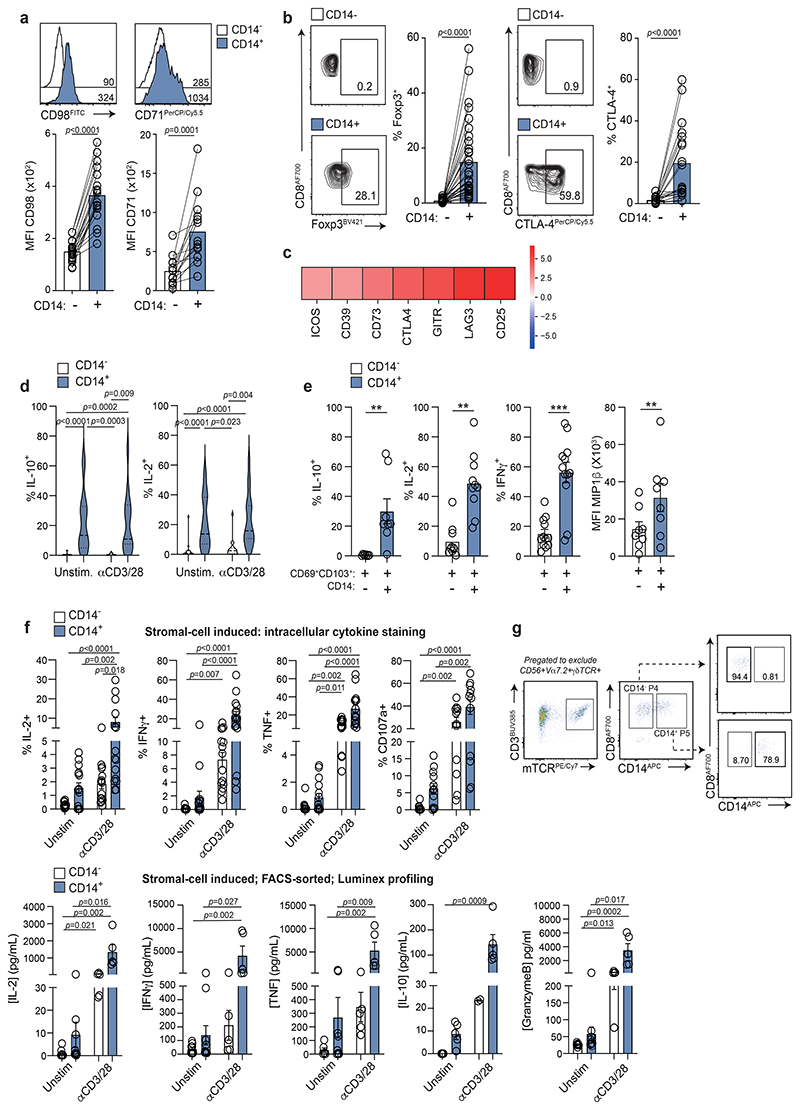
Nutrient transporter and immunomodulatory profile of intrahepatic CD14^+^CD8^+^T-cells and soluble mediator profile of stromal cell-derived CD14^+^CD8^+^T-cells. Representative flow cytometric plots and summary data: MFI expression of **a** CD98 (n=19) and CD71 (n=14) and **b** Foxp3 (n=32) and CTLA-4 (n=19) of *ex vivo* intrahepatic CD14^-^CD8^+^T-cell (black outline) and CD14^+^CD8^+^T-cells (blue filled). **c** Heatmap showing relative expression (fold change) of immunomodulatory markers on CD14^+^CD8^+^T-cells compared to their CD14^-^CD8^+^T-cell counterparts. **d** Assessment of intrahepatic CD14^+^CD8^+^T-cell intracellular cytokine production ± 4hr anti-CD3/CD28 stimulation in the presence of brefeldin-A: IL-10 (n=15) and IL-2 (n=19) **e** Assessment of CD14^+^CD8^+^T-cell production of IL-10 (n=15) and IL-2 (n=19), and IFNγ (n=19) andMIP1β (n=12) afte 4hr anti-CD3/CD28 stimulation further defined by co-expression of CD69 and CD103. **f** Summary data: peripheral stromal cell-induced CD14^-^CD8^+^T-cell and CD14^+^CD8^+^T-cell populations post-stimulation with anti-CD3/CD28 by flow cytometric intracellular cytokine staining for IL-2, IFNγ, TNF (n=15) and CD107a (n=12) (4hr; upper panel) and by supernatant luminex detection of secreted IL-2, IFNγ, TNF, IL-10 and granzyme B from FACS-sorted stromal cell-induced populations (n=5; 16hr; lower panel). **g** Example flow cytometry plots: gating strategy for the FACS-sorting of TCR-transduced CD8^+^T-cells with or without CD14 staining using the murineTCRβ (mTCR) region contained within the transgene. Each dot (or pair of dots) represent(s) a study participant, processed, stained and analysed independently; bars represent mean, error bars (where appropriate) represent ± S.E.M. **d** solid lines: median, dotted lines: IQR. *p*-values determined using either a Kruskal-Wallis test (ANOVA) with Dunn’s multiple comparisons test **d, f**; or a two-tailed Wilcoxon *t* Test **a-b, e**.

**Figure 3:**
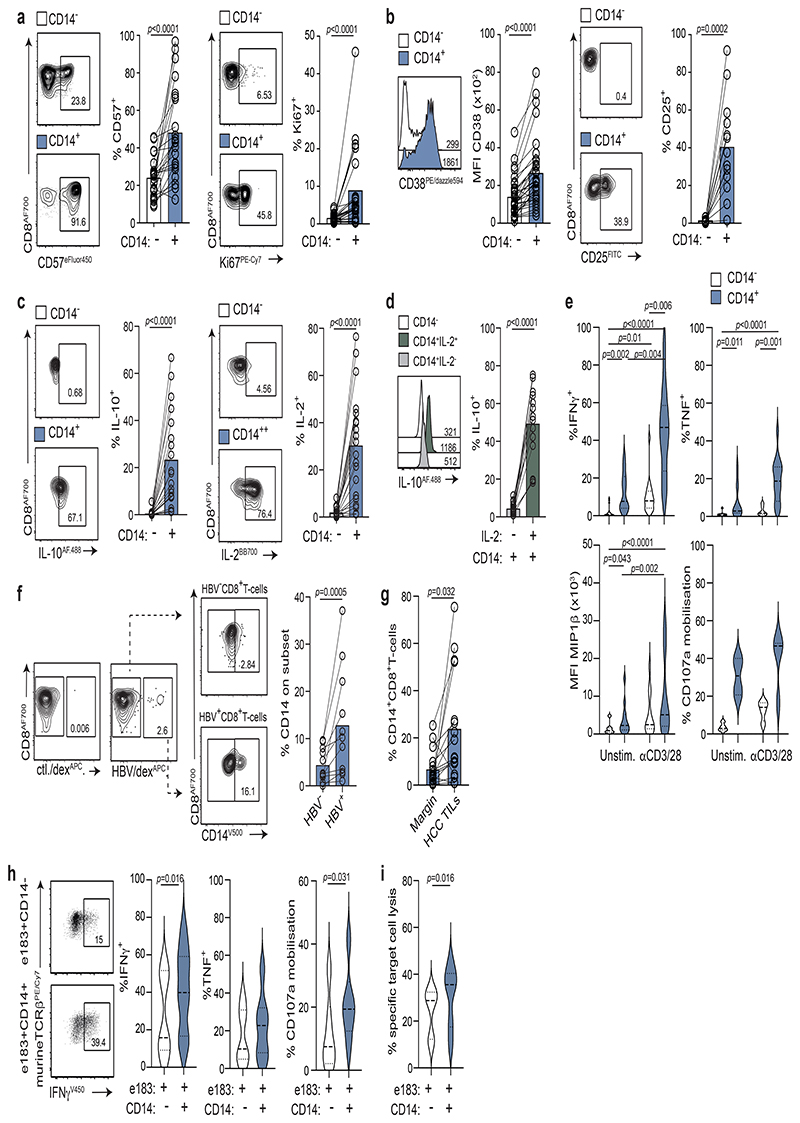
Immunomodulatory function of CD14^+^CD8^+^T-cells. Representative flow cytometric plots and summary data showing percentage or MFI of **a** CD57 (n=23), Ki67 (n=34) and **b** CD25 (n=13), CD38 (n=34) and PD-1 (n=36) on intrahepatic CD14^-^CD8^+^T-cells (black outline) or CD14^+^CD8^+^T-cells (blue filled) *ex vivo*. **c** Representative flow cytometric plots and summary data showing percentage intracellular staining for IL-10 (n=19) and IL-2 (n=21) by CD14^-^CD8^+^T-cells or CD14^+^CD8^+^T-cells *ex vivo* (without exogenous stimulation or brefeldin-A), and **d** co-production of IL-10 *ex vivo* by IL-2-positive or IL-2-negative CD14^+^CD8^+^T-cells (n=16). **e** Assessment of CD14^+^CD8^+^T-cell intracellular cytokine production ± 4hr anti-CD3/CD28 stimulation in the presence of brefeldin-A: IFNγ (n=19), TNF (n=15), MIP1β (n=12) and CD107a (n=5). **f** Intrahepatic HBV-specific CD8^+^T-cells identified using *ex vivo* staining with a panel of HLA-A2^+^ dextramers loaded with immunodominant peptides, gated using a control HLA-A2^+^ dextramer loaded with an irrelevant peptide (ctl./dex). Example plots and summary data depict CD14 expression on the global (HBV dex-negative) or HBV-specific CD8^+^T-cells (n=11). **g** Frequency of CD14^+^CD8^+^T-cells in HCC TILs in comparison to paired non-cancerous tissue (margin) from the same study participant (n=16). Functionality of FACS-purified TCR-redirected HBVenv183-specific CD14± CD8^+^T-cells against HepG2-preS1 presenting endogenously processed peptide at E:T ratio 1:2.5 for 16 hr by **h** ICS for IFNγ, TNF production (n=7) and CD107a mobilisation (n=6) and **i** specific target cell lysis (n=9). Each dot (or pair of dots) represent(s) a study participant or PBMC donor, processed, stained and analysed independently; bars represent mean. **e, h-i** solid lines: median, dotted lines: IQR. *p*-values determined using either a Kruskal-Wallis test (ANOVA) with Dunn’s multiple comparisons test **e**; or a two-tailed Wilcoxon *t* Test **a-d, f-i**.

We postulated that liver CD8^+^T_RM_ distinguished by the acquisition of CD14 would have distinct functional features instructed by myeloid cells. Hepatic myeloid cells can shape the tolerance and/or activation of neighbouring T-cells through local cytokine release or transpresentation, exchange of metabolites, antigen presentation and co-stimulation^16,19–23^. A gain-of-function screen recently identified several myeloid-restricted genes as the most potent at remodelling T-cells to promote their activation and effector function^24^. We therefore investigated whether the fraction of human liver CD8^+^T-cells staining for CD14 had a distinct activation status and functional potential compared to their CD14^-^CD8^+^T-cell counterparts directly *ex vivo*. A higher proportion of human liver CD14^+^CD8^+^T-cells expressed the chronic activation marker CD57 and the proliferation marker Ki67 than the remaining intrahepatic CD8^+^T-cells (Fig.3a), contrasting with prototypical CD8^+^T_RM_ which have low turnover^6,25^. *Ex vivo* human liver CD14^+^CD8^+^T-cells constituted a highly activated population, with significantly increased CD38 and a striking upregulation of CD25, but equivalent percentage of PD-1 expression, compared to CD14^-^CD8^+^T-cells (Fig.3b). In line with the increased nutrient uptake required to maintain a highly activated state, CD14^+^CD8^+^T-cells had a consistent increase in system-L amino acid transporters marked by CD98, and in the transferrin receptor CD71 (Extended Data Fig.3a). Other cardinal features of highly activated/immunomodulatory T-cells that segregated exclusively with the CD14^+^ fraction of CD8^+^T-cells were Foxp3, CTLA-4, and co-expression of the ectonucleotidases CD39 and CD73, with expression of LAG-3, GITR and ICOS also increased (Extended Data Fig.3b,c). Intrahepatic CD14^+^CD8^+^T-cells were potent constitutive producers of the immunoregulatory cytokine IL-10 directly *ex vivo* (without stimulation or brefeldin-A, mean 23%, max. 67% [Fig.3c left panel]). Cell-autonomous IL-10 has been shown to protect CD8^+^T-cells from excessive stimulation^26^, thereby promoting their function against chronic hepatotropic viral infections^27^. Similarly, abundant CD8^+^T-cell production of IL-2, that can counteract PD-1-mediated tolerance in the liver^6,28^, was seen in the CD14-expressing fraction without the need for *ex vivo* stimulation, frequently co-expressed with IL-10 (Fig.3c right panel,d). This combination of functional features characterised CD14^+^CD8^+^T-cells as a constitutively proliferative, activated, population with an immunomodulatory cytokine profile associated with maintenance of effector function in the liver.

Upon short-term (4 hr) TCR engagement, CD14^+^CD8^+^T-cells maintained their high constitutive production of IL-10 and IL-2 (Extended Data Fig.3d), whilst simultaneously further increasing their high steady-state production of the effectors IFNγ, TNF and MIP1β and their capacity for cytotoxic degranulation (CD107a), all of which are important anti-pathogen and anti-tumour functions (Fig.3e). This was not simply a generic feature of tissue residence, since the liver CD8^+^T_RM_ fraction expressing CD14 were more functional than the remainder of CD8^+^T_RM_ lacking CD14 (Extended Data Fig.3e). Thus, CD14 staining demarcates a population of human liver CD8^+^T_RM_ enriched for high constitutive production of immunoregulatory cytokines and the capacity to additionally produce proinflammatory effectors immediately following TCR engagement. The disease relevance of the heightened immunomodulatory potential of CD14^+^CD8^+^T-cells was underscored by the finding that hepatitis B virus (HBV)-specific responses (identified by HLA/peptide multimer staining of intrahepatic leukocytes (IHL) extracted from HBV-infected livers) were more than two-fold enriched for the expression of CD14 compared to the residual global CD8^+^T-cells (Fig.3f). CD14^+^CD8^+^T-cells were also ˜3-fold enriched amongst tumour-infiltrating leukocytes (TILs) freshly isolated from resected HCC compared to paired unaffected liver margins (Fig.3g), in line with this tumour type being rich in the stromal factors (ECM proteins and CXCL12^11^) that we had found promoted CD14 acquisition by CD8^+^T-cells.

We then examined whether CD8^+^T-cells interacting with MNP *in vitro*, marked by the acquisition of CD14, could recapitulate the enhanced functionality we observed in this fraction of liver CD8^+^T-cells when sampled directly *ex vivo*. CD8^+^T-cells acquiring CD14 following *in vitro* culture with pHSC and myeloid cells had enhanced effector function compared to the fraction not co-staining for CD14, with increased IL-2, IFNγ, TNF and CD107a by flow cytometry (Extended Data Fig.3f, top panel) and increased IL-2, IFNγ, TNF, IL-10, granzyme B by Luminex profiling of sorted anti-CD3/CD28-stimulated CD14^+^CD8^+^T-cells (Extended Data Fig.3f bottom panel). To test whether MNP instruction, marked by CD14 acquisition, had therapeutic potential for antigen-specific T cells, we used TCR-gene transduced T-cells specific for an HBV tumour antigen, that are being trialled for immunotherapy of HCC^29^. T-cells genetically engineered with a TCR specific for HBVenv183/HLA-A2 were expanded in the presence of MNP and stellate cells. The CD14 expressing fraction of TCR-transduced CD8^+^T-cells induced by this *in vitro* protocol (representative example Extended Data Fig.3g) had the hyper-functionality upon TCR engagement we had previously observed *ex vivo*; significantly more of the CD14^+^TCR-gene transduced CD8^+^T-cells produced the anti-viral/anti-tumour effectors IFNγ, TNF and degranulated within 16 hr of stimulation with an HBsAg-expressing hepatoma cell line compared to their CD14-negative counterparts (Fig.3h). Moreover, specific lysis of the HBsAg-expressing hepatoma cell line by HBVenv183/HLA-A2 TCR-redirected T-cells was enhanced by those that had acquired CD14 following addition of MNP (Fig.3i). These data suggested that CD14 expression marks a fraction of “super-responder” CD8^+^T-cells that have undergone MNP re-programming, both *in situ* in human liver and *in vitro* in TCR gene-engineered populations.

## Bacterial LPS shapes CD14^+^CD8^+^T-cells

**Extended Data Figure 4:**
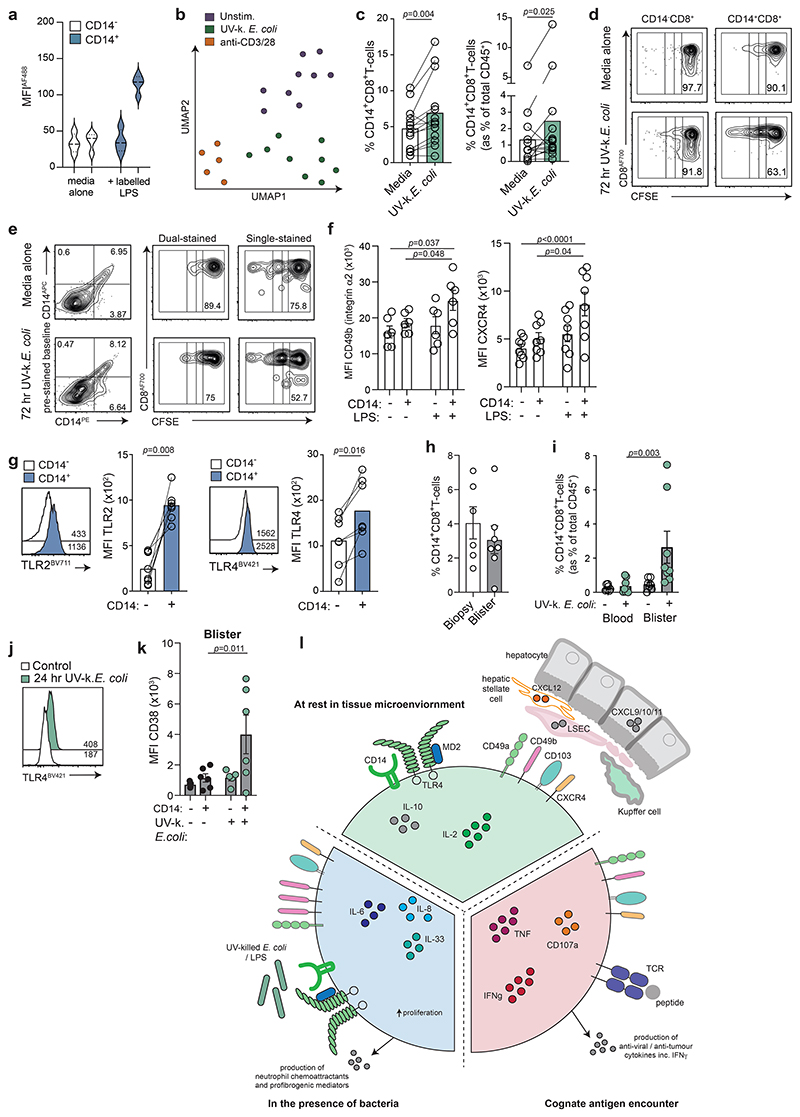
LPS-induced effects on CD14^+^CD8^+^T-cells *in vitro*. **a** MFI of bound LPS^AlexaFluor488^ on stromal cell and MNP-induced CD14^-^CD8^+^T-cells or CD14^+^CD8^+^T-cells (n=5) after culture for 120min with LPS^AlexaFluor488^, or media alone. **b** UMAP: soluble mediator profiles produced by FACS-sorted stromal cell-induced CD14^+^CD8^+^T-cells ± stimulation with media alone (purple), 0.3×10^6^ UV-killed *E. coli* (green) or anti-CD3/28 (orange). **c** Percentage CD14 on intrahepatic CD8^+^T-cells ± 72hr exposure to 0.3×10^6^ UV-killed *E. coli* as a proportion of total CD8^+^T-cells (n=14) or a proportion of total intrahepatic leukocytes (CD45^+^; n=13). **d** CFSE dilution of intrahepatic CD14-labelled CD14^+^CD8^+^T-cells after 72hr culture *in vitro* in the presence of UV-killed *E. coli* and **e** the co-expression of intrahepatic CD14-labelled CD14^+^CD8^+^T-cells (APC; prelabelled on d 0) and further stained with CD14^PE^ 72 hr after *in vitro* culture in the presence of 0.3x10^6^ UV-killed *E. coli*. **f** MFI expression of CD49b (n=6) and CXCR4 (n=8) on peripheral CD14 ± CD8^+^T-cells after co-culture in the presence of LPS. **g** Representative plots and summary data: MFI of TLR2 and TLR4 (n=7) on ascitic CD14^-^CD8^+^T-cells (black outline) or CD14^+^CD8^+^T-cells (blue filled) *ex vivo*. **h** Frequency of CD14^+^CD8^+^T-cells isolated from human skin punch biopsies (n=6; taken from the forearm of healthy controls) or from skin blister aspirates (n=7; control blister). **i** CD14^+^CD8^+^T-cells as a proportion of total leukocytes (CD45^+^) from blood (n=8) or blister exudates (n=8) with or without prior UV-killed *E. coli* intradermal injection. **j** TLR4 expression on CD8^+^T-cells aspirated from skin blisters ± UV-killed *E. coli* injection (representative of n=3 blister exudates). **k** Expression of CD38 on CD14^-^CD8^+^T-cells or CD14^+^CD8^+^T-cells from blister exudates ± UV-killed *E. coli* intradermal injection (n=6). **l** Overview schematic of the functional role of CD14^+^CD8^+^T-cells. Each dot (or pair of dots) represent(s) a study participant, processed, stained and analysed independently; bars represent mean, error bars (where appropriate) represent ± S.E.M. **a** solid lines: median, dotted lines: IQR. *p*-values were determined using a Mann-Whitney *U* Test **h**; two-tailed Wilcoxon *t* Test **c, g**; or a two-tailed Kruskal-Wallis test (ANOVA) with Dunn’s multiple comparisons test **f, i, k**

**Figure 4:**
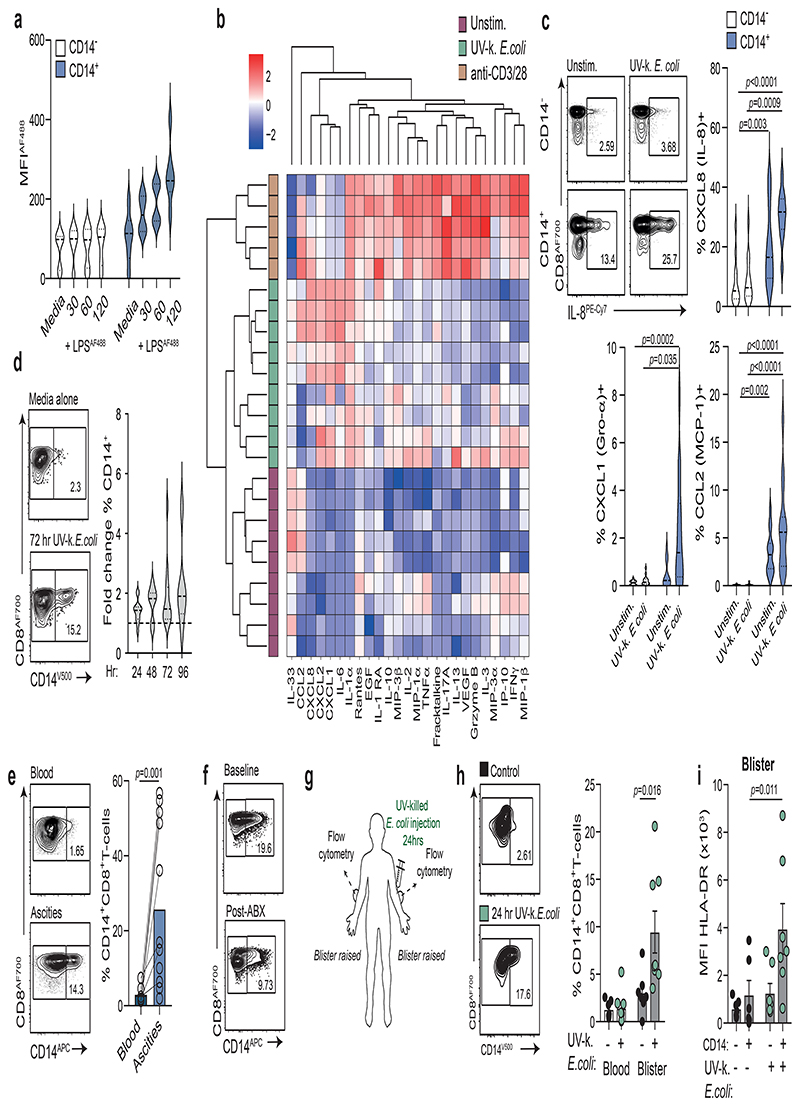
Bacterial LPS shapes CD14^+^CD8^+^T-cells. **a** MFI of LPS^AlexaFluor488^ bound to CD14^-^CD8^+^T-cells or CD14^+^CD8^+^T-cells (n=5) after addition for 30, 60 or 120min compared to media alone for 120min. **b** Heatmap showing expression profiles of soluble mediators produced by FACS-sorted pHSC-induced CD14^+^CD8^+^T-cells after overnight stimulation with anti-CD3/CD28 (orange), 0.3x10^6^ UV-killed *E. coli* (green) or unstimulated (purple) using unbiased hierarchical clustering. Left-hand dendrogram: sample similarity and top dendrogram: mediator similarity based on average-linkage using Euclidian distance. **c** pHSC and MNP-induced CD14^-^CD8^+^T-cell and CD14^+^CD8^+^T-cell functionality by ICS for CXCL8 (n=12), CXCL1 (n=15) or CCL2 (n=15) ± 16 hr UV-killed *E. coli* in the presence of brefeldin-A. **d** Percentage increase CD14 on intrahepatic CD8^+^T-cells after 24 (n=13), 48 (n=7), 72 (n=12) or 96hr (n=6) *in vitro* culture with 0.3×10^6^ UV-killed *E. coli* compared to media alone. **e** Representative plots and summary data: frequency of CD14^+^CD8^+^T-cells isolated from paired peripheral blood and ascitic fluid (n=11). **f** Frequency of CD14^+^CD8^+^T-cells in the ascitic fluid of a cirrhotic study participant with spontaneous bacterial peritonitis (SBP) pre-antibiotics (baseline) and post-antibiotics (post-ABX). **g** Schematic: human forearm skin blister model for *in vivo* UV-killed *E. coli* exposure. **h** Frequency of CD14^+^CD8^+^T-cells in blood or skin blister aspirate (grey filled) with (green dots) or without (black dots) intradermal injection of 1.5×10^7^ UV-killed *E. coli* 24hr previously (n=8). **i** Expression of HLA-DR on CD14^-^CD8^+^T-cells or CD14^+^CD8^+^T-cells from blister exudates with or without prior UV-killed *E. coli* intradermal injection (n=7). Each dot (or pair of dots) represent(s) a study participant/PBMC donor, processed, stained and analysed independently; bars represent mean and error bars (where appropriate) represent ± S.E.M.. **a,c**-**d** solid lines: median, dotted lines: IQR. *p*-values determined using either a Kruskal-Wallis test (ANOVA) with Dunn’s multiple comparisons test **c, h** and **i**; Wilcoxon *t* Test **e**.

We postulated that the heavy exposure to bacterial wall LPS and shed LPS in the liver may promote and/or differentially skew MNP interactions with locally resident T-cells to increase CD14^+^CD8^+^T-cells and/or alter their functional profile. Previous reports have shown that binding of exogenous CD14 can confer LPS responsiveness on cells lacking endogenous expression^30^ as well as suggesting that human CD8^+^T-cells can upregulate sufficient functional TLR4 to respond directly to LPS following anti-CD3 activation *in vitro*^31^. Firstly, we showed that fluorescently-labelled LPS preferentially bound to the CD14-expressing fraction of *ex vivo* liver CD8^+^T-cells and to stromal-cell induced CD14^+^CD8^+^T-cells, with a stepwise increase in binding over time (Fig.4a, Extended Data Fig.4a). Stimulation of FACS-sorted CD14^+^CD8^+^T-cells with UV-killed *Escherichia coli* (*E. coli*, as a source of LPS and other bacterial PAMPs sensed by CD14^32,33^) produced a distinct profile of soluble mediators compared to that seen at rest or after TCR engagement (Fig.4b, Extended Data Fig.4b). The CD14^+^CD8^+^T-cell response to *E. coli* was dominated by the production of innate-like hepatoprotective cytokines (IL-6, IL-33)^34,35^ as well as chemokines driving neutrophil recruitment, neutrophil recruitment of T-cells^36^ and wound healing, including CXCL8 (IL-8) (Fig.4b), previously noted to be produced by a fraction of HBV-specific CD8^+^T-cells^37^. These data raised the possibility that CD14^+^CD8^+^T-cells acquire some direct responsiveness to LPS following CD14 acquisition as described for other cell types previously^30^. *In vivo*, the dominant response of CD14^+^CD8^+^T-cells to LPS is likely to also be influenced by activation of interacting MNP. In the presence of MNP, the addition of UV-killed *E. coli* again induced CD14^+^CD8^+^T-cell production of CXCL8, CXCL1 (Gro-α) and CCL2, which were already increased in CD14^+^ compared to CD14^-^CD8^+^T-cells (Fig.4c). Thus CD14^+^CD8^+^T-cells, with or without addition of MNP and using two different readouts, had a propensity to produce pro-fibrogenic/wound-healing chemokines able to drive an influx of innate responders like neutrophils, and this property was further enhanced by re-exposure to bacterial LPS.

Finally we asked whether exposure to bacterial LPS could activate MNP and promote membrane transfer to T-cells to partially account for the increased frequencies of CD14^+^CD8^+^T-cells in tissues like the liver. Exposure of IHL to UV-killed *E. coli* augmented CD8^+^T-cells staining for CD14 over time (Fig.4d, Extended Data Fig.4c). In the presence of UV-killed *E. coli*, proliferating CD14^+^CD8^+^T-cells preferentially acquired new CD14 expression (distinguished by staining with a different CD14 antibody before and after CFSE division during culture) (Extended Data Fig.4d,e). In addition, LPS preferentially enhanced CD49b and CXCR4 expression on CD14^+^CD8^+^T-cells (Extended Data Fig.4f), thereby rendering them more sensitive to stromal signals.

The peritoneal cavity is another body site rich in ECM and CXCL12-producing mesothelial cells^38,39^ that is heavily exposed to gram-negative bacteria under the surveillance of liver capsular macrophages^40^. Consistent with these features, CD14^+^CD8^+^T-cells (co-expressing TLR4 and TLR2; Extended Data Fig.4g) were markedly increased in the ascites of patients with cirrhosis or non-cirrhotic portal hypertension compared to their blood, accounting for up to 57% (mean 25%) of all ascitic CD8^+^T-cells (Fig.4e). Of note, the proportion of CD14^+^CD8^+^T-cells within the ascitic fluid of a cirrhotic patient with the common complication of spontaneous bacterial peritonitis halved on repeat sampling following a course of antibiotics (Fig.4f), suggesting that reduced LPS activation of MNP was reflected in decreased CD8^+^T-cells acquisition of CD14.

To more definitively test the postulate that LPS activation could drive CD8^+^T-cell acquisition of CD14, we turned to a human challenge model. We examined the impact of *E. coli in vivo* using a human skin suction blister challenge model^41^, in which we found CD14^+^CD8^+^T-cells detectable at a similar frequency to skin (Extended Data Fig.4h). Seven healthy volunteers had suction blisters raised on both forearms, following intradermal injection of UV-killed *E. coli* 24 hr previously in one arm but not the contralateral arm (Fig.4g). CD14^+^CD8^+^T-cells increased within the cellular exudate aspirated from the blister raised 24 hr-post injection with UV-killed *E. coli* compared to the control blister, and remained barely detectable in paired blood samples, indicating a localised *in vivo* response (Fig.4h, Extended Data Fig.4i). The bacterial-induced increase in CD8^+^T-cell CD14 was accompanied by an increase in HLA-DR, TLR4 and CD38 (Fig. 4i; Extended Data Fig.4j,k). Thus *in vivo* tissue-localised introduction of *E. coli* was able to induce the acquisition of MNP-derived molecules by CD8^+^T-cells, thereby re-shaping local T-cell immunity at the site of bacterial exposure.

## Discussion

Memory T-cells undergo extensive re-programming upon acquiring organ residence but the local tissue components that can drive these distinct features are poorly understood. Examining CD8^+^T-cells directly isolated from human liver tissue, we find that those distinguished by co-staining for CD14, and other prototypic myeloid markers, have increased turnover, stromal homing potential and immunomodulatory effector function. CD14^+^CD8^+^T-cells have high constitutive production of the immuno-protective cytokine IL-10, mount rapid and potent effector function upon TCR engagement, and switch to chemotactic/hepatoprotective function upon LPS exposure (schema, Extended Data Fig. 4l). This profile can be recapitulated by CD8^+^T-cell ICAM-1/actin-dependent acquisition of membrane proteins including the LPS receptor from MNP, consistent with the closer proximity of CD14^+^CD8^+^T-cells to MNP *in situ* in human liver. Our data therefore suggest that MNP instruction drives the increased turnover and hyper-functionality of tissue CD8^+^T_RM_ marked by CD14; this concept is supported by a recent study showing highly efficient remodelling of T-cell proliferation and effector function by over-expression of membrane receptors such as LTBR, usually restricted to myeloid cells^24^. MNP can also re-shape T-cell functionality through cell-cell transfer of specific metabolites^19^. Furthermore, a subset of Kupffer cells (KC2) has recently been defined in mouse and human liver that can be activated by IL-2 to cross-present antigen to convert tolerised into functional CD8^+^T-cells; of note the latter are distributed more widely within the liver than the periportal-localised CD8^+^T-cells primed by hepatocytes, reminiscent of the zonal differences we observed for CD14^+^ versus CD14^-^CD8^+^T-cells^16,17^. Thus further mechanistic studies are needed to investigate whether MNP-derived cytokines, receptors like lymphotoxin β receptor (LTBR), metabolites and/or antigen presentation contribute to the distinct topology and functional profile of hepatic CD8^+^T-cells marked by CD14. The accumulation of CD14^+^CD8^+^T-cells in zone 2 of the liver suggests that interactions with CD14^hi^ myeloid cells are favoured in this midlobular region. It remains to be clarified what downstream signalling changes occur in tissue CD8 T-cells that have been re-programmed by myeloid cells to account for their altered constitutive, TCR- and LPS-dependent functions.

Tissue stroma and chemokines in the ECM are increasingly recognised to play a fundamental role in dictating organ-specific immune responses^42–45^. Consistent with this, we find that CD14^+^CD8^+^T-cells express receptors for ECM and CXCL12, stromal-derived factors that we demonstrate can promote CD8^+^T-cell acquisition of CD14. Hepatic stromal (stellate) cells are known to play a pivotal role in imprinting Kupffer cell identity on MNP^46^ and coordinating their response to LPS^47^; we now demonstrate stellate cells also collaborate with myeloid cells to render liver CD8^+^T_RM_ responsive to LPS. LPS, and stromal factors like CXCL12, are likely to predominantly expand CD14^+^CD8^+^T-cells through increased MNP activation promoting transfer of the LPS receptor complex to T-cells. We show that inactivated *E. coli* further increase the capacity of CD8^+^T-cells for the stromal interactions that we found support their CD14 acquisition, congruent with a previous report of LPS driving T-cell adherence to fibronectin^48^. Our data reveal that *E.coli* exposure increases CD14^+^CD8^+^T-cell frequencies *in vitro* and *in vivo* in a skin blister model, likely contributing to their preferential accumulation in sites rich in bacterial LPS like the liver and cirrhotic ascites. The LPS-induced myeloid collaboration with CD8^+^T-cells provides a means by which bacterial products can mould a highly immunomodulatory fraction of hepatic CD8^+^T_RM_, including virus-specific responses. The recognition that LPS can also skew CD14^+^CD8^+^T-cells towards rapid production of chemotactic cytokines points to an unappreciated early role for tissue T-cells in inflammation and hepatoprotective responses triggered by gram-negative bacteria or LPS that breach the intial firewall formed by periportal MNP to reach the midlobular zone responsible for hepatocyte homeostasis^18,45^.

Our data imply that antibiotics or other drugs lowering the amount of gut bacterial LPS in the portal circulation will reduce CD14^+^CD8^+^T-cells, whilst TLR4-based adjuvants such as monophosphoryl-lipid A or stromal factors like CXCL12 could be targeted to the liver to promote myeloid-reprogramming. The recognition that MNP can re-programme T-cell effector function can be exploited therapeutically; for example, we show that myeloid co-cultures can be used to generate TCR gene-edited T-cells with superior immunotherapeutic potential for adoptive cell therapy. Further studies are needed to explore the immunomodulatory potential of CD14^+^CD8^+^T-cells preferentially accumulating amongst the long-lived donor T-cells surviving in HLA-mismatched allografts and within HCC TILs and whether they become tolerant to LPS, as described for liver MNP upon repetitive high-dose exposure^49^. Of note, studies of human CD8^+^T_RM_ typically gate out CD14-expressing cells, precluding previous analysis of this population. The finding that CD14^+^CD8^+^T-cells also accumulate in cirrhotic ascites suggests they may play a role in monitoring bacterial invasion of the peritoneal cavity and underscores the need to study them in other human barrier organs heavily exposed to LPS. Our study exemplifies the importance of sampling and monitoring human T_RM_ to uncover the tissue-imposed adaptations that allow them to respond to local microenvironmental cues.

## Methods

### Study samples, ethics and inclusion

Each study participant provided written informed consent prior to inclusion, with the storage of any samples/clinical information conforming to the requirements of the Declaration of Helsinki, the Data Protection Act 1998 and the Human Tissue Act 2004.

Liver samples were either obtained from non-diseased distal margins of colorectal metastases or margins of hepatocellular carcinoma surgical resections (either on the background of chronic hepatitis B/hepatitis C infection, non-alcoholic steatohepatitis or alcoholic liver disease), explants obtained upon solid-organ transplantation, or perfusion fluid (perfusates) obtained from the donor livers used for solid-organ transplantation. Spleen and lymph nodes samples were obtained from the NHSBT organ donation pathway. The majority of tissue and blood samples were obtained through the Tissue Access for Patient Benefit Initiative (TAPb) at The Royal Free Hospital, Hampstead, London (approved by the UCL-Royal Free Hospital BioBank Ethical Review Committee references: 11/WA/0077, 16/WA/0289 and 21/WA/0388); healthy donor blood samples were covered by REC 11//LO/0421 (approved by South East Cost Brighton and Sussex Ethics Committee). Further liver tissue samples were obtained in Freiburg for imaging mass cytometry (approved by the ethics committee of the Albert-Ludwigs-University; Freiburg reference: #21-1372), and from The Royal London Hospital (approved by the London Brent & The City Research Ethics Committee references: 16/LO/1699 or the London Bridge Research Ethics Committee reference 17/LO/0266). Skin experiments were taken under UCL Research Ethics Committee project numbers 87878 for biopsies and 1309/005 for skin blister/E.coli challenge studies. Ascitic fluid was obtained from study participants with cirrhosis or IVC obstruction requiring an ascitic tap at either the Royal Free Hospital, London (approved by the UCL Research Ethics Committee reference: 15/LO/0800), the Hospital Clínic de Barcelona (approved by Ethics Committee Hopsital Clínic de Barcelona, Committee reference: 2019-0267) or the Royal London Hospital (approved by the London Bridge Research Ethics Committee reference 17/LO/0266), assessed for spontaneous bacterial peritonitis (SBP) and in one case treated with Norfloxacin before re-sampling. HIV seropositive donors were excluded from the study but there were no other exclusions; all available samples with sufficient viable cell yields for the experimental protocol were included.

### Human cell isolation

Human intrahepatic leukocytes (IHL) were isolated from resected/explanted liver material as previously described^50^. Briefly, tissue was cut up and incubated at 37 °C for 30 min in HBSS^+/+^ (Life Technologies; ThermoFisher Scientific) containing collagenase IV (ThermoFisher Scientific) and DNaseI (Roche; Sigma Aldrich; Merck), prior to mechanical disruption using a GentleMACS (Miltenyi Biotech) with debris removed by filtration through a 70 µM filter (Greiner). Parenchymal cells were removed by centrifugation on a 30% Percoll gradient (GE Healthcare; VWR) followed by a further leukocyte isolation by density centrifugation using a Pancoll gradient (PAN Biotech). IHL were isolated from perfusion liquid (perfusates) or small core-biopsy samples as previously described^51^. Briefly, the perfusate fluid was first concentrated by centrifugation. Concentrated cells were then resuspended in RPMI-1640 (Life Technologies; ThermoFisher Scientific) and isolated by density centrifugation on a Pancoll gradient.

For the isolation of human leukocytes from the skin, spleen and lymph nodes, tissue was first cut into small pieces and washed with an EDTA-containing buffer (2mM EDTA [Sigma Aldrich; Merck] + 0.5% heat inactivated fetal bovine serum [HI-FBS; Sigma Aldrich; Merck] in 1x phosphate buffered saline [PBS; Life Technologies; ThermoFisher Scientific]). For the skin an additional enzymatic digestion (using the Whole Skin Digestion kit; Miltenyi Biotech) was required overnight. Mechanical disruption was performed using the barrel of a 5 mL sterile syringe to pass the cells through a 70 µM filter. Concentrated cells were resuspended, and leukocytes isolated by density centrifugation on a Pancoll gradient. Leukocytes obtained from the spleen and lymph nodes were further subjected to a red blood cell lysis step (BioLegend), on ice, prior to use. All intrahepatic, skin, splenic and lymphoid cell samples were used immediately.

For the isolation of human leukocytes from ascitic fluid, the fluid drained from the patient was concentrated by centrifugation and washed twice with RPMI-1640. Where necessary leukocytes were further subjected to a red blood cell lysis prior to use and frozen in 10% DMSO (Sigma Aldrich; Merck)-HI FBS if not used immediately.

Where possible peripheral blood was obtained from the same study participants for comparison. Peripheral blood mononuclear cells (PBMC) were isolated from heparinised peripheral blood by density centrifugation using a Pancoll gradient.

### Multiparametric flow cytometry

Multi-parametric flow cytometry was used for phenotypic and functional analysis. For analysis of the CD14-expressing T-cells a strict gating criterion was used throughout. Cells were stained with a Blue fixable Live/Dead dye (Invitrogen; ThermoFisher Scientific) for the exclusion of dead cells. Doublets, CD45-, CD56^+^, CD19^+^, CD3^+^CD4^+^ cells were also excluded from the analysis (sequential gating strategy: Extended Data Fig.1a). In functional and sorting experiments, and for confirmatory phenotyping, additional gating excluded CD3^+^Vα7.2^+^ MAIT-cells and γδ-T cells (gating strategy, Extended data Fig.2f). Surface markers were stained with saturating concentrations of monoclonal antibodies (mAbs) for 30 min at 4 °C in 50% diluted brilliant violet buffer (BD Bioscience). To avoid non-specific antibody binding to Fc receptors in *ex vivo* stains of tissue lymphocytes, an additional Fc receptor blocking step (Miltenyi Biotech; catalogue number: 130-059-901) was included as per manufacturers instructions prior to surface antibody labelling. In the majority of experiments, CD14 was detected with a Miltenyi Biotech REAfinity Recombinant Antibody (IgG1 Fc region mutated to abolish binding to Fcγ receptors). Cells were fixed using Cytofix/Cytoperm (BD Bioscience) or the Foxp3 buffer kit (BD Bioscience). Intracellular/intranuclear markers were subsequently stained in a 0.1% saponin-based buffer (Sigma Aldrich; Merck) or 1x PBS for 30 min at 4 °C. Full details of mAbs, including dilutions/catalogue numbers, are detailed in Supplementary Table 1. All samples were acquired in 1x PBS on a BD Bioscience LSR-FortessaX20-SORP running DIVA v. 8.0.1 and analysed using FlowJo v. 9.9.4 or v. 10.8.1 (TreeStar; BD Bioscience).

### Imaging flow cytometry

For confirmation of single cell expression of αβTCR, CD3, CD8, CD14 and TLR4 by imaging cytometry global CD3^+^T-cells were first isolated using the magnetic bead-based human Pan T-cell negative selection kit (MACS-bead isolated; Miltenyi Biotech) from three liver samples; and stained as described in *Multiparametric flow cytometry* with the addition of a DAPI nuclear dye (Sigma Aldrich; Merck). For confirmation of *in vitro* membrane acquisition by CD8^+^T-cells, MACS-bead isolated CD8^+^T-cells (isolated as per the manufacturer’s instruction using the human CD8^+^T-cell isolation kit (Miltenyi Biotech) were assessed for expression of αβTCR, CD3, CD8, CD14, and further stained with a PE-labelled streptavidin antibody to detect the biotin acquired directly from mononuclear phagocytes (MNP) or monocyte-derived macrophages (MDM) as described in *Isolation of mononuclear phagocytes (MNP) and the in vitro generation of monocyte-derived macrophages (MDM)*. Samples were acquired in 1x PBS on an ImageStreamX MKII (Merck) running INSPIRE v. 200.1.388.0 with a 60X optic and analysed using Amnis Ideas. Doublets were discriminated by gating area vs. aspect ratio by detecting high aspect singlets, with a histogram gradient RMA to define in focus single cells.

### CD14^+^CD8^+^T-cell cytospin

Intrahepatic leukocytes were sorted by FACS for CD14± CD8^+^T-cells as follows: Singlets, CD3^+^CD56^-^CD19^-^CD4^-^CD8^+^Vα7.2^-^ and split in to CD14± based on an isotype control. Cells were isolated into chilled FACS-tubes coated with FBS and prefilled with 500 μL sterile 1x PBS for cytospin. Giemsa staining (Sigma-Aldrich; Merck) was used to determine morphology of sorted cells on cytospins. Slides were viewed using a Zeiss AxioImager microscope, images taken of four fields from n=2 samples using the 100x objective, and viewed using Zen (v. 2.3).

### Detection of CD14-expressing CD8^+^T-cells in the setting of HLA-mismatch transplantation

An additional cohort of five study participants undergoing re-transplantation (i.e. receiving a second liver transplant) for disease recurrence (not chronic rejection; obtained through the *TAPb Initiative*, as above). Detection of donor and recipient leukocytes from isolated IHL were determined on the basis of an human leukocyte antigen (HLA) class-I mismatch by flow cytometry. HLA-haplotyping was further confirmed by PCR, undertaken by Antony Nolan (NHS, London, UK) or MRC Weatherall Institute of Molecular Medicine Sequencing Facility (Oxford, UK). Samples were further profiled as described in *Multiparametric flow cytometry* to determine CD14 expression.

### Isolation of primary human hepatic stellate cells and co-cultures with PBMC/CD8^+^T-cells

To generate CD14-expressing CD8^+^T-cells *in vitro* freshly isolated PBMC from healthy control donors were co-cultured in complete RPMI-1640 (cRPMI; containing 10% FBS, 100 IU/mL penicillin/streptomycin, 20 mM HEPES, 0.5 mM sodium pyruvate, MEM non-essential amino acids, MEM essential amino acids and 50 µM β–mercaptoethanol; all Life Technologies; ThermoFisher Scientific) for 4 d in the presence of 40 IU/mL recombinant human IL-2 (PeproTech) at 37 °C with primary human hepatic stellate cells from three donors. Hepatic stellate cells were isolated as previously described^51,52^ from liver tissue samples using density centrifugation with an Optiprep (Sigma Aldrich; Merck) gradient. Pre-isolated hepatic stellate cells were thawed and cultured in 25/75 cm^2^ tissue culture flasks in stellate cell media (ScienCell Research Laboratories) to approximately 90% confluency prior to co-culture. For the co-culture experiments hepatic stellate cells were detached using trypsin-EDTA (Life Technologies; ThermoFisher Scientific) and re-seeded at a density of 0.15 × 10^5^ cells/well in cRPMI and left to adhere prior to the addition of 0.5 × 10^6^ freshly isolated PBMC. After 4 d co-culture PBMC were harvested and profiled as described in *Multiparametric flow cytometry* for CD14 expression and phenotype. Where indicated T-cells were purified from freshly isolated PBMC prior to hepatic stellate cell co-culture using the pan T-cell isolation kit or the CD8^+^T-cell isolation kit for comparison with bulk PBMC. In certain indicated T-cell/hepatic stellate cell co-culture experiments the cells were further supplemented with 5 µg/mL anti-CXCR4 (BioTechne) or IgG isotype control (BioTechne).

### CXCL12 ELISA

Cell-free supernatants from primary human hepatic stellate cells from three donors cultured in 24-well flat bottom plates for 4 d in stellate cell media at various cell densities were harvested as assessed for CXCL12 production using the Human CXCL12/SDF-1α Quantikine ELISA kit (BioTechne) according to the manufacturer’s instructions.

### In vitro derivation of CD14-expressing CD8^+^T-cells with cytokines

To generate CD14-expressing CD8^+^T-cells *in vitro* freshly isolated PBMC from healthy control donors were co-cultured in cRPMI for 4 d in the presence of 40 IU/mL recombinant human IL-2 (PeproTech) at 37 °C with the addition of either 50 ng/mL recombinant human: IL-6 (R&D Systems), IL-8 (R&D Systems), IL-15 (PeproTech), TGFβ (R&D Systems), CXCL9 (BioLegend), CXCL10 (R&D Systems), CXCL11 (BioLegend), CXCL12 (BioTechne) or CXCL16 (R&D Systems). After 4 d co-culture PBMC were harvested and profiled as described in *Multiparametric flow cytometry* for CD14 expression and phenotype.

### Isolation of mononuclear phagocytes (MNP) and the in vitro generation of monocyte-derived macrophages (MDM)

For the isolation of autologous mononuclear phagocytes (MNP), PBMC were freshly isolated from heparinised blood and subjected to the pan monocyte isolation kit (Miltenyi Biotech) as per the manufacturer’s instructions.

To generate monocyte derived macrophages (MDM), 5 x 10^6^ PBMC/mL were plated in 24-well plates for 2 hr in RPMI-1640 supplemented with 10% HI-FBS to allow for MNP adherence prior to being washed thoroughly in 1x PBS to remove non-adherent cells. Post initial adherence step MNP were exposed to macrophage differentiation media (RPMP-1640 supplemented with 10% human AB-serum and 20 ng/mL recombinant human MSCF [BioLegend]) for 6 – 10 d, with media changes every ˜3 d.

### Biotin-labelling of MNP/MDM

Where indicated, purified MNP or *in vitro* derived MDM were labelled with EZ-Link Sulfo NHS-LC-LC-Biotin (ThermoFischer Scientific) as previously described^53^. Briefly MNP/MDM were incubated with 1x PBS containing 10 µg/mL bitotinylation reagent for 10 min at 37 ºC. To quench, an equal volume of HI-FBS was added per well prior to the MNP/MDM being washed thoroughly and used in co-culture experiments with autologous CD8^+^T-cells. Autologous CD8^+^T-cells were co-cultured with labelled MNP/MDM in cRPMI for 4 d in the presence of 40 IU/mL recombinant human IL-2 at 37 °C at an approximate 1:1 ratio. After 4 d co-culture CD8^+^T-cells were harvested and profiled as described in *Multiparametric flow cytometry* for CD14 expression and phenotype.

### CD14 acquisition by peripheral CD8^+^T-cells

To assess the ability of CD8^+^T-cells to acquire CD14 from MNP a series of *in vitro* trogocytosis assays were performed using freshly isolated peripheral CD8^+^T-cells. Autologous magnetic-bead isolated CD8^+^T-cells and magnetic-bead isolated MNP were co-cultured with primary human hepatic stellate cells in cRPMI supplemented with 100 IU/mL IL-2 in the presence or absence of the following inhibitors/antibodies/reagents: 25 µM Latrunculin B (actin polymerisation inhibitor), 100 nM Nocodazole (microtubule polymerisation inhibitor), 1 µg/ml anti-ICAM or the matched isotype control or 5 µg/ml soluble CD14 (BioLegend). In other experiments where indicated the MNP and CD8^+^T-cells were physically separated with 0.4 µM transwell inserts or with prior fixation of the MNP fraction for 10 mins with 4% paraformaldehyde. After fixation MNP were washed thoroughly in 1x PBS to remove any residual fixative. After 4 d co-culture CD8^+^T-cells were harvested and profiled as described in *Multiparametric flow cytometry* for CD14 expression and phenotype.

### Exosome purification and analysis from MDM supernatants and re-addition to CD8^+^T-cells

To exclude a role for T-cell acquisition of CD14 by exosome-mediated transfer, exosomes were isolated and purified from MDM from three donors (generated as described in *Isolation of mononuclear phagocytes [MNP] and the in vitro generation of monocyte-derived macrophages [MDM]*) using the CD63 exosome isolation kit (Miltenyi Biotech) as per the manufacturer’s instructions. Briefly, cellular debris was removed from the cell-free MDM supernatants by centrifugation, prior to the 1 hr incubation with the kit-provided exosome isolation microbeads. Post incubation exosomes were purified from the supernatant by magnetic bead isolation and quantified using the Bicinchoninic Acid (BCA) assay (ThermoFisher Scientific). The MACSPlex Exosome Detection kit (Miltenyi Biotech) was used as per the manufacturer’s instructions to phenotype and analyse the MDM-isolated exosomes by flow cytometry after high-speed centrifugation.

Once purified, 5 µg/mL primary human MDM-derived exosomes were co-cultured with isolated autologous CD8^+^T-cells in cRPMI supplemented with 40 IU/mL IL-2. After 4 d co-culture CD8^+^T-cells were harvested and profiled as described in *Multiparametric flow cytometry* for CD14 expression and phenotype.

### Confocal imaging of in vitro derived CD14^+^CD8^+^T-cells

Autologous MDM/CD8+T-cell co-cultures were carried out in 24-well plates containing poly-Lysine coated coverslips (refer to *Isolation of mononuclear phagocytes (MNP) and the in vitro generation of monocyte-derived macrophages [MDM]*). After co-culture coverslips were removed and mounted on slides for image acquisition. Prior to staining slides were subjected to a 1 hr blocking step using 10% goat serum/1%BSA/0.001 Triton-TX100 in 1x PBS at R.T. After blocking, slides were incubated with unconjugated antibodies against rat anti-human CD8 and rabbit anti-human CD14 (ThermoFisher) for a further 18 hr and washed thoroughly in 1X PBS. Primary antibody detection required further staining with secondary fluorochrome labelled antibodies: goat anti-rat AlexaFluor-488, and anti-human Alexa647 conjugates (Jackson Immuno Research) for 1 hr. All slides were also labelled with Hoechst33342 (Thermo Fisher).

Images were acquired using a Zeiss LSM880 Airyscan confocal microscope at 63X, 4X zoom (PLAN-Apochromat 63x/1.4NA) and 20X (EC Plan-Neofluar 0.5NA) magnification. For representative images, 63x/1.4NA Z-stacks were acquired and visualised as maximum intensity projections. For quantification of CD14, confocal 20X magnification Z-slices were acquired in the medial plane of the CD8 signal in the CD8^+^T-cell alone and MDM-associated CD8^+^T-cell populations. Image analysis was carried out in FIJI ImageJ with 30 CD8^+^T-cells quantified per condition (Schindelin et al., 2012). Nuclear segmentation occurred via segmentation of Hoechst33342 fluorescence. T-cell populations were identified, and their cell boundaries segmented using the extent of CD8 expression. Mean fluorescence of CD14 was then quantified within the segmented CD8^+^T-cell populations.

### Mass cytometry imaging of CD14^+^CD8^+^T-cells in human liver

Imaging Mass Cytometry was conducted as reported previously^54^. Briefly, relevant antibodies were conjugated to lanthanide metals using the Maxpar X8 antibody labelling kit. 4 µm thick formaldehyde-fixed paraffin-embedded (FFPE) sections were deparaffinized and incubated for 40 minutes in EnVision FLEX Target Retrieval Solution High pH (DAKO). Prior to staining liver sections were blocked by incubation with SuperBlock Blocking Buffer (ThermoFisher). Once blocked, slides were incubated with the relevant conjugated antibodies at R.T. overnight (detailed in Supplementary Table 2) in 0.5% BSA, 1% Triton-X-100 in TRIS. 30 minute exposure to Iridium Cell-ID intercalator (Fluidigm) was used to visualize DNA. Ablation/imaging was conducted using the Hyperion Imaging Mass Cytometry system (Fluidigm).

Data analysis was performed using ImageJ, *R* Studio with *imctools, QMIQ* and *cytomapper*^54^. The Liver Zonation Index was calculated by importing relevant tissue section images into ImageJ (Version 1.52) using the *imctools* plugin, and custom code accessible here: https://github.com/ljpallett/pallettetal_2022_imc. A Gaussian Blurr filter was applied to the CYP1A2 signal with a sigma of 100 and the intensity of each pixel measured. Background subtraction was performed by subtracting the minimum intensity per image from all values. Areas with a liver zonation index ≥ 0.5 were classified as zone 3; < 0.5 and ≥ 0.25 zone 2; and < 0.25 as zone 1. Enrichment scores were calculated by comparing the absolute cell counts in a certain zone to a hypothetical cell count if there was an equal distribution of the respective cells across all zones. The enrichment ratio was calculated by dividing the enrichment score of one cell population by the enrichment score of another cell population in a given zone. The distance to the closest myeloid structure was obtained by calculation of the distance to the closest Iba1^+^ myeloid structure in ImageJ.

### 3D de-cellularised liver scaffolds

For the preparation of de-cellularised scaffolds, liver tissue was obtained through the *TAPb Initiative*; study approval reference: 11/WA/0077. The full protocol for the development of 3D acellular biological scaffolds was previously described^13^. Briefly, whole human livers were perfused with 1x PBS to remove blood and frozen at -80 ºC prior to decellularization. The right lobe was sectioned and agitated with de-ionised water (Merck-Millipore) containing 3% sodium deoxycholate (Sigma Aldrich; Merck), 0.5% sodium dodecyl sulfate (Sigma Aldrich; Merck), 0.3% Triton X100 (ThermoFisher Scientific) and 4.5% sodium chloride (Sigma Aldrich; Merck) and 1x PBS until the tissue was translucent with the dissolution of cells. The absence of cells in the extracellular matrix proteins (ECM) scaffolds was confirmed by the absence of DNA material quantified by Dneasy Blood and Tissue Kit (Qiagen). All scaffolds were confirmed by proteomics and immunohistochemical analysis to express natural ECM including collagen-I, -III and -IV, fibronectin, laminin, lumican, mimecan and vitronectin^55^. 0.5-1x10^6^ PBMC were cultured in cRPMI in the presence 40 IU/mL recombinant human IL-2 and 5 ng/mL IL-15 (BioTechne) for 3 d in 96-well round-bottom plates ± an ECM scaffold. After culture, 3D scaffolds (and cells cultured in the absence of a 3D scaffold) were subjected to 30 min collagenase digestion to recover leukocytes for phenotypic analysis as described in *Multiparametric flow cytometry*.

### Quantification of Gene Expression by RT-PCR

For the quantification of mRNA transcript for CD14, TLR4 and MD-2, total RNA was extracted from highly pure paired *ex vivo* human intrahepatic CD14^-^CD8^+^T-cells or CD14^+^CD8^+^T-cells using TRIzol and RNAeasy Plus Micro Kit (Qiagen). Subsequently RNA was reverse-transcribed to cDNA with the High-Capacity RNA-to-cDNA Kit (Applied Biosystems) or Quantitecht RT Kit (Qiagen). Gene expression was measured by quantitative rtPCR using the 7500 Real-Time PCR System (Applied Biosystems). KiCqStart PCR primers used were as follows: 18S, CD14, TLR4, MD-2 (Sigma Aldrich; Merck). The relative expression level of specific transcripts was normalized with respect to the internal standard, 18S and calculated using the delta Ct method^56^.

### Single-cell RNA-sequencing: Samples

For the analysis of the single cell transcriptome two intrahepatic perfusate samples were isolated and frozen at -80 °C for storage prior to transportation to Newcastle, UK. Once thawed in pre-warmed HBSS containing 0.001% DnaseI, intrahepatic leukocytes were sorted by FACS to index classical mononuclear phagocytes (MNP) defined as Singlets, CD3^-^CD56^-^ CD19^-^CD14^hi^HLA-DR^+^ or CD14± CD8^+^T-cells as follows: Singlets, CD3^+^CD56^-^CD19^-^CD4^-^ CD8^+^Vα7.2^-^ and split in to CD14± based on an isotype control. Single cells were sorted into 96-well skirted LoBind plates (Eppendorf) containing 5 µL lysis buffer (TCL buffer [Qiagen], supplemented with 1% β-mercaptoethanol [Sigma Aldrich; Merck]) on a BD Bioscience FACSFusion. The sorted plates were subsequently immediately sealed, centrifuged, and placed on dry ice to flash-freeze the cell lysate and stored at -80 °C.

### Single-cell RNA-sequencing: SMART-seq2 library preparation and sequencing

A modified SMART-seq2 protocol^57^ was performed on the single flow cytometry sorted-cells as previously described^58^. After cDNA generation, libraries were prepared (384 cells per library) using the Illumina Nextera XT kit (Illumina). Each library was sequenced to achieve a minimum depth of 1-2 million raw reads per cell using an Illumina HiSeq 4000 using v. 4 SBS chemistry to generate 75-bp paired-end reads.

### Single-cell RNA-sequencing: Alignment, quantification and quality control

SMART-seq2 sequencing data were aligned with STAR (v. 2.5.1b) using the GRCh38 human reference genome. Quantification was performed using htseq-count (v. 0.10.0). Cells with fewer than 200 detected genes, more than 2750 genes, or for which the total mitochondrial gene expression exceeded 20% were removed as previously described^58^. Genes expressed in fewer than three cells were removed. The simulated doublet histogram generated using Scrublet was unimodal, likely due to cellular homogeneity^59^. Harmony data integration^60^ was used to correct for any batch effects between samples.

### Single-cell RNA-sequencing: Clustering and annotation

Following quality control gene expression was normalised by cell to correct for cell-to-cell variation in total reads and log transformed using the *NormalizeData* tool in Seurat. The top 2000 highly variable genes were identified using the “*vst*” method in Seurat. Linear dimensional reduction was performed by principal component analysis after expression levels were scaled to mean expression 0 and variable 1 across all cells. The dimensionality of the data was determined using the *JackStraw* procedure in Seurat and the top 30 principal components used to generate the neighbourhood graph. Clustering was performed using the Leiden algorithm (0.6 resolution) and embedded using Uniform Manifold Approximation and Projection (UMAP).

### Dextramer staining for the identification of virus-specific CD8^+^T-cells

In study participants known to be HLA-A2^+^ (confirmed by monoclonal HLA-A2-FITC antibody [BioRad; Abcam] staining), HBV-specific or CMV-specific APC-conjugated HLA-A2/dextramers (Immudex) of the following specificities were used: HBVcore18-27 (FLPSDFFPFV), HBVenvelope183-191 HBVenvelope335-342 (WLSLLVPFV), HBVenvelope348-357 (GLSPTVWLSV), HBVpolymerase455-463 (GLSRYVARL), HBVpolymerase502-510 (KLHLYSHPI) or CMV-NLV (NLVMVATV). To identify antigen-specific CD8^+^T-cells, isolated intrahepatic leukocytes were stained with corresponding dextramers at 37 °C in 1x PBS for 20 min, washed twice in cRPMI, and left to rest for 1 hr before further mAb staining as described in *Multiparametric flow cytometry*.

### FITC-LPS uptake assay

To assess the functionality of the TLR4:CD14 complex 0.5-1 x 10^6^ freshly isolated IHL or stromal cell induced CD14-expressing CD8^+^T-cells (described above: *Isolation of primary human hepatic stellate cells and co-cultures with PBMC/CD8*^*+*^*T-cells*) were cultured in warm cRPMI in the presence or absence of 5 µg/mL fluorescently-labelled LPS (ThermoFisher Scientific) at 37 °C for 30, 60 or 120 min prior to flow cytometric staining to determine LPS-uptake by the cell by flow cytometric assessment of the mean fluorescence intensity (MFI) of the FITC channel.

### Measurement of Supernatant Cytokine Profiles by Luminex Assay

To functionally profile CD14-expressing CD8^+^T-cells we used a multiplex array to profile stromal cell induced CD14± CD8^+^T-cells (described above: *Isolation of primary human hepatic stellate cells and co-cultures with PBMC/CD8*^*+*^*T-cells*). CD14± CD8^+^T-cells were sorted on a BD FACSAriaII to exclude MNP by FSC-A and SSC-A, dead cells and doublets, then sorted for CD3^+^CD56^-^CD4^-^CD8^+^Vα7.2^-^ and split in to CD14± based on an isotype control. Purified CD14± CD8^+^T-cells were seeded in 96-well round-bottom plates at a density of ˜1 × 10^5^ cells/well in 200 µL cRPMI and cultured at 37 °C for 24 hr in the presence or absence of exogenous stimulation with ˜0.3 × 10^6^ UV-killed *E. coli* or 1 µg/mL immobilised anti-CD3 and 5 ng/mL soluble anti-CD28 (both eBioscience; ThermoFisher Scientific). After incubation cell culture supernatants were harvested and stored at -80 °C for multiplex analysis. Cytokine expression profiles were determined using the Luminex array platform with the Human XL Cytokine Luminex 45-plex Performance Assay (BioTechne). Samples were acquired on a MAGPIX Luminex (ThermoFisher Scientific) running xPONENT v. 4.2 and analysed using a custom pipeline (Python v. 3.6; https://github.com/ljpallett/pallettetal_2022_luminex).

### In vitro experiments: phenotype and functionality

To assess the effect of *in vitro* bacterial LPS-exposure on CD14 expression 0.5-1 x 10^6^ freshly isolated IHL from human resected tissue were cultured in the presence or absence of ˜0.3 × 10^6^ UV-killed *E. coli* for 24, 48, 72, or 96 hr in cRPMI. Phenotype of CD8^+^T-cells was assessed by flow cytometry as above.

To assess the functionality of intrahepatic CD14-expressing T-cells *ex vivo* 1 x 10^6^ IHL were stimulated with 1 µg/mL immobilised anti-CD3 and 5 µg/mL soluble anti-CD28 for 4 hr at 37°C in the presence of 1 µg/mL brefeldin-A (Sigma Aldrich; Merck). Functionality was assessed by intracellular cytokine production as described in *Multiparametric flow cytometry*.

To confirm the capacity of stromal cell induced CD14± CD8^+^T-cells (described above: *Isolation of primary human hepatic stellate cells and co-cultures with PBMC/CD8*^*+*^*T-cells*) to secrete cytokines/chemokines we used intracellular cytokine staining. Freshly isolated peripheral CD8^+^T-cells were first co-cultured with autologous MNP 1:1 for 4 d. After co-culture ± CD8^+^T-cells were removed and re-seeded in 96-well round-bottom plates in cRPMI supplemented with 100 IU/mL IL-2 and cultured at 37 °C for 24 hr in the presence or absence of exogenous stimulation with ˜0. 3 x10^6^ UV-killed *E. coli* and brefeldin-A. Strict gating including an αβTCR antibody was used to exclude MNP from analysis.

### CFSE-labelling and dual CD14-labelling of leukocytes

Where indicated, freshly isolated intrahepatic leukocytes were pre-labelled with CD14-APC (ReaAffinity; Miltenyi Biotech) and 2 µM CFDA (CFSE; Invitrogen; ThermoFisher Scientific) for 15 min at 37 °C prior to 3 d culture in the presence of ˜0.3 × 10^6^ UV-killed *E. coli* to determine cellular proliferation. After culture, leukocytes were further stained with CD14-PE (BioLegend) along with other surface mAb to determine phenotype as described in *Multiparametric flow cytometry*.

### Human in vivo skin blister model

The human *in vivo* skin blister model was approved by UCL Research Ethics Committee (reference: 10527/001). Seven young healthy volunteers were recruited. Intradermal UV-killed *E.coli* (UV-killed *E. coli*; strain: NCTC10418; source: Public Health England, UK) was used to model *in vivo* gram negative bacteria exposure using suction blisters to induce an acute, resolving inflammatory response for interrogation as previously described^41^. Briefly 1.5 × 10^7^ UV-killed *E. coli* in 100 µL of sterile saline was injected intradermally into a marked site on the ventral side of the right forearm of participants. From each participant the left forearm was used to raise a “naïve” unexposed blister to analyse the exudate (baseline blister). After 24 hr each participant returned, and a suction blister was used to remove the inflammatory exudate from the LPS-exposed injection site from the right forearm. Both blister exudates were centrifuged to concentrate the cells and analysed as described in *Multiparametric flow cytometry*.

### Generation of TCR-redirected T cells

Where indicated, genetically engineered CD8^+^T-cells with known TCR specificity were used to generate CD14-expressing T-cell *in vitro*. For this CD8^+^T-cells were modified to express a TCR specific for an immunodominant epitope of the HBV envelope protein; Hbe183-91 (e183-specific CD8^+^T-cells; amino acid sequence: FLLTRILTI). For this phoenix amphotrophic packaging cells (ATCC CRL-3213) were transiently co-transfected using FuGENE (Promega) with plasmids encoding for the e183-specific TCR alongside an amphotrophic envelope protein. For these transfected phoenix cells, retroviral supernatants were collected for the further transduction of healthy donor CD8^+^T-cells obtained from freshly isolated PBMC by MACS-bead isolation (Human CD8^+^ isolation kit). Prior to transduction isolated CD8^+^T-cells were stimulated with Human T-Activator CD3/CD28 Dynabeads (ThermoFischer Scientific) in cRPMI supplemented with 5 ng/mL recombinant human IL-15 and IL-7 (PeproTech) and 100 IU/mL IL-2 for up to 48 hrs. For transduction: 1 × 10^6^ activated CD8^+^Tcells/well were plated on retronectin-coated (Takara) plates and mixed by centrifugation with previously harvested and concentrated retroviral supernatants. Transduced CD8^+^T-cells were expanded in cRPMI supplemented with 5ng/mL recombinant human IL-15/IL-7 and IL-2 as above for up to 10 d and continually monitored for the expression of the engineered e183-specific TCR by staining for the murine constant region of the TCRβ chain by flow cytometry prior to co-culture with autologous MNP/MDM (as described in *Isolation of mononuclear phagocytes [MNP] and the in vitro generation of monocyte-derived macrophages [MDM])*.

After gene-modification and expansion, TCR-redirected CD8^+^T-cells were co-cultured with autologous MNP/MDM at a ratio of between 1:1 and 10:1 (T-cells:MNP; depending on MNP availability) for 4 d to allow for T-cell acquisition of the CD14/TLR4 complex. After MNP/T-cell co-culture e183-specific TCR-redirected CD14± CD8^+^T-cells were sorted on a BD FACSAriaII to exclude MNP by FSC-A and SSC-A, dead cells and doublets, then sorted for CD3^+^CD56^-^CD19^-^ CD4^-^CD8^+^Vα7.2^-^murineTCRβ^+^ and split in to CD14± based on an FMO control.

### preS1 HepG2 maintenance

A human HCC cell line, HepG2, transduced with a construct containing the preS1 portion of the genotype D HBV envelope protein (HepG2-preS1 cells) using the Lenti-X HTX packaging system, was kindly provided by Prof A. Bertoletti’s lab (Singapore). HepG2-preS1 cells were cultured in DMEM (Invitrogen) supplemented with 10% HI-FBS, 100 IU/mL penicillin/streptomycin, 0.5 mM sodium pyruvate and MEM non-essential amino acids. The HepG2-preS1 are capable of endogenous expression of HBV envelope protein, recognised by the specific engagement of the e183-specific TCR, as previously described.^61^ The HepG2-preS1 line used in this study was regularly tested throughout for mycoplasma contamination by PCR (EZ-PCR Mycoplasm Kit, Biological Industries).

### Assessment of functionality of TCR-redirected T cells

To evaluate the function of the TCR-redirected CD8^+^T-cells, HepG2-preS1 cells were plated and allowed to adhere at various cell densities (as indicated in the appropriate figure) for 6 hr prior to use. Once adhered, FACS-sorted e183-specific TCR-redirected T cells ± CD14 were added directly to the HepG2-preS1 cells in cRPMI supplemented with 100 IU/mL IL-2 for 16 hrs, either in the presence or absence of BFA/monensin. The CD8^+^T-cells cultured in the presence of BFA/monensin were harvested after the 16 hr incubation and assessed for cytokine production by intracellular cytokine staining as described in *Multiparametric flow cytometry*. Whereas the cell-free supernatant was harvested from the CD8^+^T-cells cultured without BFA/monensin and assessed for cytotoxicity using the ToxiLight Non-destructive Cytotoxicity BioAssay (Lonza) as per the manufacturer’s instruction. Briefly the assay evaluates the specific target cell lysis by measuring relative light units (RLU), using the following calculation: (RLU [effector + target] – RLU [effector] – RLU [target]) / (RLU [100% target lysis] – RLU [effector] – RLU [target]) * 100.

## Statistical analyses

Statistical analyses were performed in Excel v.16.16.09, Prism (GraphPad v.7 or v. 8) or Python 3.6 using appropriate tests (Mann-Whitney *t* test, Wilcoxon Signed-rank *t* test, Kruskal-Wallis test [ANOVA] with Dunn’s post hoc test for pairwise multiple comparisons between each group) as indicated in the legends. The following tests were carried out as two-tail tests with significant differences marked on all figures. Where appropriate, the Bonferroni correction method for multiple testing was used. Error bars display S.E.M. Significance levels were marked on all figures. Relevant visualisations were created using Python 3.6 and seaborn, the statistical visualisation package.

## Extended Data

## Supplementary Material

Peer Review File

Reporting Summary

Supplementary information

## Data Availability

The source data used within the main figures is available as supplementary files to this manuscript and from figshare (DOI: https://doi.org/10.6084/m9.figshare.21623379). scRNAseq data has been deposited at the European Genome-phenome Archive (EGA), which is hosted by the EBI and the CRG, under accession number EGAS00001006885. Further information about EGA can be found on https://ega-archive.org. Further datasets generated during and/or analysed as part of this study are available from the corresponding authors upon reasonable request.

## Code availability

Custom codes used in this study are available on GitHub. For the heat map visulisation used to display the soluble mediator profiles of CD14-expressing CD8^+^T-cells the custom code created is available: https://github.com/ljpallett/pallettetal_2022_luminex.

For the image mass cytometry analysis, the custom code created is available: https://github.com/ljpallett/pallettetal_2022_imc.
