# Supplementary material for: Tissue CD14+CD8+T-cells reprogrammed by myeloid cells and modulated by LPS
Source: Nature. Author manuscript; Available in PMC 2026 Aug 12. (PMC7619353; doi:10.1038/s41586-022-05645-6)
Supplement: Supplementary file 1 [file EMS216202-supplement-Peer_Review_File.pdf]

Peer Review File

Manuscript Title: Tissue CD14+CD8+T-cells are reprogrammed by myeloid cells and modulated by LPS

Reviewer Comments & Author Rebuttals

Reviewer Reports on the Initial Version:

Referees' comments:

Referee #1 (Remarks to the Author):

"Bacterial LPS drives tissue-sequestered immunomodulatory CD14+CD8+ T cells" describes a subset of liver (and skin) resident CD8 cells that have a regulatory phenotype (IL-10 production, Foxp3+ expression) and are distinguished by expression of the surface LPS receptor (CD14, TLR4, MD2). These cells have an activated phenotype, are spontaneously secreting large amounts of regulatory cytokines (detectable ex vivo without brefeldin), have increased expression of metabolic receptors, and have constitutive PD1 expression. CD3 stimulation drives inflammatory cytokine production, indicating their regulatory phenotype can be overcome. Repertoire analysis and staining with tetramers indicates that there is not an obvious bias in specificities between the CD14+ and CD14- compartments. Stimulation in vitro or in vivo (in human skin and in mice) with LPS expands the CD14 compartment as a proportion of the populations and (in some experiments) in overall cell number. The cell type reported is interesting and the data presented are mostly robust. Foxp3+ CD8 T cells have been described in multiple systems, but the link to LPS-responsiveness and CD14 expression provides a novel insight into their function and, potentially, their ontogeny. The primary concern with the manuscript is that in its current form it fails to resolve either of these issues (function, ontogeny) definitively. None of the functional experiments demonstrate the role of this cell type in tissue protection or provide an example of under what conditions their regulatory role is essential. The huPBL mouse model might allow some manipulation (Foxp3 knockdown or CRISPR) to address this. Alternatively, describing the ontogeny of these cells would be useful, but the LPS stimulation assays only demonstrate that this signal likely expands this population once it is CD14+--not how it differentiates into a CD14+ regulatory population to begin with.
Other specific concerns:
1) In a pure culture of CD14- cells, following CD3/CD28 stimulation, do any of the cells upregulate CD14? What about under different biasing conditions with cytokine stimulation?
2) Related to 1, the metabolism phenotype is intriguing and might point to a mechanistic role of metabolic signals in driving the differentiation of this subset—can this be recapitulated in vitro (by feeding or starving these cells of various metabolites) to drive differentiation?
3) The high levels of constitutive PD1 expression are suggestive of ongoing TCR stimulation. Is there any other evidence that TCR is being stimulated (transcriptional, functional) in these cells?
4) Figure 3g is a very nice experiment—elegant and informative.
5) The repertoire data are interesting, but the presence of TCRs and tetramer positive cells in both CD14+ and CD14- subsets would suggest that antigen is not driving the differentiation alone. Also, this complicates the interpretation of the PD1 expression—for the tet+ cells that are in both compartments, are the CD14- cells PD1 negative (suggestive that antigen is not around?)? This

would help resolve the point in 3), whether PD1 is being driven by TCR signaling directly.
6) The repertoire analysis seems like it would be useful to apply to the LPS-expanded cultures to determine if LPS is non-specifically activating the entire CD14+ population or further selecting a specific subset.
7) For the effector cytokine production, given the ease with which epitope-specific cells were identified (CMV, HBV), stimulation with cognate peptide would be preferable (and would potentially be more informative functionally) than the extraphysiological signal of CD3/CD28.
8) Figure 1c: This is difficult to read, there are too many data points on one graph. Jitter-ing the points, enlarging the graph, or choosing a different color scheme would be helpful.
9) Figure 5 and b need to also include absolute numbers of cells after culture. Including BrdU in the in vitro culture would also help show what proportion of the CD14+ cells expand.
10) It seems like more animals need to be included in 5j—the spread is too big and the power too low in its current form.
11) 5J through l (the mouse huPBL experiment) would also be improved by including a BrdU incorporation assay.
12) Minor point: in extended data figure 3e, the enriched sequences in the TRBV7-8 BJ2-7 have a strong motif, mostly driven by the V and J germline sequences. The central T residue though is a rare occurrence in the 7-8/2-7 sequences in the vdjdb and may represent a non-templated addition. This would be easy to determine and would be an interesting side note further suggesting a strong signal of antigen-specific enrichment.
13) There's a generic comment about code and data availability that should be made specific. The sequencing data could be deposited and an accession number reported during the review process.

Referee #2 (Remarks to the Author):

This manuscript reports that human CD8+ T cells expressing CD14 and compartmentalized in the liver - and possibly in other organs exposed to bacterial products - have constitutive immunoregulatory features (expression e.g. of Foxp3, CTLA-4 and IL-10) but can rapidly mount effector responses (production of IFN-g, TNF) upon TCR stimulation. CD14+CD8+ T cells co-express TLR4, are rare in blood while are present in variable proportions in human liver samples from healthy tissues or in liver biopsies from patients with chronic HBV infection or other liver diseases, and can develop in HIS mice engrafted with human fetal liver-derived stem cells. Exposure to LPS or Gram-negative bacteria results in an increased proportion of CD8+CD14+ T cells, in vitro and in vivo, while administration of antibiotic against Gram-negative bacteria results in a reduced frequency of circulating and intrahepatic human CD8+CD14+ T cells in NGR mice. The experiments are of high technical quality and some of the findings are novel and exciting. However, there are limitations inherent to the experimental systems and hence difficulties in drawing conclusions on a mechanistic link between bacterial LPS, CD14+CD8+ T cells and the balance of hepatic tolerance versus immunity. The data provided raise a number of issues that could be addressed by the authors to strengthen the manuscript, as specified below.
Specific points:
1. A major issue is that the authors do not provide evidence that CD14/TLR4 is functional in liver CD8+ T cells. Other studies have shown that CD14 and TLR4 can be expressed on the surface of

human CD8+ T cells and that these cells can directly respond to TLR4 agonists, while TLR4 knock down by siRNA attenuates this response (Komai-Koma et al., 2009).
2. Another major issue is that most of the findings in the manuscript do not necessarily relate to a direct effect of LPS on CD8+CD14+ T cells, given that experiments of LPS stimulation are not done on purified cells. In these conditions, it cannot be excluded that the effects are indirect and mediated by other LPS-sensing cells. If this was the case, it would still be an interesting, albeit different, report. This points needs to be clarified, for example using the same in vivo model of adoptive transfer of human T cells into NRG mice and taking advantage of the CRISPR/Cas9 system to knock out CD14 gene, while simultaneously knocking in GFP gene as a reporter for CD14 promoter activity.
3. Phenotypically, CD14+CD8+ liver T cells express Foxp3, CD25, CTLA-4, LAG3, which are characteristic of Treg cells but also of activated conventional human T cells. These findings raise questions as to the stability of the described phenotype.
4. The authors need to clarify the clonal relatedness between CD14+ and CD14– CD8+ T cells, especially in light of the findings that these two populations share antigen specificity and TCR usage. The TCR sequencing data indeed support the notion that the two cell populations do not represent distinct lineages rather they may represent functionally distinct stages or different fates from a common precursor.
5. The data displayed in Fig. 3g of donor-derived a liver allograft explant are remarkable. However, it is difficult to conclude that CD14+ T cells are long lived, since one cannot exclude the possibility that these cells are continually generated from a CD14– population. There is also a different proportion of CD14+ cells of donor or recipient origin. This finding needs to be commented.

Referee #3 (Remarks to the Author):

The authors describe a CD8 T cell subset found in liver that displays both immunoregulatory and proinflammatory characteristics by expressing a Treg signature (i.e., FoxP3, CTLA4, IL10 and IL2) and effector cytokines (IFNg and TNFa). TCR repertoire analysis indicates that these cells are differentiated from common progenitors with CD14– CD8 T cells or CD14– liver resident CD8 T cells and may obtain CD14 expression upon particular stimuli. These cells can be expanded by gram-negative bacteria and LPS, suggesting the functional significance of CD14 expression by these tissue-resident CD8 T cells. While identification and characterization of tissue-resident CD8 T cells that potentially possess a specialized function may be interesting, this study lacks novelty because the majority of the data presented here have been described in previous studies by these and other authors (Pallett LJ JEM 2017, Komai-Koma M EJI 2009). Furthermore, these studies lack data that show the functional attributes of these CD8 T cells in the tissue environment. A potentially new and relevant aspect of this study would have been the immunoregulatory capacity of this CD8 T cell subset during pathogen invasion to preserve tissue homeostasis. However, the authors did not explore this critical feature experimentally and only speculate on their functionality according to expression of surface molecules and cytokine production. Overall, while this study has investigated the phenotype of liver-resident CD14+ CD8 T cells in more detail compared to previous reports, neither their contribution to tissue homeostasis nor their contribution to liver inflammation have been investigated, which limits the impact of this study in understanding these tissue resident CD8 T cells.

Specific comments:

1. The authors suggest the immunomodulatory potential of CD14+CD8+ T cells based on their expression of FOXP3, CD25, CTLA4, LAG3 etc (Fig. 2). Although these molecules belong to the CD4 Treg signature, expression by a subset of T cells also reflects the state of activation. To claim that they are regulatory CD8 T cells in the liver tissue environment, their suppressive activity should be demonstrated.

2. The authors state that CD14+CD8 T cells express proinflammatory effectors IFNg, TNFa and MIP1b upon TCR stimulation while maintaining IL10 and IL2. From the data in Fig. 2c, it is clear that the IL10 producing CD14+CD8+ T cells co-express IL2. However, the tSNE plots in Fig. 2f show that IFNg and TNFa producing cells belong to a very minor fraction of IL10/IL2 producers, which do not support their statement that the CD14+CD8+ T cells function as both regulatory and effector T cells. The data shown do not provide convincing evidence that these opposite roles are exerted by this liver resident CD14+CD8 T cell subset depending on distinct immune conditions (tissue homeostasis vs, pathogenic challenge).

3. Fig. 3 is devoted to another set of characterization of surface marker expression by CD14+CD8+ T cells, some of which was described in their previous report (JEM, 2017). While the majority of markers expressed by CD14+CD8 T cells that are associated with activation and cytotoxic activity are expressed at similar levels as CD14– CD8 T cells, some markers that are upregulated by CD14+CD8 T cells, including CD71, CD98 and CD36, indicate they are metabolically active. While this selective marker expression pattern may reflect a specialized function of this cell type, this study does not go beyond phenotype analysis and it is therefore not clear whether the acquisition of this phenotype by CD14+CD8 T cells is critical for their contribution to the immune response in liver tissue.

4. Fig. 3g: the authors intended to examine the longevity of CD14+CD8 T cells using liver allograft obtained from an HLA-mismatched recipient. The FACS profile presented, however, indicates that the authors failed to detect donor derived HLA-A2+ cells, in contrast to the statement in the ms. In general, HLA-A2 Abs can detect a clear population if they exist. On the other hand, even if successful, this experiment would not lead to the conclusion that CD14+CD8 T cells are long-lived. There is also no evidence that CD14+ CD8 T cells are stable and this fraction could potentially have been differentiated from progenitors after transplantation. Therefore, this experiment is not appropriate to test the longevity of CD14+ CD8 T cells.

If any, the data in Fig. 3g is also not consistent with data shown in the previous figures: ~15% of endogenous intrahepatic CD8 T cells in recipients should express CD14, if their basic analysis shown in Fig. 1a is representative. Here, the authors show that CD14+ CD8 T cells are found only in the donor-derived fraction.

5. Fig. 5: to analyze the physiological relevance of CD14 expression by tissue resident CD8 T cells, the expansion of CD14+CD8 T cells after stimulation with LPS was analyzed in vitro using bulk intrahepatic leukocytes. Although the binding of LPS to CD14 on CD8 T cells may have stimulated their expansion, this effect could also be due to increased activation of APCs. Earlier studies by Komai-Koma showed that purified CD8 T cells can be stimulated with LPS to induce perforin and

granzyme. Therefore, in principle, purified CD14+CD8 T cells can directly respond to LPS or gram-negative bacteria. However, the current data may include both direct and indirect effects of LPS in the presence of potentially CD14hi non-CD8 cells.

6. Fig. 5k: In the analysis of CD8 T cells recovered from NRG mice reconstituted with human CD3+ T cells, the authors show that LPS stimulation upregulates IL10 and IL2 production while almost all CD14+ CD8 T cells produce IFNg. It is not clear why CD14+ CD8 T cells express this high level of IFNg in this adoptive host. In this figure, cytokine (IL10, IL2, IFNg) production is depicted by percentage while expression of FoxP3 is presented by cell numbers. Is there a reason for this inconsistency; it would be more useful to show the percentage for FoxP3.

In this analysis, CD8 T cells express both immunomodulatory and effector molecules. What is the functional significance of this dual phenotype for tissue immune homeostasis in this engraftment model?

Referee #4 (Remarks to the Author):

The current study by Pallett et al. describes a new subset of CD8+ T cells in the liver and proposes a contribution of these cells to the immunomodulatory state of the liver. The immunomodulatory function of the liver is an important topic with respect to the body's physiology and within clinical settings like organ transplantation, chronic infection and cancer. So far, the underlying mechanisms are poorly understood. Using unique human sample materials complemented by experiments in humanized mice, Pallett et al. were able to provide an interesting T cell subset, CD14+ CD8+ T cells, within the complex puzzle of modulators of the immune response within the liver. However, the overall concept behind the described CD14+ CD8+ T-cell population within the liver is weakly defined and thus somewhat preliminary and needs further clarification. Specific points are listed below:

- Do CD8+ CD14+ T cells express CD8a/a or CD8a/b?

- Is CD14+ expression specific for CD8+ T cells in the liver? Or do e.g. CD4+ T cells or NK cells also express CD14?

- The authors provide mRNA data of CD14, TLR4 and MD2 to confirm intrinsic expression. However, relation to mRNA expression levels in CD14+ myeloid cells are missing.

- CD14+ CD8+ T cells seem to consist of different subpopulations given the bimodal expression of multiple surface markers and that some cells are virus-specific. What does this mean in terms of the differentiation/activation status of these cells? Are there any specific co-expressions of distinct markers found (e.g. in t-SNE analyses)? Along this line, what is about the plasticity/the dynamics of the immunomodulatory CD14+ CD8+ T cells? Some epigenetic analyses and if possible an exemplified longitudinal analyses would provide further insights into these questions. In addition, epigenetic analyses in comparison to CD14-CD8+ T cells and CD14+ myeloid cells would provide insights into the relationship of CD14+CD8+ T cells to both cell types.

- CD14+CD8+ T cells are present in the liver and in the skin. Does this mean that these cells are tissue-specific subpopulation and cannot be found in the circulation? Are these cells a subset of tissue-resident T cells? However, at least not all are tissue-resident and therefore CD14 is not a unique feature of tissue-resident CD8+ T cells. Does this mean that the CD14+ CD8+ T cells are replenished from the circulation by CD14- CD8+ T cells? Please comment on that. Along this line, given that CD14+CD8+ T cells are proliferative and long-lived, is there a difference in Ki67 and BCL-2 expression between CD103+CD69+ and CD103- CD69+ CD14+ CD8+ T cells? This may reflect distinct differentiation/maturation sates of the CD14+ CD8+ T cells.

- Fig. 3g: A high proportion among the donor-derived cells are CD14+ CD8+ T cells. This implies that a high proportion of tissue-resident CD8+ T cells are CD14+ and thus immunodmodulatory in the liver? What is the fraction of tissue-resident CD8+ T cells in the liver that express CD14? Is the TCR repertoire similar distributed among tissue-resident and CD14+ CD8+ T cells or are CD14- and CD14+ tissue-resident CD8+ T cells comprised of distinct clones?

- Do the authors have any ideas about the triggers inducing CD14+ CD8+ T cells? According to the HIS model, is homeostatic proliferation one factor? Performing a similar transfer of murine liver-derived stem cells into NRG mice would further clarify this point. Furthermore, please at least discuss whether there is an impact of the SPF condition or specific microbiota (not load) on the induction of CD14+ CD8+ T cells. The increased fraction of CD14+ CD8+ T cells in E.coli cultures does not provide evidence for intrinsic induction or expansion of CD14+ CD8+ T cells since CD14 could be taken up from CD14+ cells within leukocytes. Thus, CD14 transcript levels should also be assessed in CD14+ CD8+ T cells after E.coli stimulation. In the skin blister model, absolute cell counts of CD14-/CD14+ CD8+ T cells as well as assessing CD14 mRNA levels will further clarify whether CD8+ T cells differentiate into CD14+ CD8+ T cells or are recruited or expand. The applied experimental systems do also not address the question whether LPS or other E.coli-derived PAMPs directly affect CD14+CD8+ T cells or whether this are indirect effects mediated by accessory cells. Please elaborate this further, e.g. by directly stimulating or co-stimulating sorted CD14- vs. CD14+ CD8+ T cells with LPS/E.coli-derived PAMPs and testing proliferation and effector functions. This would also provide further insights into the role of CD14 in the context of TCR stimulation.

- To name CD14+ CD8+ T cells "innate-like" seems to be misleading given that some of the cells are virus-specific. Is there evidence for any "master regulator" of innate-like T cells that is also expressed by CD14+ CD8+ T cells, e.g. PLZF? Based on the TCR repertoire analyses the authors should extend the detection of semi-invariant T cells beyond MAIT cells. To what extent are semi-invariant T cells present in CD14+ compared to CD14- CD8+ T cells?

- It remains unclear whether the presence of CD14+ CD8+ T cells is specific for the liver and thus provide a unique immunomodulatory rheostat within the gut-liver axis. As shown by the authors these cells can also be found in the skin blister model clearly questioning their restriction to the liver. Analyzing diverse organs of HIS mice will further clarify whether CD14+CD8+ T cells are also present in other barrier tissues like the gut, skin, lungs or organs like the kidneys and salivary glands or even lymphoid tissue like the spleen and lymph nodes.

Author Rebuttals to Initial Comments:

Referee #1 (Remarks to the Author)

"Bacterial LPS drives tissue-sequestered immunomodulatory CD14+CD8+ T cells" describes a subset of liver (and skin) resident CD8 cells that have a regulatory phenotype (IL-10 production, Foxp3+ expression) and are distinguished by expression of the surface LPS receptor (CD14, TLR4, MD2). These cells have an activated phenotype, are spontaneously secreting large amounts of regulatory cytokines (detectable ex vivo without brefeldin), have increased expression of metabolic receptors, and have constitutive PD1 expression. CD3 stimulation drives inflammatory cytokine production, indicating their regulatory phenotype can be overcome. Repertoire analysis and staining with tetramers indicates that there is not an obvious bias in specificities between the CD14+ and CD14- compartments. Stimulation in vitro or in vivo (in human skin and in mice) with LPS expands the CD14 compartment as a proportion of the populations and (in some experiments) in overall cell number.
The cell type reported is interesting and the data presented are mostly robust. Foxp3+ CD8 T cells have been described in multiple systems, but the link to LPS-responsiveness and CD14 expression provides a novel insight into their function and, potentially, their ontogeny. The primary concern with the manuscript is that in its current form it fails to resolve either of these issues (function, ontogeny) definitively. None of the functional experiments demonstrate the role of this cell type in tissue protection or provide an example of under what conditions their regulatory role is essential. The huPBL mouse model might allow some manipulation (Foxp3 knockdown or CRISPR) to address this.
Alternatively, describing the ontogeny of these cells would be useful, but the LPS stimulation assays only demonstrate that this signal likely expands this population once it is CD14+--not how it differentiates into a CD14+ regulatory population to begin with.
Thank you for comments on interest, robustness and novelty of LPS-responsiveness in immunomodulatory tissue CD8^{+}T-cells. We have further investigated the function and, predominantly, the derivation of CD14^{+}CD8^{+}T-cells in humans, see *Overall Comments* section above.

Other specific concerns:

1) In a pure culture of CD14- cells, following CD3/CD28 stimulation, do any of the cells upregulate CD14?
What about under different biasing conditions with cytokine stimulation?
We have now shown that activated primary hepatic stellate cells, 3D liver extracellular matrix scaffolds or CXCL12 can induce CD14 on CD8^{+}T-cells when stimulated in the presence of PBMC (**Fig.2c-e**). Other cytokines/chemokines tested (IL-6, IL-8, CXCL10) did not mimic the effect of CXCL12 (**Fig.2e**). The effect of hepatic stellate cells was abrogated when using pure CD8^{+}T-cells (**Fig.2f**), implicating a possible role for MNP within the PBMC culture. We have also extended our dataset to show that TLR4 ligand monophosphoryl lipid A (MPLA) in addition to UV-killed *E. coli* and LPS can all increase CD14^{+}CD8^{+}T-cells when they are cultured with PBMC (**Fig.4d**), and to a much lesser extent when MNP are removed (**Extended Data Fig.5e**).

2) Related to 1, the metabolism phenotype is intriguing and might point to a mechanistic role of metabolic signals in driving the differentiation of this subset—can this be recapitulated in vitro (by feeding or starving these cells of various metabolites) to drive differentiation?

Yes, the role of the increased nutrient transporters we observed is an interesting possibility for future studies. We have not addressed this in our revised manuscript, focusing instead on the dual roles of stromal cell factors (**Fig.2**) and bacterial LPS (**Fig.4**) in driving $CD14^+CD8^+$T-cells.

3) The high levels of constitutive PD1 expression are suggestive of ongoing TCR stimulation. Is there any other evidence that TCR is being stimulated (transcriptional, functional) in these cells?

We have previously shown that PD-1 is highly expressed on global intrahepatic $CD8^+$T-cells and in particular on the tissue-resident T-cell fraction (*Pallett L.J. et al JEM 2017*). In fact, PD-1 was no higher on $CD14^+CD8^+$T-cells than other intrahepatic $CD8^+$T-cells (see below **Reviewer Fig.a**). $CD14^+CD8^+$T-cells did not show downregulation of CD3 typical of recently/chronically activated T-cells (**Reviewer Fig.b**). The high PD-1 expression on intrahepatic $CD8^+$T-cells generally, and $CD8^+T_{RM}$ in particular, is thought to be driven not only by antigen stimulation of TCR but also by prototypic liver cytokines such as TGFβ, shown to induce PD-1 in the cancer setting (*Park B.V. et al Cancer Discovery 2016*).

4) Figure 3g is a very nice experiment—elegant and informative.

We thank the reviewer for their appreciation of the HLA-mismatch experiments which we have now extended from a single one to five explanted liver allografts of different durations (ranging from eight months to 11 years; **Fig.1j**, **Extended Data Fig.1f,g**).

5) The repertoire data are interesting, but the presence of TCRs and tetramer positive cells in both CD14+ and CD14- subsets would suggest that antigen is not driving the differentiation alone.

Also, this complicates the interpretation of the PD1 expression—for the tet+ cells that are in both compartments, are the CD14- cells PD1 negative (suggestive that antigen is not around?)? This would help resolve the point in 3), whether PD1 is being driven by TCR signaling directly.

We agree that our data do not support a role for antigen stimulation through the TCR being the predominant factor driving $CD14^+CD8^+$T-cells *in vivo*. Instead we show that they are driven by stromal cell factors (**Fig.2**) and bacterial LPS (**Fig.4**), although they can then mount potent cytokine responses when stimulated through their TCR (**Fig.3f**). As mentioned above, PD-1 is ubiquitously expressed on liver-resident $CD8^+$T-cells and is not further increased on $CD14^+CD8^+$T-cells.

6) The repertoire analysis seems like it would be useful to apply to the LPS-expanded cultures to determine if LPS is non-specifically activating the entire CD14+ population or further selecting a specific subset.

We have instead further investigated how LPS expands $CD14^+CD8^+$T-cells in culture by quantifying cell division in a pre-labelled population (CFSE dilution of pre-stained $CD14^+CD8^+$T-cells) and the acquisition of new expression, (stained with an anti-CD14 monoclonal antibody labelled with a different fluorochrome added at the end of the 3 d *in vitro* culture period with UV-killed *E. coli*; **Extended Data Fig.5f,g**).

7) For the effector cytokine production, given the ease with which epitope-specific cells were identified (CMV, HBV), stimulation with cognate peptide would be preferable (and would potentially be more informative functionally) than the extraphysiological signal of CD3/CD28.

We agree that analysis of the functional response of virus-specific $CD8^+$T-cells would be more informative than that to anti-CD3/CD28. However, obtaining liver tissue from HLA-$A2^+$ donors with

detectable hepatitis B virus (HBV; **Fig.2h**) or cytomegalovirus (CMV; **Extended Data Fig.3f**) responses is not easy and their typical low-level effector responses to overnight peptide stimulation have proved difficult to detect above the high constitutive cytokine production of $CD14^+CD8^+$T-cells. A longer *in vitro* peptide stimulation protocol to expand their frequency would not be reflective of the *ex vivo* features of $CD14^+CD8^+$T-cells.

8) Figure 1c: This is difficult to read, there are too many data points on one graph. Jitter-ing the points, enlarging the graph, or choosing a different color scheme would be helpful.

It is difficult to visualise individual data points for the large number of liver samples we have studied (n=106) but jittering would not work for paired samples. Instead we have now used violin plots to better visualise the distribution of the data (**Fig.1e, i**).

9) Figure 5 and b need to also include absolute numbers of cells after culture. Including BrdU in the in vitro culture would also help show what proportion of the CD14+ cells expand.

Thank you for this good suggestion; we have now included CFSE labelling experiments showing that a small proportion of pre-existing $CD14^+CD8^+$T-cells are able to divide in culture in response to LPS whilst other $CD14^-CD8^+$T-cells start to express CD14 (**Extended Data Fig.5f,g**). We have also included $CD14^+CD8^+$T-cells as a percentage of total intrahepatic leukocytes ($CD45^+$) to better reflect absolute number changes (**Extended Data Fig.5d**).

10) It seems like more animals need to be included in 5j—the spread is too big and the power too low in its current form.

11) 5J through l (the mouse huPBL experiment) would also be improved by including a BrdU incorporation assay.

Following the sad death of our collaborator Dr Marcus Dorner we have removed these experiments, see *Overall Comments*. We have added Marcus's data from the better-established model of human immune system mice (NOD.$Rag1^{-/-}IL\text{-}2Rg^{-/-}$ engrafted with human foetal liver-derived $CD34^+$ stem cells) showing $CD14^+CD8^+$T-cells are enriched in barrier (lung, liver) compared to non-barrier organs (salivary gland, kidney) and that broad-spectrum antibiotics can decrease their frequency in the liver (**Fig.4i-k**). Otherwise, we have focused on experiments in humans.

12) Minor point: in extended data figure 3e, the enriched sequences in the TRBV7-8 BJ2-7 have a strong motif, mostly driven by the V and J germline sequences. The central T residue though is a rare occurrence in the 7-8/2-7 sequences in the vdjdb and may represent a non-templated addition. This would be easy to determine and would be an interesting side note further suggesting a strong signal of antigen-specific enrichment.

This is an excellent point. The middle 'T' likely originates from the TRD1 gene, however, the LAS sequence that is conserved among the expanded clones in this sample is non-template, which is further evidence suggesting this TCRβ chain sequence and the closely related clonally expanded sequences are the result of antigen-driven selection. We have now added this to the manuscript (**line 167**, **page 6**).

13) There's a generic comment about code and data availability that should be made specific. The sequencing data could be deposited and an accession number reported during the review process.

TCR repertoire and scRNAseq data has been deposited and will be available upon publication.

Referee #2 (Remarks to the Author):

This manuscript reports that human CD8+ T cells expressing CD14 and compartmentalized in the liver - and possibly in other organs exposed to bacterial products - have constitutive immunoregulatory features (expression e.g. of Foxp3, CTLA-4 and IL-10) but can rapidly mount effector responses (production of IFN-g, TNF) upon TCR stimulation. CD14+CD8+ T cells co-express TLR4, are rare in blood while are present in variable proportions in human liver samples from healthy tissues or in liver biopsies from patients with chronic HBV infection or other liver diseases, and can develop in HIS mice engrafted with human fetal liver-derived stem cells. Exposure to LPS or Gram-negative bacteria results in an increased proportion of CD8+CD14+ T cells, in vitro and in vivo, while administration of antibiotic against Gram-negative bacteria results in a reduced frequency of circulating and intrahepatic human CD8+CD14+ T cells in NGR mice. The experiments are of high technical quality and some of the findings are novel and exciting. However, there are limitations inherent to the experimental systems and hence difficulties in drawing conclusions on a mechanistic link between bacterial LPS, CD14+CD8+ T cells and the balance of hepatic tolerance versus immunity. The data provided raise a number of issues that could be addressed by the authors to strengthen the manuscript, as specified below.
We appreciate the recognition of technical quality and novelty.

Specific points:

1. A major issue is that the authors do not provide evidence that CD14/TLR4 is functional in liver CD8+ T cells. Other studies have shown that CD14 and TLR4 can be expressed on the surface of human CD8+ T cells and that these cells can directly respond to TLR4 agonists, while TLR4 knock down by siRNA attenuates this response (Komai-Koma et al., 2009).
We had referred to the *Komai-Koma M. et al* observation that human peripheral $CD8^{+}$T-cells differ from murine in their capacity to co-express $CD14^{+}$ and TLR4 after *in vitro* activation and have the capacity to react directly to LPS in a TLR4-dependent manner in combination with TCR stimulation. A further short report by *Zanin-Zhorov A. et al* showed human peripheral T-cells have the capacity to adhere to the extracellular matrix protein fibronectin in response to LPS, which fits nicely with our new findings for the stromal derivation of liver $CD14^{+}CD8^{+}$T-cells (**Fig.2a-f**) and our data that LPS upregulates CD49b (that binds fibronectin, **Extended Data Fig.5h**). We now present data to show that intrahepatic $CD14^{+}CD8^{+}$T-cells analysed directly *ex vivo* bind to LPS (**Fig.4a**), and preferentially divide in response to it as part of the response of mixed intrahepatic leukocytes (**Extended Data Fig.5c**). We could not obtain enough pure $CD14^{+}CD8^{+}$T-cells from human livers to look at function directly so instead used the $CD14^{+}CD8^{+}$T-cells we derived with stromal stimulation *in vitro*, having shown that these recapitulated the upregulation of TLR4 and constitutive immunoregulatory cytokine production characteristic of the liver-derived population (**Fig.2c** and **Extended Data Fig.5a**); after short-term UV-killed *E. coli* stimulation the functional profile of induced $CD14^{+}CD8^{+}$T-cells was distinct from that seen at rest or upon TCR stimulation, being instead differentially skewed towards the production of proinflammatory cytokines like IL-6 and chemokines like IL-8 and CCL5 (**Fig.4b**, **Extended Data Fig.5b**).

2. Another major issue is that most of the findings in the manuscript do not necessarily relate to a direct

effect of LPS on CD8+CD14+ T cells, given that experiments of LPS stimulation are not done on purified cells. In these conditions, it cannot be excluded that the effects are indirect and mediated by other LPS-sensing cells. If this was the case, it would still be an interesting, albeit different, report.
This points needs to be clarified, for example using the same in vivo model of adoptive transfer of human T cells into NRG mice and taking advantage of the CRISPR/Cas9 system to knock out CD14 gene, while simultaneously knocking in GFP gene as a reporter for CD14 promoter activity.

We have done further experiments with human samples to distinguish between direct and indirect effects of LPS on $CD14^{+}CD8^{+}$T-cells (see Q.1 and *Overall Comments*) and have better clarified this distinction in the manuscript. Although we have added some data suggesting direct sensing of LPS, the preferential responsiveness of $CD14^{+}CD8^{+}$T-cells to LPS *in vivo* will reflect both direct and indirect effects. Moreover, their distinct *ex vivo* profile when analysed directly out of the human liver is the most accurate reflection of the imprint left on them by their increased expression of CD14/TLR4.

3. Phenotypically, CD14+CD8+ liver T cells express Foxp3, CD25, CTLA-4, LAG3, which are characteristic of Treg cells but also of activated conventional human T cells. These findings raise questions as to the stability of the described phenotype.

This is a good point. Since some of the $CD14^{+}CD8^{+}$T-cells expressing these markers also produce IL-2 etc, they do not represent *bona fide* stable Foxp3-expressing TREG and may have upregulated some of these markers as a reflection of their highly activated state. However, the fact all constitutive IL-10 production by intrahepatic $CD8^{+}$T-cells segregates with the CD14-expressing fraction is functional evidence of their potential to mediate immunosuppression of surrounding cells; likewise their unusually potent production of IL-2 without stimulation identifies them as a source of this cytokine which is key for driving TREG expansion in neighbouring cells (**Fig.3e**). Whether they can also suppress surrounding responses by increasing local concentrations of adenosine through their co-expression of CD39/CD73 (**Fig.3d**), or through their expression of CTLA-4 (**Fig.3b**) remains unproven at this stage. Their capacity to switch to dominant production of INFγ, TNFα and MIP1β upon short-term TCR stimulation (**Fig.3f**) again support them being highly activated immunomodulatory cells, rather than stable TREG (**now clarified line 207-8, page 7**).

4. The authors need to clarify the clonal relatedness between CD14+ and CD14– CD8+ T cells, especially in light of the findings that these two populations share antigen specificity and TCR usage. The TCR sequencing data indeed support the notion that the two cell populations do not represent distinct lineages rather they may represent functionally distinct stages or different fates from a common precursor.

We agree with this interpretation of our findings on antigen specificity and TCR usage and have included additional data supporting the notion nicely summarised by this Reviewer that $CD14^{+}CD8^{+}$T-cells represent functionally distinct fates from a common precursor (**line 184, page 6**). Results supporting this interpretation have been extended in the new results sections to **Fig.2** (derivation) and **Fig.4** (bacterial LPS expansion) and summarised in *Overall Comments* above and the *Discussion* of the manuscript.

5. The data displayed in Fig. 3g of donor-derived a liver allograft explant are remarkable. However, it is difficult to conclude that CD14+ T cells are long lived, since one cannot exclude the possibility that these cells are continually generated from a CD14– population. There is also a different proportion of CD14+ cells of donor or recipient origin. This finding needs to be commented.

The data this reviewer remarked upon from one liver allograft have now been extended to five, showing a consistent striking enrichment of CD14$^+$CD8$^+$T-cells in the small donor fraction (**Fig.1j**, **Extended Data Fig.1f,g**). We have modified our interpretation to refer to their preferential accumulation within the small long-lived donor fraction which could represent either long-lived cells or more likely turnover of their progeny continuing to express ± acquire CD14 (**lines 114, 118, page 4)**. Their particularly striking enrichment in the mismatched allografts removed at the earliest timepoints suggests they may be better suited to retention being less susceptible to pre-transplant perfusion removal than other donor CD8$^+$T-cells, in line with their stromal homing profile. The increased expression of CD14 on donor-derived CD8$^+$T-cells, especially at earlier time points post-transplant may also reflect increased homeostatic turnover to replenish the massively depleted donor pool (see suggestion from Reviewer 4); this is supported by the increased Ki67 on CD14$^+$CD8$^+$T-cells (**Fig.1h**) and our new finding that CD14 is preferentially expressed on the most actively dividing cells following LPS stimulation (by CFSE dilution, **Extended Data Fig.5f,g**).

Now that we have five allografts, this Reviewer's observation about the recipient liver-infiltrating CD8$^+$T-cells has become more obvious, since they clearly express less CD14 (**Fig.1j**) than CD8$^+$T-cells in non-transplanted livers **(Fig.1d,e)** as well as in the donor fraction, (**Fig.1j**). Whilst recipient liver-infiltrating CD8$^+$T-cells infiltrating the liver can acquire some T$_{RM}$ markers (CD69$^{+/-}$CD103), they have less CXCR3 than the donor-derived T$_{RM}$ (*Pallett L.J., Burton A.R. et al JEM 2020*) consistent with an inability to migrate into the liver parenchyma for the stromal interactions that we now show promote CD14 induction. This is now described **lines 115-121 and 124-127, page 4**.

Referee #3 (Remarks to the Author):

The authors describe a CD8 T cell subset found in liver that displays both immunoregulatory and proinflammatory characteristics by expressing a Treg signature (i.e., FoxP3, CTLA4, IL10 and IL2) and effector cytokines (IFNg and TNFa). TCR repertoire analysis indicates that these cells are differentiated from common progenitors with CD14– CD8 T cells or CD14– liver resident CD8 T cells and may obtain CD14 expression upon particular stimuli. These cells can be expanded by gram-negative bacteria and LPS, suggesting the functional significance of CD14 expression by these tissue-resident CD8 T cells. While identification and characterization of tissue-resident CD8 T cells that potentially possess a specialized function may be interesting, this study lacks novelty because the majority of the data presented here have been described in previous studies by these and other authors (Pallett LJ JEM 2017, Komai-Koma M EJI 2009). Furthermore, these studies lack data that show the functional

attributes of these CD8 T cells in the tissue environment. A potentially new and relevant aspect of this study would have been the immunoregulatory capacity of this CD8 T cell subset during pathogen invasion to preserve tissue homeostasis. However, the authors did not explore this critical feature experimentally and only speculate on their functionality according to expression of surface molecules and cytokine production. Overall, while this study has investigated the phenotype of liver-resident CD14+ CD8 T cells in more detail compared to previous reports, neither their contribution to tissue homeostasis nor their contribution to liver inflammation have been investigated, which limits the impact of this study in understanding these tissue resident CD8 T cells.
Regarding this Reviewer's comments on overlapping novelty:

In *Pallett L.J. et al JEM 2017* we simply observed the presence of CD14 expression on a fraction of CD69/CD103 expressing $CD8^+$T-cells within the human liver. All the other features we described in that paper were characterised on global $CD8^+T_{RM}$; for example, we noted increased IL-2 production by liver $CD8^+T_{RM}$ after short-term TCR stimulation, whereas in this study we find that most of this induced, and all the constitutive unstimulated, IL-2 originates from the fraction expressing CD14.

In *Komai-Koma M. et al EJI 2009* CD14/TLR4 were observed to be upregulated on a fraction of $CD8^+$T-cells upon *in vitro* stimulation of PBMC rather than *ex vivo* on tissue-compartmentalised T-cells as we have shown. LPS was shown to adjuvant the response to TCR stimulation on an *in vitro* stimulated population whereas we profile their unique phenotypic, TCR/antigen specificities and functional properties directly from human liver as well as determining their stromal induction and showing their capacity for *in vivo* expansion/contraction according to bacterial LPS load.

Specific comments:

1. The authors suggest the immunomodulatory potential of CD14+CD8+ T cells based on their expression of FOXP3, CD25, CTLA4, LAG3 etc (Fig. 2). Although these molecules belong to the CD4 Treg signature, expression by a subset of T cells also reflects the state of activation. To claim that they are regulatory CD8 T cells in the liver tissue environment, their suppressive activity should be demonstrated.
Please see response to Reviewer 2, point 3.

2. The authors state that CD14+CD8 T cells express proinflammatory effectors IFNg, TNFa and MIP1b upon TCR stimulation while maintaining IL10 and IL2. From the data in Fig. 2c, it is clear that the IL10 producing CD14+CD8+ T cells co-express IL2. However, the tSNE plots in Fig. 2f show that IFNg and TNFa producing cells belong to a very minor fraction of IL10/IL2 producers, which do not support their statement that the CD14+CD8+ T cells function as both regulatory and effector T cells. The data shown do not provide convincing evidence that these opposite roles are exerted by this liver resident CD14+CD8 T cell subset depending on distinct immune conditions (tissue homeostasis vs, pathogenic challenge).
We did show a distinct population of $CD14^+CD8^+$T-cells able to co-produce IL-10/IL-2/IFNγ (mean 14%, **Fig.3g** and by tSNE **Extended Data Fig.4b**). Although this co-production is an unusual feature of interest, an important consideration is that within the overall fraction of intrahepatic $CD8^+$T-cells expressing CD14 there are some that exert constitutive IL-10/IL-2 production (**Fig.3e**), some that respond within 4 hr of TCR stimulation to produce more IFNγ/TNFα/MIP1β than their $CD14^-CD8^+$T-cells (**Fig.3f**) and some that may respond with a different functional profile to LPS stimulation (according to the new data from the stromal cell-induced $CD14^+CD8^+$T-cells; **Fig.4b**, **Extended Data Fig.5b**).

3. Fig. 3 is devoted to another set of characterization of surface marker expression by CD14+CD8+ T cells, some of which was described in their previous report (JEM, 2017).
While the majority of markers expressed by CD14+CD8 T cells that are associated with activation and cytotoxic activity are expressed at similar levels as CD14– CD8 T cells, some markers that are upregulated by CD14+CD8 T cells, including CD71, CD98 and CD36, indicate they are metabolically active. While this selective marker expression pattern may reflect a specialized function of this cell

type, this study does not go beyond phenotype analysis and it is therefore not clear whether the acquisition of this phenotype by CD14+CD8 T cells is critical for their contribution to the immune response in liver tissue.

The surface markers we have analysed on $CD14^+CD8^+$T-cells inevitably overlap with those analysed in our previous study on liver $CD8^+T_{RM}$ since these are many of the most important available panels to analyse T-cells by flow cytometry. However, there is no replication of data since in *Pallett L.J. et al JEM 2017* we had not analysed any markers within the $CD14^+$ versus $CD14^-CD8^+$T-cells and had not included many markers studied here (e.g. TLR2, TLR4, MD-2, CD36, CD71, CXCR4, CD49a, CD49b, Pan-KIR, CD38, CD25, Foxp3, CTLA-4, CD73, LAG-3, GITR, ICOS, NKG2A).

We have now pointed out that CD36, in addition to its metabolic roles, functions as a co-receptor with TLR2/TLR6 or TLR4/TLR6 heterodimers so that its striking enrichment on $CD14^+CD8^+$T-cells merits future investigation in the context of a potential role as a DAMP for recognising oxidised low-density lipoproteins (**lines 87-92, page 3**). We have also clarified that expression of the other nutrient transporters studied (CD98 and CD71; **Extended Data Fig.4a**) may simply reflect the highly activated state of these cells (**lines 195-197, page 6-7**); we have not addressed any potential specific metabolic re-programming of $CD14^+CD8^+$T-cells in this study.

4. Fig. 3g: the authors intended to examine the longevity of CD14+CD8 T cells using liver allograft obtained from an HLA-mismatched recipient. The FACS profile presented, however, indicates that the authors failed to detect donor derived HLA-A2+ cells, in contrast to the statement in the ms. In general, HLA-A2 Abs can detect a clear population if they exist. On the other hand, even if successful, this experiment would not lead to the conclusion that CD14+CD8 T cells are long-lived. There is also no evidence that CD14+ CD8 T cells are stable and this fraction could potentially have been differentiated from progenitors after transplantation. Therefore, this experiment is not appropriate to test the longevity of CD14+ CD8 T cells.

If any, the data in Fig. 3g is also not consistent with data shown in the previous figures: ~15% of endogenous intrahepatic CD8 T cells in recipients should express CD14, if their basic analysis shown in Fig. 1a is representative. Here, the authors show that CD14+ CD8 T cells are found only in the donor-derived fraction.

We hope that the Reviewer is more convinced by the new donor-HLA stains we have included in the revised manuscript (**Fig.1j, Extended Data Fig.1f**); HLA-specific mAb do give less distinct staining on intrahepatic leukocytes than we typically see in the periphery but we and others have recently been able to use them to distinguish donor and recipient $CD8^+$T-cells within MHC-mismatched allografts in other studies (*Pallett L.J. Burton A.R. et al JEM 2020, Cuff A.O. et al JI 2016*). See also answer to Reviewer 2, point 5.

5. Fig. 5: to analyze the physiological relevance of CD14 expression by tissue resident CD8 T cells, the expansion of CD14+CD8 T cells after stimulation with LPS was analyzed in vitro using bulk intrahepatic leukocytes. Although the binding of LPS to CD14 on CD8 T cells may have stimulated their expansion, this effect could also be due to increased activation of APCs. Earlier studies by Komai-Koma showed that purified CD8 T cells can be stimulated with LPS to induce perforin and granzyme. Therefore, in principle, purified CD14+CD8 T cells can directly respond to LPS or gram-negative bacteria. However, the current data may include both direct and indirect effects of LPS in the presence of potentially CD14hi non-CD8 cells.

Thank you for this important point. Please see responses to Reviewer 2 point 1 and 2 and *Overall Comments*.

6. Fig. 5k: In the analysis of CD8 T cells recovered from NRG mice reconstituted with human CD3+ T cells, the authors show that LPS stimulation upregulates IL10 and IL2 production while almost all CD14+ CD8 T cells produce IFNg. It is not clear why CD14+ CD8 T cells express this high level of IFNg in this adoptive host.
In this figure, cytokine (IL10, IL2, IFNg) production is depicted by percentage while expression of FoxP3 is presented by cell numbers. Is there a reason for this inconsistency; it would be more useful to show the percentage for FoxP3.
In this analysis, CD8 T cells express both immunomodulatory and effector molecules. What is the functional significance of this dual phenotype for tissue immune homeostasis in this engraftment model?
We have removed data from this potentially flawed model, please see response to Reviewer 1 points 10 -11 and *Overall Comments*.

Referee #4 (Remarks to the Author):

The current study by Pallett et al. describes a new subset of CD8+ T cells in the liver and proposes a contribution of these cells to the immunomodulatory state of the liver. The immunomodulatory function of the liver is an important topic with respect to the body's physiology and within clinical settings like organ transplantation, chronic infection and cancer. So far, the underlying mechanisms are poorly understood. Using unique human sample materials complemented by experiments in humanized mice, Pallett et al. were able to provide an interesting T cell subset, CD14+ CD8+ T cells, within the complex puzzle of modulators of the immune response within the liver. However, the overall concept behind the described CD14+ CD8+ T-cell population within the liver is weakly defined and thus somewhat preliminary and needs further clarification. Specific points are listed below:

- Do CD8+ CD14+ T cells express CD8a/a or CD8a/b?
Data from our scRNAseq indicates $CD14^{+}CD8^{+}$T-cells express CD8αβ (**Reviewer Fig.c**)

- Is CD14+ expression specific for CD8+ T cells in the liver? Or do e.g. CD4+ T cells or NK cells also express CD14?
CD14 expression is detectable at a lower frequency on $CD4^{+}$T-cells and $CD56^{+}CD3^{-}$ NK cells than on $CD8^{+}$T-cells within the human liver (**Reviewer Fig.d**).

- The authors provide mRNA data of CD14, TLR4 and MD2 to confirm intrinsic expression. However, relation to mRNA expression levels in CD14+ myeloid cells are missing.
We have now included this comparison by RT-PCR and scRNAseq. As expected MNP express considerably higher levels of CD14 transcripts (**Extended Data Fig.2b,d**). Transcripts of TLR4, MD-2 are also increased in MNP whereas CD3ζ is present at equivalent levels in $CD14^{+}$ and $CD14^{-}CD8^{+}$T-cells and absent on MNP (**Reviewer Fig.e**).

- CD14+ CD8+ T cells seem to consist of different subpopulations given the bimodal expression of multiple surface markers and that some cells are virus-specific. What does this mean in terms of the

differentiation/activation status of these cells? Are there any specific co-expressions of distinct markers found (e.g. in t-SNE analyses)?
Along this line, what is about the plasticity/the dynamics of the immunomodulatory CD14+ CD8+ T cells? Some epigenetic analyses and if possible an exemplified longitudinal analyses would provide further insights into these questions. In addition, epigenetic analyses in comparison to CD14-CD8+ T cells and CD14+ myeloid cells would provide insights into the relationship of CD14+CD8+ T cells to both cell types.

We agree that $CD14^+CD8^+$T-cells are not a homogenous lineage but rather a fraction of liver-compartmentalised $CD8^+$T-cells enriched to varying degrees for a number of features reflecting interactions with stroma and LPS (**line 183-6, page 6**). We were not able to segregate them into distinct subsets by scRNAseq using the SMART-Seq2 platform (**Extended data Fig.2d**) and have not been able to isolate sufficient of these cells from human liver to carry out epigenetic analyses. Because of their tissue localisation, longitudinal analysis has not been possible, with the exception of the newly added re-sampling of $CD14^+CD8^+$T-cells from the ascitic tap of a patient with spontaneous bacterial peritonitis before and after antibiotic treatment (**Fig.4h**).

- CD14+CD8+ T cells are present in the liver and in the skin. Does this mean that these cells are tissue-specific subpopulation and cannot be found in the circulation? Are these cells a subset of tissue-resident T cells? However, at least not all are tissue-resident and therefore CD14 is not a unique feature of tissue-resident CD8+ T cells. Does this mean that the CD14+ CD8+ T cells are replenished from the circulation by CD14- CD8+ T cells? Please comment on that.

Yes, $CD14^+CD8^+$T-cells are virtually absent from the circulation and detectable within the liver and the skin, and as we now show, the spleen, lymph nodes and peritoneal cavity in humans (**Fig.1e, Fig.4g**) and spleen, liver and lungs (but not the non-barrier salivary gland or kidney) in humanised immune system mice (**Fig.4i**, see below). This compartmentalisation, together with their persistence in the liver and inability to exit into the periphery from the donor pool allografts, confirms them to be tissue-sequestered. Moreover, they are enriched for T_{RM} phenotypic markers; we now show they constitute the main CD49a-expressing cells in the liver (**Fig.2b**), a marker which has been found to identify T_{RM} in skin and lungs able to interact with epithelium. As the Reviewer rightly points out, CD14 expression is not a feature of all liver-resident $CD8^+$T-cells and we now show that this is likely a reflection of their encounters with stromal and LPS signals +/- their proliferative status. Our additional data from a total of five MHC-mismatched allografts indicates that, at least in that setting, $CD14^+CD8^+$T-cells cannot be replenished from the periphery (**Fig.1j, Extended Data Fig.1f,g**) but likely only from the pool of $CD8^+$T-cells tethered within the parenchyma by stromal cells.

Along this line, given that CD14+CD8+ T cells are proliferative and long-lived, is there a difference in Ki67 and BCL-2 expression between CD103+CD69+ and CD103- CD69+ CD14+ CD8+ T cells? This may reflect distinct differentiation/maturation sates of the CD14+ CD8+ T cells.

Ki67 is increased on the fraction of intrahepatic $CD8^+$T-cells expressing CD14 (mean 9%, reaching as high as 45%, **Fig.1h**). Consistent with chronic activation, $CD14^+CD8^+$T-cells are also markedly enriched for CD57 expression (**Fig.1h**). Both these features contrast with findings in our previous study in the liver and those in other organs (*Pallett L.J. et al JEM 2017, Kumar B.V. et al Cell Reports 2017)* that have found that classical $CD69^+CD103^+$ T_{RM} have low Ki67 and CD57. These findings suggest CD14 expression may be a feature of proliferating $CD8^+$T-cells with the requisite cellular and LPS exposure within the liver stroma. Bcl-2 and Bim expression did not differ in $CD14^+CD8^+$T-cells

but we have not carried out an extensive analysis of their pro/anti-apoptotic propensity (**Reviewer Fig.f**).

- Fig. 3g: A high proportion among the donor-derived cells are CD14+ CD8+ T cells. This implies that a high proportion of tissue-resident CD8+ T cells are CD14+ and thus immunodmodulatory in the liver? What is the fraction of tissue-resident CD8+ T cells in the liver that express CD14? Is the TCR repertoire similar distributed among tissue-resident and CD14+ CD8+ T cells or are CD14- and CD14+ tissue-resident CD8+ T cells comprised of distinct clones?

See above, we agree that CD14 expression is clearly a feature of tissue-resident $CD14^+CD8^+$T-cells. T_{RM} can be identified by a number of different phenotypic definitions so the proportion expressing CD14 will differ depending on definition (e.g. single expression of CD69, double expression CD69/CD103, expression CD49a). We now show that $CD14^+CD8^+$T-cells are enriched for all these markers compared to $CD14^-CD8^+$T-cells (**Fig.2b, Extended Data Fig.2a**). Our TCR sequencing identified a repertoire of similar clonal diversity and some overlapping TCRs between the $CD14^-$ and $CD14^+$ fraction of intrahepatic $CD8^+$T-cells (**Fig.2g, Extended Data Fig.3a-c**); most of these would represent T_{RM} by the loosest definition but we did not formally analyse the repertoire within $CD14^+$ and T_{RM} subsets simultaneously.

- Do the authors have any ideas about the triggers inducing CD14+ CD8+ T cells? According to the HIS model, is homeostatic proliferation one factor? Performing a similar transfer of murine liver-derived stem cells into NRG mice would further clarify this point.

Thank you for the excellent suggestion re. homeostatic proliferation driving CD14 expression (**added line 105, page 4).** Although we have not performed the suggested murine experiment, we think this concept fits with several of our findings: the increase in CD14 expression within the markedly depleted donor allograft pool especially in the earlier samples post-transplant (**Fig.1j**); the very high Ki67 on $CD14^+CD8^+$T-cells (**Fig.1h**) and the acquisition of new CD14 expression on the CFSE diluted fraction of dividing cells following LPS stimulation (**Extended Data Fig.5f,g**). We have also now shown that stromal cell factors (extracellular matrix proteins and CXCL12, **Fig.2c-e**) can promote the induction of $CD14^+CD8^+$T-cells (see *Overall Comments*).

Furthermore, please at least discuss whether there is an impact of the SPF condition or specific microbiota (not load) on the induction of CD14+ CD8+ T cells. The increased fraction of CD14+ CD8+ T cells in E.coli cultures does not provide evidence for intrinsic induction or expansion of CD14+ CD8+ T cells since CD14 could be taken up from CD14+ cells within leukocytes. Thus, CD14 transcript levels should also be assessed in CD14+ CD8+ T cells after E.coli stimulation.

The impact of specific bacterial composition (in addition to LPS overall load) would be a very interesting factor to consider in future studies since different LPS structures in different gram-negative bacteria affect their ability to bind TLR4 as we mention in our *Discussion* (**line 328-330, page 10-11).** The capacity of gram-positive bacteria to interact with $CD14^+CD8^+$T-cells through their expression of TLR2 also merits study. The possibility of CD14 being acquired from neighbouring cells as well as/instead of T-cell-intrinsic expression is an important point that we have now addressed (see *Overall Comments* and responses to Reviewer 1 point 6). Our new data suggest that most CD14 on $CD8^+$T-cells is not cell-intrinsic but acquired (from activated MNP or other CD14-expressing cells such as hepatocytes) by activated/proliferating T-cells interacting with stromal cells Although LPS can drive proliferation and function of existing $CD14^+CD8^+$T-cells, our data suggest it probably drives their

expansion predominantly by activating MNP (directly or via stromal cells) to transfer CD14 (**Extended Data Fig.5e-g,i**).

In the skin blister model, absolute cell counts of CD14-/CD14+ CD8+ T cells as well as assessing CD14 mRNA levels will further clarify whether CD8+ T cells differentiate into CD14+ CD8+ T cells or are recruited or expand. The applied experimental systems do also not address the question whether LPS or other E.coli-derived PAMPs directly affect CD14+CD8+ T cells or whether this are indirect effects mediated by accessory cells. Please elaborate this further, e.g. by directly stimulating or co-stimulating sorted CD14- vs. CD14+ CD8+ T cells with LPS/E.coli-derived PAMPs and testing proliferation and effector functions. This would also provide further insights into the role of CD14 in the context of TCR stimulation.

We have now added the percentage of $CD14^{+}CD8^{+}$T-cells as a proportion of all leukocytes ($CD45^{+}$) in the skin blisters (**Extended Data Fig.6b**). We would not be able to obtain sufficient cells from the skin blister model to purify for RT-PCR. For direct versus indirect LPS effects, please see responses above.

- To name CD14+ CD8+ T cells "innate-like" seems to be misleading given that some of the cells are virus-specific. Is there evidence for any "master regulator" of innate-like T cells that is also expressed by CD14+ CD8+ T cells, e.g. PLZF?

We have removed the term innate-like which we used to refer to their capacity to respond to a bacterial PAMP but agree could be misleading. CD14+CD8+ T cells are not "innate-like" in the sense that they do not have increased PLZF or NKG2A although they do have an increase in KIR expression (**Extended Data Fig.1e**).

Based on the TCR repertoire analyses the authors should extend the detection of semi-invariant T cells beyond MAIT cells. To what extent are semi-invariant T cells present in CD14+ compared to CD14- CD8+ T cells?

We have now analysed the presence of semi-invariant T-cells more thoroughly in our TCR analysis of $CD14^{+}CD8^{+}$T-cells, showing very few invariant-NK-T-cells, germline-encoded mycolyl reactive or mucosal-associated invariant T-cells (**Extended Data Fig.3d**). Please note that this is likely a reflection of our gating strategy that excluded $CD56^{+}$ cells from our sorts and all flow cytometry so that we could concentrate our analysis on conventional T-cells.

- It remains unclear whether the presence of CD14+ CD8+ T cells is specific for the liver and thus provide a unique immunomodulatory rheostat within the gut-liver axis. As shown by the authors these cells can also be found in the skin blister model clearly questioning their restriction to the liver. Analyzing diverse organs of HIS mice will further clarify whether CD14+CD8+ T cells are also present in other barrier tissues like the gut, skin, lungs or organs like the kidneys and salivary glands or even lymphoid tissue like the spleen and lymph nodes.

Thank you for this suggestion. We have now shown that $CD14^{+}CD8^{+}$T-cells can also be detected in human spleen and lymph nodes as well as liver and skin (**Fig.1e**). In addition, they accumulate at particularly high frequencies in the peritoneal cavity in the setting of cirrhotic ascites (**Fig.4g**). In the humanised immune system mice we now show that $CD14^{+}CD8^{+}$T-cells are significantly enriched in the spleen and lung as well as liver, whereas they are at similar negligible levels in the non-barrier organs kidney and salivary glands to blood (**Fig.4i**). These data suggest $CD14^{+}CD8^{+}$T-cells are a

feature of lymphoid organs and of non-lymphoid barrier organs (the liver can be considered to be a semi-barrier organ because of its high exposure to bacterial products through the portal vein). Future studies should address the presence and roles of $CD14^+CD8^+$T-cells in other human barrier organs such as the lungs.

Reviewer Figure

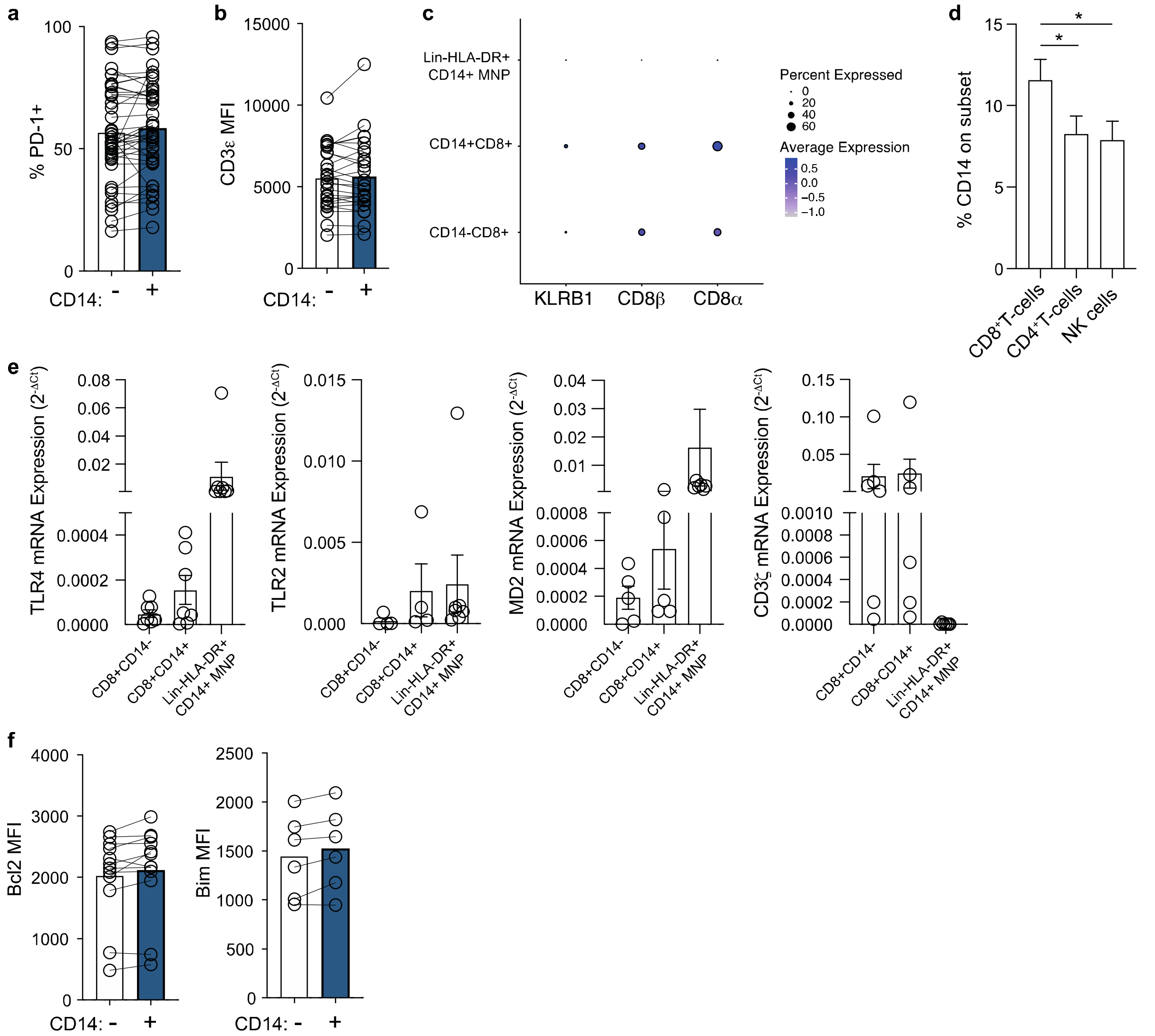

Summary data showing mean fluorescence intensity (MFI) or percentage expression of **a** PD-1 (n=43) and **b** CD3ε (n=26) on intrahepatic CD14-CD8+T-cells (black outline) or CD14+CD8+T-cells (blue filled). **c** Dot plot depicts the percentage expressing and relative expression levels of CD8α, CD8β and KLRB1 (CD161, a marker enriched on CD8αα T cells) determined by single-cell RNA-Seq transcriptome analysis of FACS-sorted intrahepatic CD14-CD8+T-cells, CD14+CD8+T-cells or Lin-HLA-DR+CD3-CD14+ MNP from two separate liver samples. **d** Assessment of CD14 expression on CD8+T-cells, CD4+T-cells and CD3-CD56+ NK cells. **e** Assessment of TLR4, TLR2, MD2 and CD3ζ mRNA by rt-PCR (relative to an internal 18S control) in FACS-sorted Lin-HLA-DR+CD3-CD14+ mononuclear phagocytes (MNP), intrahepatic CD14-CD8+T-cells and CD14+CD8+T-cells. **f** Summary data showing mean fluorescence intensity (MFI) Bcl2 (n=12) and Bim (n=6) on intrahepatic CD14-CD8+T-cells or CD14+CD8+T-cells.

Reviewer Reports on the First Revision:

Referees' comments:

Referee #1 (Remarks to the Author):

The manuscript has been substantially re-organized and re-focused, directly addressing the question of ontogeny more thoroughly than the prior version and clarifying some functional issues as well. Most of my major concerns have been addressed, but the new data raise some additional questions.
1) The levels of TLR4, MD-2, and TLR2 transcriptionally are substantially lower than in MNPs. This reviewer figure should potentially be included in the extended figures in the main document. Given this, can the authors speculate on whether these elements of the complex are also captured by trogocytosis or do they think this level of expression is high enough to confer a functional response?
2) Can the authors provide a negative control image for Figure 1b, perhaps a CD14- negative cell so that we can see what negative levels of CD14 and TLR4 are by this assay?
3) Although it may not be relevant, can the authors also report on whether there is a difference in caspase-11 message based on their scRNA-seq data between the CD14+ and CD14- populations? I realize caspase-11 is only relevant for cytoplasmic LPS, but it might still be informative.
4) Can the authors clarify the numbers of individuals the repertoire analysis in ext figure 3 comes from? At the top of the legend it says four donors. In figure cthere is a blow up of TRBJ usage for enriched TRBVs in “one or both liver samples”—I think this means in the CD14+ or CD14- from one donor, but while the 11-2 enrichment is more even, the 7-8 enrichment looks enriched in “both” samples. In the chart showing the ranked frequency of the conserved CDR3 motif, can the motif frequency be listed in two columns, corresponding to the CD14+ and CD14- contributions?
5) Very minor point: in line 127 the authors refer to the CD8 Trm “residing within the vasculature”—is it accepted to refer to vascular T cells as “Trm”? Wouldn’t these be accessible to blood-based antibody staining (which I thought was the gold standard for ‘non-residency’?)

UW: The reviewer notes in comments to the editor that he/she remains particulalrly concerned about the expression of the entire surface LPS-receptor complex--as none of the components are expressed at reasonable levels at the mRNA level.

Referee #2 (Remarks to the Author):

The authors have carefully considered the points raised by the reviewers. Overall, the manuscript reports novel and exciting findings.

Referee #3 (Remarks to the Author):

Identification of a T cell subset that adapts to the liver tissue microenvironment and contributes to this organ’s immune homeostasis may provide new insight into tissue-specific immune regulation. The authors have analyzed CD8 T cells that may exert immunomodulatory activity in response to

environmental stimuli. However, the revised ms does not include data that clarifies the functional significance of these cells and does not address comments raised previously.

Comment 1. We had asked whether CD14+ CD8 T cells might exert immune regulatory activity based on observations contained in an earlier version of this ms. While the authors speculate that these CD8 T cells suppress surrounding cells by IL-10 production and CD39/73 expression, additional experimental data supporting this idea have not been provided.

Comment 2. We requested functional data on CD14+ CD8 T cells that coexpress IFNg, TNFa, IL-10 and IL-2. Although the authors now note that a fraction of CD14+ CD8 cells constitutively express IL-10/IL-2 and some express IFNg/TNFa/MIP1b, the potential functional relevance of IL-10/IL-2 production (or other cytokines produced) under different immunological conditions is lacking.

Comment 3. We had asked whether expression of CD71, CD98 and CD36 by CD14+ CD8 cells indicated a metabolically-active state that might separate this CD8+ subset from CD14– CD8 T cells. The authors have not provided information on this point. Instead, they list additional markers of CD14+ CD8 T cells compared with their earlier study (JEM, 2017).

Comment 4. We had questioned the conclusion that CD14+ CD8 cells are long-lived after liver allograft transplantation. While the authors now provide a better FACS plot for detection of donor cells, questions of lifespan and renewal have not been addressed. The authors now state that CD14+ CD8 T cells acquire CD14 protein from other surrounding cells, including mononuclear phagocytes, based on scRNA-Seq and co-culture experiments. It is surprising that donor CD8 cells (and not endogenous CD14+ CD8 T cells) specifically acquire CD14 from surrounding phagocytes and maintain this protein for long periods of time.

Comment 6. We had asked about the functional relevance of CD14+ CD8 T cells that express both IFNg and IL-10 in the liver tissue environment. Although the authors refer to a response to another reviewer, it does not address this question.

Referee #4 (Remarks to the Author):

In the revised manuscript, Pallett et al. have elaborated in more detail on the ontogeny and function of CD14+CD8+ T cells compared to the initial version of the manuscript. The authors present a very comprehensive study, have applied challenging and cutting-edge methods and have investigated unique patient samples to characterize this activated tissue-sequestered novel CD8+ T cell population. Still, the question about the biological relevance of CD14+CD8+ T cells remains elusive.

Nevertheless, this is an elegant study providing some novel insights into the immunity at barrier tissues.

I have two minor remarks:

(1) since CD14+CD8+ T cells are not restricted to the liver (as nicely shown by the authors), a greater emphasize should be made on the discussion of the potential role of CD14+CD8+ T cells in barrier tissues in general;

(2) to clarify that CD14 expression is not restricted to CD8+ T cells within the lymphocyte compartment, the data on CD14-expressing CD4+ T cells and NK cells (as provided for the reviewer) should be included in the manuscript.

Author Rebuttals to First Revision:

Point-by point to Referees' comments:

Referee #1 (Remarks to the Author):

The manuscript has been substantially re-organized and re-focused, directly addressing the question of ontogeny more thoroughly than the prior version and clarifying some functional issues as well. Most of my major concerns have been addressed, but the new data raise some additional questions.
Thank you for acknowledging all our additional work on ontogeny and function of these cells.

1) The levels of TLR4, MD-2, and TLR2 transcriptionally are substantially lower than in MNPs. This reviewer figure should potentially be included in the extended figures in the main document. Given this, can the authors speculate on whether these elements of the complex are also captured by trogocytosis or do they think this level of expression is high enough to confer a functional response?
We have now included the figure showing that transcriptional levels of TLR4, MD-2 and TLR2 are very low in $CD14^+CD8^+$T-cells. Instead we have gone on to demonstrate that they can acquire not only CD14, but also these other components of the LPS receptor from myeloid cells. We show that levels of the receptor on *ex vivo* liver, and *in vitro* induced, $CD14^+CD8^+$T-cells are sufficient for LPS binding. Their differential cytokine response to LPS is partially direct since it is maintained with purified $CD14^+CD8^+$T-cells *in vitro*. However in tissues, their response to LPS is likely also indirect, via activation of interacting MNP.

2) Can the authors provide a negative control image for Figure 1b, perhaps a CD14- negative cell so that we can see what negative levels of CD14 and TLR4 are by this assay?
We have included new image stream data showing $CD8^+$T-cells that are negative for CD14 (Fig.2l).

3) Although it may not be relevant, can the authors also report on whether there is a difference in caspase-11 message based on their scRNA-seq data between the CD14+ and CD14- populations? I realize caspase-11 is only relevant for cytoplasmic LPS, but it might still be informative.
We have re-analysed our scRNA-Seq data looking for caspase-11 and other transcripts potentially reflective of LPS downstream signalling. We only found enrichment of one relevant transcript (*TRAM1*), not caspase 11, but isolated $CD14^+CD8^+$T-cells had not been exposed to LPS before RNA extraction, so were not optimal for these analyses.

4) Can the authors clarify the numbers of individuals the repertoire analysis in ext figure 3 comes from? At the top of the legend it says four donors. In figure cthere is a blow up of TRBJ usage for enriched TRBVs in "one or both liver samples"—I think this means in the CD14+ or CD14- from one donor, but while the 11-2 enrichment is more even, the 7-8 enrichment looks enriched in "both" samples. In the chart showing the ranked frequency of the conserved CDR3 motif, can the motif frequency be listed in two columns, corresponding to the CD14+ and CD14- contributions?
This point could have been addressed, however in the current version of the manuscript we have removed all TCR repertoire analysis to make room for new data. We felt the TCR data were taking a lot of space and distracting form the main flow and would be better published separately as part of ongoing work on the TCR repertoire in antigen-specific and global intrahepatic T cells.

5) Very minor point: in line 127 the authors refer to the CD8 Trm "residing within the vasculature"—is it accepted to refer to vascular T cells as "Trm"? Wouldn't these be accessible to blood-based antibody staining (which I thought was the gold standard for 'non-residency'?)
The liver vasculature is unusual, being lined by specialised sinusoidal endothelial cells that lack a basement membrane and have fenestra allowing intravascular T-cells to contact parenchymal and stromal cells through the endothelium. In the liver, $CD8^{+}T_{RM}$ do, in fact, largely reside within the vasculature; although they label with intravascular antibodies (usually used to exclude residency) they have been confirmed to be *bona fide* $CD8^{+}T_{RM}$ by parabiosis experiments (*Steinert, Cell 2015, Fernandez-Ruiz, Immunity 2016*).

UW: The reviewer notes in comments to the editor that he/she remains particulalrly concerned about the expression of the entire surface LPS-receptor complex--as none of the components are expressed at reasonable levels at the mRNA level.
As above, we have now shown that the key components of the LPS-receptor complex can be acquired from MNP in an actin/ICAM-1-dependent manner. Moreover, our data now suggest that the interaction of $CD14^{+}CD8^{+}$T-cells with myeloid cells results in comprehensive reprogramming beyond the acquisition of direct T-cell responsiveness to LPS, i.e that CD14 also marks out $CD8^{+}$T-cells that have been instructed by myeloid cells.

Referee #2 (Remarks to the Author):

The authors have carefully considered the points raised by the reviewers. Overall, the manuscript reports novel and exciting findings.
Thank you for this assessment.

Referee #3 (Remarks to the Author):

Identification of a T cell subset that adapts to the liver tissue microenvironment and contributes to this organ's immune homeostasis may provide new insight into tissue-specific immune regulation. The authors have analyzed CD8 T cells that may exert immunomodulatory activity in response to environmental stimuli. However, the revised ms does not include data that clarifies the functional significance of these cells and does not address comments raised previously.

Comment 1. We had asked whether CD14+ CD8 T cells might exert immune regulatory activity based on observations contained in an earlier version of this ms. While the authors speculate that these CD8 T cells suppress surrounding cells by IL-10 production and CD39/73 expression, additional experimental data supporting this idea have not been provided.
This is beyond the scope of the current manuscript and we have therefore removed speculation about an immunosuppressive role of $CD14^{+}CD8^{+}$T-cells on surrounding cells.

Comment 2. We requested functional data on CD14+ CD8 T cells that coexpress IFNg, TNFa, IL-10 and IL-2. Although the authors now note that a fraction of CD14+ CD8 cells constitutively express IL-10/IL-2 and some express IFNg/TNFa/MIP1b, the potential functional relevance of IL-10/IL-2 production (or other cytokines produced) under different immunological conditions is lacking.
$CD14^{+}CD8^{+}$T-cells have an unusual ability to constitutively produce IL-10 and Il-2 directly *ex vivo* without restimulation; both these cytokines can support the survival and function of T cells responding to their cognate antigen in the liver (*Fioravanti, J Hep 2017, Schurich, JI 2010*). Consistent with the hyper-functionality of

CD14^{+}CD8^{+}T-cells upon TCR engagement we now demonstrate that myeloid reprogramming (marked by acquisition of CD14) can enhance the anti-tumour function of TCR-redirected T cells.

Comment 3. We had asked whether expression of CD71, CD98 and CD36 by CD14+ CD8 cells indicated a metabolically-active state that might separate this CD8+ subset from CD14– CD8 T cells. The authors have not provided information on this point. Instead, they list additional markers of CD14+ CD8 T cells compared with their earlier study (JEM, 2017).

We have now clarified that the increased expression of nutrient transporters like CD71 and CD98 reflects a more metabolically active state of CD14^{+}CD8^{+}T-cells, consistent with their functionally active, proliferative profile. A full analysis of the metabolic re-programming of CD14^{+}CD8^{+}T-cells resulting from myeloid interactions, would be of interest for future studies.

Comment 4. We had questioned the conclusion that CD14+ CD8 cells are long-lived after liver allograft transplantation. While the authors now provide a better FACS plot for detection of donor cells, questions of lifespan and renewal have not been addressed. The authors now state that CD14+ CD8 T cells acquire CD14 protein from other surrounding cells, including mononuclear phagocytes, based on scRNA-Seq and co-culture experiments. It is surprising that donor CD8 cells (and not endogenous CD14+ CD8 T cells) specifically acquire CD14 from surrounding phagocytes and maintain this protein for long periods of time.

We agree this was not clearly explained as we did not mean to imply that CD8^{+}T-cells could acquire CD14 from neighbouring myeloid cells and maintain it for long periods. It was a striking and consistent finding that donor-derived CD8^{+}T-cells expressed even more CD14 than endogenous liver CD8^{+}T-cells, whereas recipient CD8^{+}T-cells could barely acquire it. We have now clarified that the majority of donor CD8^{+}T-cells are lost following transplantation, with large numbers being washed out during pre-transplantation perfusion of the organ. The lower frequency of CD14^{+}CD8^{+}T-cells in perfusates compared to liver tissue digests suggests they are preferentially retained during this procedure. We have shown that the small pool of residual donor CD8^{+}T-cells is highly proliferative (Ki67^{hi}, *Pallett, Burton, JEM 2020*) and now show that CD14 is preferentially acquired on proliferating CD8^{+}T-cells. Donor CD8^{+}T-cells may also preferentially continuously re-acquire CD14 because of their stromal localisation and signals e.g. through their higher expression of chemokine receptors like CXCR3 (*Pallett, Burton JEM 2020*). Recipient T cells may be unable to acquire CD14 due to differential localisation, their inability to acquire high levels of CXCR3 and/or HLA mismatch with donor liver APC.

Comment 6. We had asked about the functional relevance of CD14+ CD8 T cells that express both IFNg and IL-10 in the liver tissue environment. Although the authors refer to a response to another reviewer, it does not address this question.

We have now stressed that CD8^{+}T-cells co-producing IFN-γ and IL-10 have been shown to have enhanced antiviral potential in the liver tissue environment in work from the Iannacone lab (*Fioravanti, J Hep 2017*). We have included a cartoon (Ext.Fig4l) highlighting the highly context-dependent functional potential of CD14^{+}CD8^{+}T-cells. Since CD14^{+}CD8^{+}T-cells are not detectable in mice we have not been able to deplete them to determine a single dominant role in a reductionist model. However, being the sole fraction of CD8^{+}T-cells able to constitutively produce IL-10 and IL-2, the most potent effectors upon TCR engagement, and with their additional capacity to produce chemotactic and hepatoprotective cytokines upon LPS exposure, CD14^{+}CD8^{+}T-cells are an important new population to study in liver homeostasis and disease. For example, we have now shown that myeloid instruction achieved by co-culture with mononuclear phagocytes and stellate cells, produces a population of CD14 expressing TCR-re-directed T cells able to mediate superior anti-tumour function against an HBV-expressing hepatoma cell line. This study focuses on defining the potential for mononuclear phagocytes to re-programme tissue T cells *in vitro* and *in vivo* and their frequency, topology, phenotype and functional profile in human liver; follow-up studies will be needed to explore their roles in different settings.

Referee #4 (Remarks to the Author):

In the revised manuscript, Pallett et al. have elaborated in more detail on the ontogeny and function of CD14+CD8+ T cells compared to the initial version of the manuscript. The authors present a very comprehensive study, have applied challenging and cutting-edge methods and have investigated unique patient samples to characterize this activated tissue-sequestered novel CD8+ T cell population. Still, the question about the biological relevance of CD14+CD8+ T cells remains elusive.

Nevertheless, this is an elegant study providing some novel insights into the immunity at barrier tissues.

Thank you for this positive assessment.

I have two minor remarks:

(1) since CD14+CD8+ T cells are not restricted to the liver (as nicely shown by the authors), a greater emphasize should be made on the discussion of the potential role of CD14+CD8+ T cells in barrier tissues in general;
We have emphasised our finding of $CD14^+CD8^+$T-cells in other sites such as the skin and peritoneal cavity and the need to further explore their role in these and other barrier tissues.

(2) to clarify that CD14 expression is not restricted to CD8+ T cells within the lymphocyte compartment, the data on CD14-expressing CD4+ T cells and NK cells (as provided for the reviewer) should be included in the manuscript.
We have now included these data in Ext.Fig.1e.

Reviewer Reports on the Second Revision:

Referees' comments:

Referee #1 (Remarks to the Author):

I appreciate the authors' extensive efforts to address the questions raised in the prior review. I have no additional concerns.

Referee #3 (Remarks to the Author):

In their manuscript, “Tissue CD14+ CD8+T-cells are reprogrammed by myeloid cells and modulated by LPS”, Pallett & colleagues describe a subset of CD8 T cells that stain for CD14 and other myeloid markers, which can be found in the human liver, skin, and peritoneum. These cells are hyper-active by the metrics presented here, and can be instructed by LPS to perform wound-healing functions upon bacterial exposure.
I have two main concerns about this manuscript, outlined below:
First, a technical concern. The CD14+ T cells described in this paper have elevated expression of every marker that the authors check (i.e., 18 markers), raising the possibility that some of these findings reflect non-specific antibody staining. Since the authors note that CD14 is gained from myeloid cells rather than expressed endogenously, it is possible that CD14+ CD8 T cells have also gained Fc receptor expression from their myeloid partners. In this case, and because the authors do not state that they block for Fc receptor activity include isotype controls in their staining protocols, it is impossible to know whether the toti-positivity observed for CD14+ CD8 T cells reflects their biological hyperactivity, or non-specific Ab-Fc interactions. As a particular example of this, the authors’ finding that CD14+ CD8 T cells are up to 67% positive for IL10 (Fig 3C). In the absence of appropriate controls, this might easily be attributed to non-specific staining.
Second, even if the above were completely resolved, the authors do not provide a compelling argument that these observations are broadly applicable. Since the T cells in question gain their CD14 expression from myeloid cells in a contact-dependent manner, it remains possible that CD14 simply marks T cells that have recently interacted with antigen-presenting cells. The idea that T cells only become fully functional after interactions with APC may simply reflect a well-establish paradigm in immunology.
This is not to dismiss the core observation of the manuscript: that CD14 can be used to identify highly active CD8 T cells that may have interacted with APC. This is relevant to T cell immunologists, especially those working on liver, peritoneum, and skin immunology. However, the claim that CD14-expressing CD8 T cells are of broad biological interest requires a much better mechanistic understanding of CD8 T cell reprogramming by myeloid cells. Although the authors show a contact dependence and a requirement for CXCL12 and LFA1 (a stand-in for contact dependence), insight into the basis for the phenotypic changes described requires analysis of the relevant receptors and signaling pathways. Without this analysis, CD14 may simply mark activated hepatic CD8 T cells, a finding that is likely to be of interest primarily to specialists in the field.

Author Rebuttals to Second Revision:

We have addressed the remaining issues raised by Reviewer 3 as follows:

1. *The technical concern re the possibility of non-specific antibody binding to Fc receptors (FcR) on $CD14^{+}CD8^{+}$T-cells that they could have acquired from myeloid cells:*
   This is a valid concern that we also considered early on in the study. Apologies that we forgot to clarify the methodological steps we took to mitigate against this possibility, as follows:

   a) We did include an Fc blocking step (using the Miltenyi FcR block reagent – catalogue # 130-059-901) during antibody staining; this has now been added into the Methods, line 866-867.
   b) To further avoid this potential issue, we switched early on in our study to using a Miltenyi REA anti-$CD14^{APC}$ antibody which has an Fc region that is mutated to abrogate binding to FcR. Since this actually gave stronger and clearer staining of CD14 on $CD8^{+}$T-cells (rather than abolishing it which would have been expected if there was an issue with non-specific FcR binding) we switched to using it for most experiments, including the representative plots that were already shown in Fig.1a. The use of this REA antibody was already listed in the supplementary antibody table, we have now also specified why it was used in the Methods, page 869-870 and specifically mentioned it in the legend of Fig.1a.
   c) If we had an issue with non-specific antibody binding we would have expected to see increased staining for all markers on $CD14^{+}CD8^{+}$T-cells; however we showed in Ext. data Fig.1c,d that CD45, $\alpha\beta$TCR, and CD3ζ were expressed at comparable levels on $CD14^{+}$ and $CD14^{-}CD8^{+}$T-cells. We had previously included a number of other markers expressed at similar levels on these populations that we had to remove from this revision for space constraints. However, we have now added back in PD-1 expression as another example of an antibody where staining was not increased on $CD14^{+}CD8^{+}$T-cells (added to Fig.3b). Moreover, non-specific FcR binding of antibodies would not be expected to give such variability between antibodies of different specificities but the same isotype/species/flurochrome/manufacturer, e.g. mean 8.9% $CD14^{+}CD8^{+}$T-cells staining with anti-$Ki67^{(PE-Cy7)}$ versus mean 40.5% staining with anti-$CD25^{(PE-Cy7)}$ (Fig.3a and b), both of which are the same isotype subclass antibodies (mouse; IgG1κ).

2. *The biological concern that 'CD14 merely marks out cells that are highly activated as a result of interaction with APC in certain tissues including liver, peritoneum and skin':*
   Firstly, we would counter that this in itself *would be* of broad biological relevance and high utility for understanding human tissue immunity. Secondly, we have shown a lot more than this, including the fact that this interaction and re-programming has a zonal pattern, can be promoted by stromal CXCL12 or LPS and that $CD14^{+}CD8^{+}$T-cell functionality is also selectively altered after exposure to LPS. Our findings additionally have translational applicability since we show myeloid instruction can be used to boost the function of TCR-redirected T cells for adoptive cell therapy. However, we do agree that with this first description of these phenomena many questions remain unanswered, in particular the mechanistic interactions and signalling pathways through which myeloid re-programming of T cells occurs. We have extended the discussion of these caveats, page 12.
