# Supplementary material for: Tissue CD14+CD8+T-cells reprogrammed by myeloid cells and modulated by LPS
Source: Nature. Author manuscript; Available in PMC 2026 Aug 12. (PMC7619353; doi:10.1038/s41586-022-05645-6)
Supplement: Supplementary file 2 [file EMS216202-supplement-Reporting_Summary.pdf]

nature research

Corresponding author(s): Mala K. Maini; Laura J. Pallett
Nature 2018-10-15114D

Last updated by author(s): Nov 3, 2022

Reporting Summary

Nature Research wishes to improve the reproducibility of the work that we publish. This form provides structure for consistency and transparency in reporting. For further information on Nature Research policies, see our Editorial Policies and the Editorial Policy Checklist.

Statistics

For all statistical analyses, confirm that the following items are present in the figure legend, table legend, main text, or Methods section.

n/a	Confirmed	
☐	☒	The exact sample size (n) for each experimental group/condition, given as a discrete number and unit of measurement
☐	☒	A statement on whether measurements were taken from distinct samples or whether the same sample was measured repeatedly
☐	☒	The statistical test(s) used AND whether they are one- or two-sided<br>*Only common tests should be described solely by name; describe more complex techniques in the Methods section.*
☒	☐	A description of all covariates tested
☒	☐	A description of any assumptions or corrections, such as tests of normality and adjustment for multiple comparisons
☐	☒	A full description of the statistical parameters including central tendency (e.g. means) or other basic estimates (e.g. regression coefficient) AND variation (e.g. standard deviation) or associated estimates of uncertainty (e.g. confidence intervals)
☐	☒	For null hypothesis testing, the test statistic (e.g. F, t, r) with confidence intervals, effect sizes, degrees of freedom and P value noted<br>*Give P values as exact values whenever suitable.*
☒	☐	For Bayesian analysis, information on the choice of priors and Markov chain Monte Carlo settings
☒	☐	For hierarchical and complex designs, identification of the appropriate level for tests and full reporting of outcomes
☒	☐	Estimates of effect sizes (e.g. Cohen's d, Pearson's r), indicating how they were calculated

Our web collection on statistics for biologists contains articles on many of the points above.

Software and code

Policy information about availability of computer code

Data collection	Software used for data acquisition: FACSDIVA v8.0.0.1; INSIPIRE v.200.1.388.0; Illumina HiSeq 4000 v.4; xPONENT v.4.2
Data analysis	Software used for data/statistical analysis: FlowJo v9.9.4 or v.10.8.1, Prism v7 and v8, Ideas, Python 3.6, Excel v.16.16.09, Zen v.2.3<br>Imaging mass cytometry: FIJI ImageJ v.1.52; R Studio with imctools, QMIQ and cytomapper. The Liver Zonation Index was calculated by importing relevant tissue section images into ImageJ (Version 1.52) using the imctools plugin<br>Luminex analysis used to generate heat map in Fig.4: custom pipeline Python 3.6: https://github.com/ljpallett/pallettetal_2022_luminex)<br>SMART-Seq2: data aligment STAR v.2.5.1b; quantification htseq-count v.0.10.0; clustering and annotation NormalizeData tool Seurat,<br>Data visualization: seaborn, the statistical visualisation package

For manuscripts utilizing custom algorithms or software that are central to the research but not yet described in published literature, software must be made available to editors and reviewers. We strongly encourage code deposition in a community repository (e.g. GitHub). See the Nature Research guidelines for submitting code & software for further information.

Data

Policy information about availability of data

All manuscripts must include a data availability statement. This statement should provide the following information, where applicable:

- Accession codes, unique identifiers, or web links for publicly available datasets
- A list of figures that have associated raw data
- A description of any restrictions on data availability

Where appropriate all raw data (with a list of associated figures) will be made available on public repositories.

The data analsysed within the main figures of this article are included in this published article (within the supplementary information files). The datasets generated during and/or analysed during the current study are available from the corresponding author on reasonable request. Correspondence and requests for materials should be addressed to MKM or LJP.

Field-specific reporting

Please select the one below that is the best fit for your research. If you are not sure, read the appropriate sections before making your selection.

☒ Life sciences ☐ Behavioural & social sciences ☐ Ecological, evolutionary & environmental sciences

For a reference copy of the document with all sections, see nature.com/documents/nr-reporting-summary-flat.pdf

Life sciences study design

All studies must disclose on these points even when the disclosure is negative.

Sample size	For the determination of frequency of CD14-expressing CD8 T cells in the intrahepatic compartment, preliminary data obtained from our previous study (Pallett LJ. JEM 2017) gave an effect size of 1.38 when comparing blood and liver samples. Using this, an alpha level of 0.05, and power of 0.8 gave us a required sample size of 10 study participants in each group. So using opportunistic sampling of "waste" material from transplant/resection/explant surgery at the Royal Free Hospital for clinical or diagnostic purpose we ensured we obtained a least this number. We continued to collect and analyse consecutive samples made available to the lab (where the isolated cell yield allowed) for further phenotypic and functional assessment of this subpopulation.<br><br>To ensure transparency in our data, all points from individuals sampled obtained have been shown in figures and/or data distribution depicted using violin plots. Sample sizes are given for each figure throughout the paper in the corresponding legends. Sample size varies from experiment to experiment depending on tissue/sample availability.
Data exclusions	Prior to inclusion in this study, human participants were excluded prior to sample collection if they were HIV-seropositive. Once biological samples were processed and stained for flow cytometric analysis no data point was excluded. All data obtained during this study has been presented within the figures.
Replication	For the phenotypic and functional assessment of CD14 expressing T cells by luminex, flow cytometry or imaging cytometry, experiments using human biological samples, each study participant / sample source was processed and analysed independently. Thus the experimental techniques used (leukocyte isolation, monoclonal antibody staining, flow cytometry, and down-stream analysis) were replicated numerous times on independent days.<br><br>Experimental variation has been depicted graphically with all data points/study participants displayed (as dots) and bars representing the mean/median (indicated as appropriate). Where appropriate when biological samples are linked, for example if we received blood and tissue samples from the same study participant we have represented these as paired data (with appropriate statistical testing).
Randomization	No randomisation was used in this study. All participants were pseudo-anonymised and sampled ad hoc.
Blinding	Not applicable to this study.

Reporting for specific materials, systems and methods

We require information from authors about some types of materials, experimental systems and methods used in many studies. Here, indicate whether each material, system or method listed is relevant to your study. If you are not sure if a list item applies to your research, read the appropriate section before selecting a response.

Materials & experimental systems

n/a	Involved in the study	
☐	☒	Antibodies
☐	☒	Eukaryotic cell lines
☒	☐	Palaeontology and archaeology
☒	☐	Animals and other organisms
☐	☒	Human research participants
☒	☐	Clinical data
☒	☐	Dual use research of concern

Methods

n/a	Involved in the study	
☒	☐	ChIP-seq
☐	☒	Flow cytometry
☒	☐	MRI-based neuroimaging

Antibodies

Antibodies used	Detailed specification (including clone, catalogue numbers and dilutions) of all antibodies used throughout this study have been

Antibodies used	included in published article.<br>For flow cytometry these are detailed in Supplementary Table 1<br>For Hyperion, imaging mass cytometry these are included in Supplementary Table 2<br>For confocal imaging these are included in the corresponding section of the Methods
Validation	All antibodies were purchased from well established manufacturers and were validated by the vendor for species and target. e.g. BD biosciences, Biolegend in Knock-out/knock-in primary model systems to ensure biological accuracy in ISO 9001 certified facilities. Side-by-side lot comparisons are performed.<br><br>Details of antibody clones have been included for cross-referencing of manufacturing company specification/validation processes. We further validated antibodies by titration to optimal concentrations and by using positive controls where possible (e.g. using populations known to express a certain marker or by polyclonal stimulation).<br><br>Fluorescence minus one stains or unstimulated wells/DMSO control wells were used to define gates in Flowjo for all FACS assays . Negative controls were included in each run and positive controls where possible (PBMCs).

Eukaryotic cell lines

Policy information about cell lines

Cell line source(s)	Phoenix-AMPHO: purchased from ATCC (Phoenix-AMPHO (ATCC® CRL-3213™)<br>HepG2-preS1: donated by Prof Antonio Bertoletti (Singapore)
Authentication	Phoenix-AMPHO: This cell line was purchased from a well established company and was validated by the vendor.<br>We did not perform further cell line authentication.<br><br>HepG2-preS1: This cell line was generated to constitutively process and present HBsAg peptide to antigen-specific T cells - as described here: https://pubmed.ncbi.nlm.nih.gov/28614795/.<br>As shown in Fig.3 this line has been validated through the use of TCR-redirected HBsAg-specific T cells, that functionally respond to (via the production of cytokines/chemokines) and lyse the cell line.
Mycoplasma contamination	Both cell lines used in this study were regularly tested throughout for mycoplasma contamination by PCR (EZ-PCR Mycoplasm Kit, Biological Industries).
Commonly misidentified lines<br>(See ICLAC register)	No commonly misidentified cell lines were used in this study.

Human research participants

Policy information about studies involving human research participants

Population characteristics	Each study participant provided written informed consent prior to inclusion in the study, with the storage of any samples/ clinical information conforming to the requirements of the Data Protection Act 1998 and the Human Tissue Act 2004.<br><br>Liver samples were obtained from the non-diseased distal 'healthy' margins of colorectal metastases or hepatocellular carcinoma resections/explants, or perfusion fluid (perfusates) obtained from healthy livers used for solid-organ transplantation. Ascitic fluid samples were obtained from study participants with cirrhosis or IVC obstruction requiring an ascitic tap as part of their routine clinical care. Lymph node and splenic samples were obtained from deceased donors (otherwise healthy participants) through the NHSBT transplant pathway surplus to requirements during solid organ transplantation used primarily for HLA-typing.<br><br>All study participants were excluded on the basis of HIV-seropositivity and lack of capacity for consent.<br>All study participants were over the age of 18.
Recruitment	Recruitment of patients for the provision of biological samples was carried out by the surgical team making samples available to the laboratory via the Tissue for Patient Benefit Initiative (via Amir Gander, RFH, London, UK). Written, informed consent was obtained from each study participant before enrollment.<br><br>Recruitment of volunteers for the in vivo skin biopsy/blister experiments took place within UCL using poster advertising. Study approved by UCL Research Ethics Committee (ref: 10527/001). Informed consent was obtained from each study participant before enrollment. Additional health screening was done before allowing study participants to take place in the in vivo exposure skin blister study.<br><br>Recruitment of volunteers for the ascitic fluid sampling took place at the Hospital Clinic de Barcelona by co-authors from the manuscript. Informed consent was obtained from each study participant before enrollment.
Ethics oversight	Ethical oversight for liver/splenic/lymph node samples: Tissue Access for Patient Benefit Initiative (TAPb) at The Royal Free Hospital, Hampstead, London (approved by the UCL-Royal Free Hospital BioBank Ethical Review Committee references: 11/ WA007 and 16/WA/0289).<br><br>Ethical oversight for ascitic fluid: Royal Free Hospital, London (approved by the UCL Research Ethics Committee reference:

15/LO/0800), the Hospital Clínic de Barcelona (approved by Ethics Committee Hopsital Clínic de Barcelona, Committee reference: 2019-0267) or the Royal London Hospital (approved by the London Bridge Research Ethics Committee reference 17/LO/0266)

Ethical oversight for skin samples: UCL Research Ethics Committee (ref: 10527/001)

Ethical oversight for the tissue samples used for imaging cytometry: Freiburg (approved by the ethics committee of the Albert-Ludwigs-University; Freiburg reference: #21-1372)

Note that full information on the approval of the study protocol must also be provided in the manuscript.

Flow Cytometry

Plots

Confirm that:

☒ The axis labels state the marker and fluorochrome used (e.g. CD4-FITC).

☒ The axis scales are clearly visible. Include numbers along axes only for bottom left plot of group (a 'group' is an analysis of identical markers).

☒ All plots are contour plots with outliers or pseudocolor plots.

☒ A numerical value for number of cells or percentage (with statistics) is provided.

Methodology

Sample preparation

Human biological samples:

For the isolation of human intrahepatic leukocytes (IHL) from resected/explanted material, tissue was cut into small pieces and incubated at 37ºC for 30 min in HBSS (Life Technologies; ThermoFisher Scientific) containing 0.01% collagenase IV (ThermoFisher Scientific) and 0.001% DNaseI (Roche; Sigma Aldrich; Merck). Mechanical disruption was then performed using a GentleMACS (Miltenyi Biotech) with debris removed by filtration through a 70 µM filter (Greiner). Parenchymal cells were removed by centrifugation on a 30% Percoll gradient (GE Healthcare; VWR) followed by a further leukocyte isolation by density centrifugation using a Pancoll gradient (PAN Biotech). For the isolation of IHL from perfusion liquid (perfusates): first the perfusate was concentrated by centrifugation. Concentrated cells were resuspended in RPMI-1640 (Life Technologies; ThermoFisher Scientific) and isolated by density centrifugation on a Pancoll gradient.

For the isolation of human leukocytes from the skin, spleen and lymph nodes, tissue was first cut into small pieces and washed with an EDTA-containing buffer (2mM EDTA [Sigma Aldrich; Merck] + 0.5% heat inactivated fetal bovine serum [HI-FBS; Sigma Aldrich; Merck] in 1x phosphate buffered saline [PBS; Life Technologies; ThermoFisher Scientific]). For the skin an additional enzymatic digestion (using the Whole Skin Digestion kit; Miltenyi Biotech) was required overnight. Mechanical disruption was performed using the barrel of a 5 mL sterile syringe to pass the cells through a 70 µM filter. Concentrated cells were then resuspended, and leukocytes isolated by density centrifugation on a Pancoll gradient. Leukocytes obtained from the spleen and lymph nodes were further subjected to a red blood cell lysis (Biolegend), on ice, prior to use. All intrahepatic, skin, splenic and lymphoid cell samples were used immediately.

For the isolation of human leukocytes from ascitic fluid, the fluid drained from the patient was concentrated by centrifugation and washed twice with RPMI-1640. Where necessary leukocytes were further subjected to a red blood cell lysis prior to use and frozen in 10% DMSO (Sigma Aldrich; Merck)-HI FBS if not used immediately.

Where possible peripheral blood was obtained from the same study participants for comparison. Peripheral blood mononuclear cells (PBMC) were isolated from heparinised peripheral blood by density centrifugation using a Pancoll gradient.
.

Instrument

BD Bioscience LSR-X20-SORP

Software

FlowJo v.9.9.4, Python 3.6

Cell population abundance

Sample purity, where appropriate, was confirmed by flow cytometry. ~300 sorted cells were re-analysed on flow cytometer to confirm purities >98%.

Gating strategy

The flow cytometry gating strategy used to defined CD14-expressing T-cells used in this study is shown in the Extended Data Figures. For all samples strict exclusion criterion were used during gating to exclude debris, dead cells (using an Invitrogen live dead protein stain), CD45negative cells and the following lymphocyte populations (NK cells, B cells, CD4 T cells and non-classical CD56+CD3+ T cells). Where indicated CD161hiVa7.2 MAITs were further excluded.

☒ Tick this box to confirm that a figure exemplifying the gating strategy is provided in the Supplementary Information.
