# Supplementary material for: Tissue CD14+CD8+T-cells reprogrammed by myeloid cells and modulated by LPS
Source: Nature. Author manuscript; Available in PMC 2026 Aug 12. (PMC7619353; doi:10.1038/s41586-022-05645-6)
Supplement: Supplementary file 3 [file EMS216202-supplement-Supplementary_information.pdf]

nature portfolio

https://doi.org/10.1038/s41586-022-05645-6

Supplementary information

Tissue $CD14^{+}CD8^{+}$ T cells reprogrammed by myeloid cells and modulated by LPS

In the format provided by the authors and unedited

Supplementary Table 1

Detailed specifications of monoclonal antibodies used for flow cytometric analyses (D.F. dilution factor used)

Antigen	Fluorochrome	Company	Clone	Catalogue No.	D.F.
CD45	BV805	BD Bioscience	HI100	564914	1:200
CD3	BV711	Biolegend	OKT3	317328	1:100
CD3	BUV395	BD Biosicence	UCHT1	563546	1:100
CD8	BV785	Biolegend	RPA-T8	301046	1:100
CD8	AlexaFluor700	Thermo Scientific	OKT8	56-0086-42	0.5:100
CD8	APC	Thermo Scientific	OKT8	17-0086-41	1:100
CD4	APC/Cy7	Biolegend	RPA-T4	300518	0.5:100
CD4	BUV395	BD Bioscience	SK3	563550	1:100
CD56	PE/Cy7	BD Bioscience	NCAM16.2	335826	1.5:100
CD56	ECD	Beckman Coulter	N901	A82943	1:100
CD56	PE/Dazzle594	Biolegend	QA17A16	392410	0.5:100
CD19	BV786	BD Bioscience	SJ25c1	563325	1:100
CD19	APC/Cy7	BD Bioscience	SJ25C1	557791	1:100
CD14	V500	BD Bioscience	M5E2	561391	2:100
CD14	BV510	Biolegend	M5E2	301842	1:100
CD14	APC	Miltenyi Biotech	REA599	170-078-099	1:100
CD14	APC/Cy7	Biolegend	M5E2	301820	3:100
CD14	PE	Biolegend	63D3	301850	1:100
αβTCR	PerCP/Cy5.5	Biolegend	IP62	306724	1:100
αβTCR	APC/Cy7	Biolegend	IP62	306728	2:100
αβTCR	PE				
CD57	eFluor450	Thermo Scientific	TB01	48-0577-42	1:100
TLR2	BV711	BD Bioscience	11G7	742770	2:100
TLR2	FITC	Thermo Scientific	TL2.1	11-9922-42	3:100
TLR4	BV421	BD Bioscience	HTA125	743392	4:100
TLR4	PE	Biolegend	HTA125	312806	2:100
TLR4	FITC	Thermo Scientific	HTA125	MA1-22766	3:100
CD36	APC	Biolegend	5-271	336208	1:100
CD98	FITC	Biolegend	MEM-108	315603	2:100
CD71	PerCP/Cy5.5	Biolegend	CY1G4	334114	2:100
HLA-DR	V500	BD Bioscience	G46-6	561224	1:100
CD161	PE	Miltenyi Biotech	191B8	130-092-677	2:100
CD161	APC	Miltenyi Biotech	191B8	130-098-908	2:100
Vα7.2	FITC	Biolegend	3C10	351704	3:100
Vα7.2	BV785	Biolegend	3C10	351722	1:100
Vα7.2	BV605	Biolegend	3C10	351720	1:100
CD38	PE/Dazzle594	Biolegend	HIT2	303538	0.5:100
CD38	BV605	Biolegend	HIT2	303532	0.5:100
CD25	APC	Biolegend	BC96	302610	3:100
CD25	FITC	BD Bioscience	2A3	345796	3:100
CD25	PE/Cy7	Thermo Scientific	BC96	25-0259-42	2:100
PD-1	PE	Biolegend	EH12.2H7	329906	1:100
LAG3	PE/Cy7	Thermo Scientific	3DS223H	25-2239-42	2:100
CD39	BV421	Biolegend	A1	328214	2:100
CD39	PE/Cy7	Biolegend	A1	328212	1:100
CD73	FITC	Biolegend	AD2	344016	1:100
ICOS	BV605	Biolegend	C398.4A	313538	1:100
GITR	BV711	Biolegend	108-17	371212	2:100
CD49a	BUV395	BD Bioscience	SR84	742363	1:100
CD49a	FITC	Biolegend	TS2/7	328308	1:100

CD49b	APC	Biolegend	TS2/7	328313	2:100
CD49b	FITC	Biolegend	TS2/7	328308	2:100
CXCR3	PerCP/Cy5.5	BD Bioscience	IC6/CXCR3	560832	2:100
CXCR4	BV605	Biolegend	12G5	306521	1:100
CD69	PE/Dazzle594	Biolegend	FN50	310942	0.5:100
CD69	BV605	Biolegend	FN50	310938	0.5:100
CD103	BV711	BD Bioscience	Ber-ACT8	563162	2:100
CD103	BV605	BD Bioscience	Ber-ACT8	743652	2:100
CD3ζ(CD247)	PE	Thermo Scientific	6B10.2	12-2479-82	1:100
IL-10	AlexaFluor488	Biolegend	JES3-9D7	501411	4:100
IFNγ	V450	BD Bioscience	B27	560371	2:100
TNF	APC/Cy7	Biolegend	Mab11	502944	1:100
IL-2	PerCP/eFluor710	Thermo Scientific	MQ1-17H12	46-7029-42	2:100
IL-2	BB700	BD Bioscience	MQ1-17H12	566406	1:100
MIP1β	PE	BD Bioscience	D21-1351	561120	1:100
Ki67	PE/Cy7	Thermo Scientific	20Raj1	25-5699-42	3:100
Foxp3	BV421	Biolegend	206D	320124	4:100
Foxp3	V450	BD Bioscience	2396A/E7	561182	4:100
Foxp3	AlexaFluor660	Thermo Scientific	236A/E7	48-4777-42	3:100
CTLA-4	PerCP/eFluor710	Thermo Scientific	14D3	46-1529-42	2:100
CTLA-4	PE	BD Bioscience	BNI3	555853	4:100
HLA-A2	PE/Cy7	Biolegend	BB7.2	343314	3:100
HLA-A2	FITC	BioRad	BB7.2	MCA2090	3:100
HLA-A3	PE	Thermo Scientific	GAP.A3	12-5754-42	3:100
Streptavidin	PE	BD Biosciences		554061	1:1000
IL-8	PE/Cy7	Biolegend	E8N1	511416	2:100
CXCL1	eFluor660	Thermo Scientific	KTYFLF	50-7515-42	2:100
CCL2	AF700	BioTechne	2D8	NBP2-2215A700	3:100
MD-2	FITC	abcam	18H10	Ab106105	2:100
Murine TCRβ	PE/Cy7	Thermo Scientific	H57-597	25-5961-82	1:100

Supplementary Table 2

Detailed specifications of monoclonal antibodies used for the Hyperion Imaging Mass Cytometry (D.F. dilution factor used)

Channel	Epitope	Manufacturer	Catalog	Clone	Dilution
115 In	HLA-DR	Abcam	ab176408	TAL 1B5	1:400
144 Nd	CD69	Abcam	ab234512	EPR21814	1:100
145 Nd	CD3	CST	85061S	D7A6E	1:200
151 Eu	CD31	Fluidigm	3151025D	EPR3094	1:200
156 Gd	CD4	Abcam	ab181724	EPR6855	1:200
157 Gd	CYP1A2	Novus	NB600-1490	D15	1:200
159 Tb	CD68	Biolegend	916104	KP1	1:1600
161 Dy	Iba1	Abcam	ab220815	EPR16588	1:1600
162 Dy	CD8	Biolegend	372902	C8/144B	1:800
174 Yb	CD14	ProteinTech	60253-1-Ig	2C1D9	1:800
175 Lu	CD34	Abcam	ab198395	EP373Y	1:400
176 Yb	CK7	Origene	BM6003P	RCK105	1:400
194 Pt	HH3	CST	4499BF	D1H2	1:100
